# Rapid Iododeboronation with and without Gold Catalysis: Application to Radiolabelling of Arenes

**DOI:** 10.1002/chem.201704534

**Published:** 2017-12-14

**Authors:** Stacey Webster, Kerry M. O'Rourke, Conor Fletcher, Sally L. Pimlott, Andrew Sutherland, Ai‐Lan Lee

**Affiliations:** ^1^ Institute of Chemical Sciences, School of Engineering and Physical Sciences Heriot–Watt University Edinburgh EH14 4AS UK; ^2^ WestCHEM, School of Chemistry, The Joseph Black Building University of Glasgow Glasgow G12 8QQ UK; ^3^ West of Scotland PET Centre Greater Glasgow and Clyde NHS Trust Glasgow G12 0YN UK

**Keywords:** aryl iodides, gold catalysis, iodination, radiochemistry, radiopharmaceuticals

## Abstract

Radiopharmaceuticals that incorporate radioactive iodine in combination with single‐photon emission computed tomography imaging play a key role in nuclear medicine, with applications in drug development and disease diagnosis. Despite this importance, there are relatively few general methods for the incorporation of radioiodine into small molecules. This work reports a rapid air‐ and moisture‐stable *ipso*‐iododeboronation procedure that uses NIS in the non‐toxic, green solvent dimethyl carbonate. The fast reaction and mild conditions of the gold‐catalysed method led to the development of a highly efficient process for the radiolabelling of arenes, which constitutes the first example of an application of homogenous gold catalysis to selective radiosynthesis. This was exemplified by the efficient synthesis of radiolabelled *meta*‐[^125^I]iodobenzylguanidine, a radiopharmaceutical that is used for the imaging and therapy of human norepinephrine transporter‐expressing tumours.

## Introduction

Aryl iodides are versatile key building blocks in organic synthesis and have found widespread applications in areas such as cross‐coupling reactions and the generation of free‐radical intermediates.^1^ In addition to their applications in synthesis, aryl iodides are also found in natural products and pharmaceutically important compounds.^2^, ^3^ More recently, radiolabelled aryl and heteroaryl iodides have been increasingly reported in medical applications; in particular, in single‐photon emission computed tomography (SPECT) imaging for drug development and the clinical diagnosis of disease, and in targeted radionuclide therapy.^4^

As a result of these applications, much effort has gone into developing methods for the efficient synthesis of aryl iodides.^5^ One such method is the *ipso*‐substitution of arylboronic acids by using *N*‐iodosuccinimide (NIS), which was developed by Olah.^6^ However, one limitation of this uncatalysed method is the substrate scope: deactivated arylboronic acids with electron‐withdrawing substituents performed poorly, even after extended reaction times. For this reason, several base‐ or phase‐transfer‐mediated^7^ and Cu‐catalysed7c, 8, 9 *ipso*‐iodination reactions of boronic acids have emerged in recent years. Despite the much‐improved substrate scopes afforded by these recent developments, there are still limitations, such as the need for base/additives, environmentally damaging solvents, or long reaction times.^10^ Therefore, improvement is still required, especially if this transformation is to find widespread utility in fields such as total synthesis, material science, and medical imaging.

**Scheme 1:**
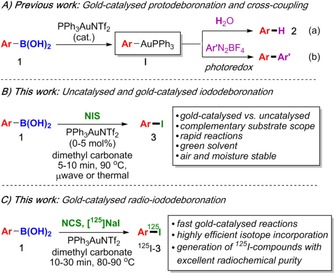
A) Gold‐catalysed protodeboronation/cross couplings. B) Gold‐catalysed and uncatalysed iododeboronation. C) Gold‐catalysed radioiododeboronation.

Our interest in this area arose from our previous work on gold catalysis;^11^ in particular, the mild gold‐catalysed^12^ proto‐ and deuterodeboronations^13^ and cross‐couplings (Scheme 1 A (a) and (b), respectively).^14^ We hypothesised that the organogold intermediate **I** 15 should react with NIS to rapidly form iodoarenes **3**. Therefore, our aims were to 1) compare the gold‐catalysed and uncatalysed reactions to ascertain whether gold catalysis can be used to overcome some of the substrate scope limitations of the uncatalysed reaction, 2) develop a much faster iodination protocol, both catalysed and uncatalysed, 3) utilise environmentally friendly solvents to significantly improve the practicality of the reaction (the original reaction times were 1.5–25 h in acetonitrile),^6^ and 4) to apply the methodology to radioiododeboronations.

Although there are early isolated reports of unselective gold‐mediated radioiodinations^16^ and a more recent elegant use of stoichiometric gold substrates for radiofluorinations,^17^ as far as we are aware, there are no examples of selective radioiodinations^5^ or radiofluorinations^18^ that use homogenous gold catalysis. Therefore, one of the main aims of this work was to demonstrate the first such application of gold catalysis to radiosynthesis. To the best of our knowledge, gold catalysis has never been used for iododeboronations, even in cold (nonradiolabelled) procedures.^19^ However, the groups of Wang and Frontier have reported mechanistically distinct iodinations of arenes through the gold‐catalysed activation of NIS for electrophilic aromatic substitutions.^20^, ^21^ Therefore, another challenge was to develop the intended *ipso*‐iododeboronation in a selective manner and avoid any over‐iodination, which would be caused by electrophilic aromatic substitutions.

Herein, we report a rapid *ipso*‐substitution of arylboronic acids by using NIS in the environmentally friendly solvent dimethyl carbonate (DMC)^22^ under microwave heating^23^ or thermal heating (Scheme 1 B). Both the gold‐catalysed and uncatalysed reactions were investigated concurrently, and we demonstrated that Au^I^ catalysis can be used to greatly improve the yields in cases where the uncatalysed reaction is poor. Conversely, the uncatalysed reaction is often successful in cases where gold catalysis fails, so both protocols complement each other nicely. We also describe the use of this mild and general transformation for the radioiodination of arenes (Scheme 1 C). Incorporation of the ^125^I radioisotope was fast and highly efficient, which allowed the preparation of the radiopharmaceutical *meta*‐[^125^I]iodobenzylguanidine in high molar activity and excellent radiochemical purity.

## Results and Discussion

We initiated our studies by carrying out a solvent screen on the gold‐catalysed iododeboronation of arylboronic acid **1 a** (Table 1). Pleasingly, the expected iododeboronation product **3 a** was observed as the major product in various solvents, although the over‐iodination product **4 a**, which resulted from electrophilic aromatic substitution of gold‐activated NIS,20a was also observed as a minor product. In addition, the protodeboronation product **2 a** was also observed when the reaction was carried out in toluene (entry 4), and it is unsurprisingly the main product of the reaction performed in water (entry 8). The two best results were obtained in chloroform (entry 2) and dimethyl carbonate (entry 3); therefore, dimethyl carbonate was taken forward for optimisation owing to its “green” credentials.^22^

**Table 1:** Solvent and temperature screen.

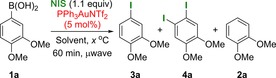				
Entry^[a]^	Solvent	*T* [°C]	Yield [%]^[b]^	**3 a**/**4 a**/**2 a** ^[c]^
1	CDCl_3_	70	60	86:14:0
2	CDCl_3_	90	77	93:7:0
3	DMC^[d]^	90	80	93:7:0
4	toluene	90	77	59:23:18
5	THF	90	50	93:7:0
6	acetone	90	21	complex mixture of products
7	dioxane	90	80	87:13:0
8	water	90	–	mainly **2 a**

[a] Reaction was carried out on a 0.1 mmol scale. Solvents were not anhydrous. [b] Combined yield of **3 a**+**4 a**+**2 a**. [c] Determined by ^1^H NMR analysis. [d] DMC=dimethyl carbonate.

With an eco‐friendly solvent in hand, our next aim was to significantly reduce the reaction times. To this end, the gold‐catalysed iododeboronation of arylboronic acid **1 b** was investigated by using various times (2–5 min), temperatures (70–100 °C), and equivalents of NIS (Table 2). Higher temperatures (entries 1 vs. 2) and a lower equivalent of NIS (entries 3 vs. 5) reduced the amount of unwanted over‐iodination product **4 b**. The conditions shown in entry 6 (90 °C, 5 min, 1.0 equiv of NIS) produced the best compromise between yield and **3 b**/**4 b** selectivity, and therefore, they were taken forward as the optimal conditions.

**Table 2:** Temperature, time, and equivalents screen.

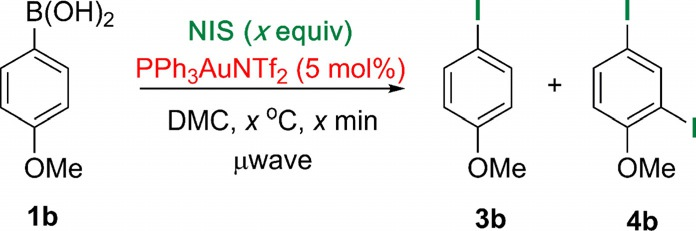					
Entry^[a]^	*T* [°C]	*t* [min]	NIS equiv	Yield [%]^[b]^	**3 b/4 b** ^[c]^
1	70	10	1.1	93	75:25
2	90	10	1.1	100	86:14
3	100	5	1.1	91	91:9
4	100	2	1.1	99	90:10
5	100	5	1.0	88	95:5
**6**	**90**	**5**	**1.0**	**92**	**95:5**

[a] Reaction carried out on 0.1 mmol of **1 b**. [b] Combined yield of **3 b**+**4 b**. [c] Determined by ^1^H NMR analysis.

It is worth noting that the reaction is not sensitive to air or moisture, and thus, it is a very practical, as well as fast, procedure. In fact, wet solvent enhanced the yields for the gold‐catalysed reaction (see the Supporting Information).^13^

Next, the substrate scope of the gold‐catalysed and uncatalysed reactions were investigated under these optimised conditions (Table 3). Both the catalysed and uncatalysed reactions were run concurrently for all substrates to ascertain whether gold catalysis offered any advantage over the uncatalysed reaction or vice versa. For strongly electron‐rich arylboronic acids **1 a**–**b**, the catalysed reaction gave a better combined yield but often poorer selectivity for the desired *ipso*‐iodination (**3**) versus over‐iodination (**4**) products (18:1 and 12:1 for catalysed reaction of **1 a** and **b**, respectively, vs. >20:1 for uncatalysed). Nevertheless, we observed that thermal heating can be used to improve the selectivity in the catalysed reaction (**3 b/4 b**, >20:1); furthermore, over‐iodination was not a problem with less electron‐rich substrates (**1 c**–**l**). This trend was expected, as very electron‐rich aryl substrates are more likely to undergo competitive electrophilic aromatic substitution than the less electron‐rich counterparts. With mildly electron‐rich arylboronic acid **1 d**, the gold‐catalysed reaction was significantly more efficient (100 vs. 62 %) and clean (inseparable side‐product observed in uncatalysed reaction). However, the presence of a phenolic proton in the substrate **1 e** caused both catalysed and uncatalysed reactions to produce a significant amount of a protodeboronated side product **2 e**.^24^ However, THP‐protected phenol substrate **1 f** was tolerated in the uncatalysed reaction, and the acid‐sensitive THP group remained intact during the iododeboronation reaction to yield product **3 f** in 60 % yield. Therefore, for electron‐rich boronic acids, the gold‐catalysed reaction generally provided higher yields, but the lower yielding uncatalysed reaction may arguably still be preferred owing to its cheaper cost.

**Table 3:** Substrate scope.

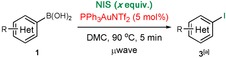
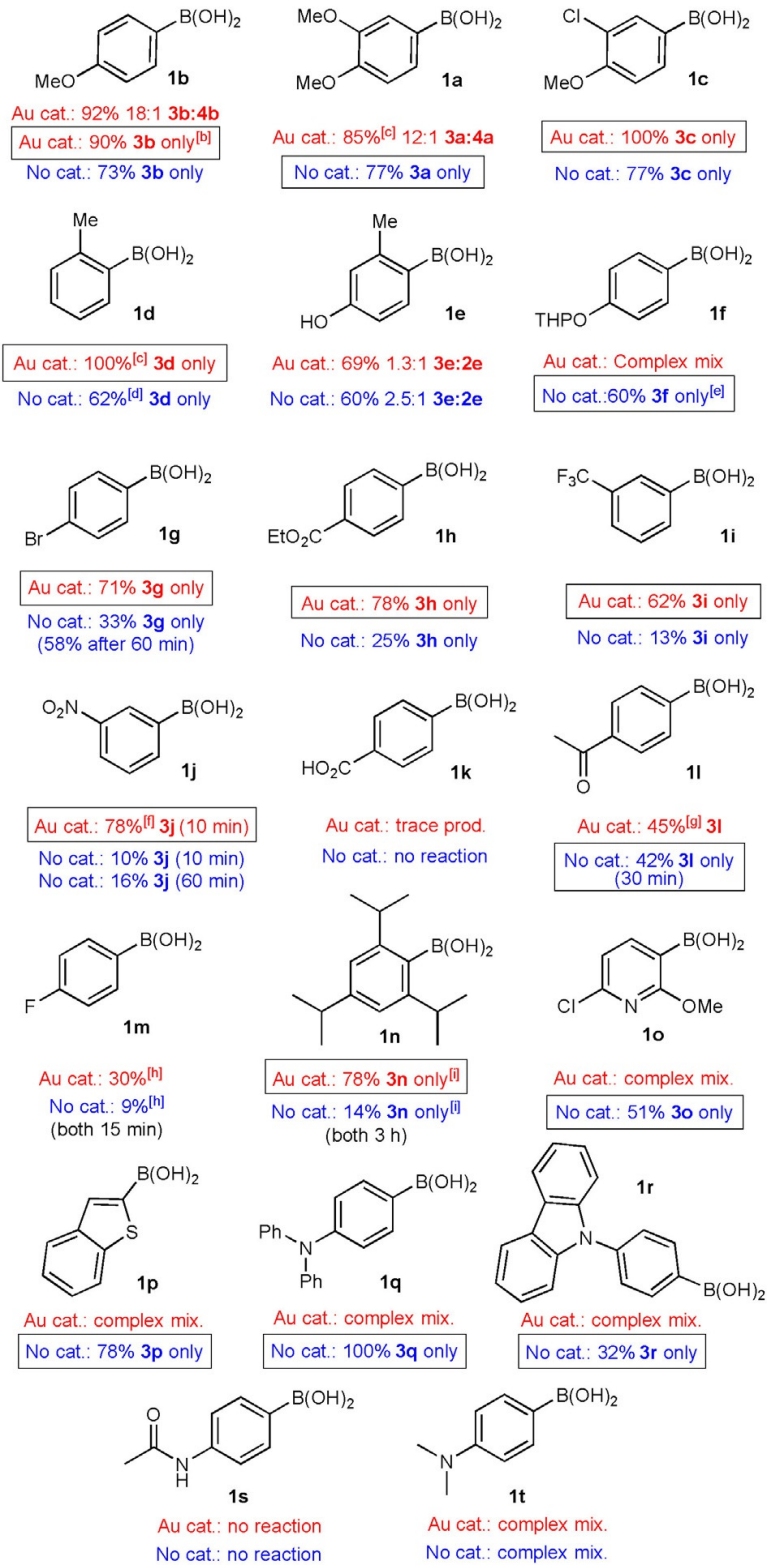

[a] Yield of isolated product. [b] Thermal heating. [c] Temperature increased to 100 °C. [d] Inseparable unidentified side product observed. [e] 4 Å molecular sieves added. [f] 50 % yield after 5 min. [g] 1:1 ratio of **3 l** and α‐iodination product. [h] 1:1 ratio of **3 m** and homocoupled product 4,4′‐difluoro‐1,1′‐biphenyl. [i] Carried out with 1.1 equivalents of NIS.

However, a different pattern emerged for electron‐poor and sterically demanding arylboronic acid substrates (Table 3, **1 g**–**l**). These deactivated species reacted extremely sluggishly in the uncatalysed reaction and provided poor yields, even after extended reaction times. To our delight, gold catalysis provided a significant improvement in the yields as well as reaction times. For example, aryl iodides **3 g**, **h**, and **i** were formed in 71, 78, and 62 % yields, respectively, after five minutes in the presence of PPh_3_AuNTf_2_, whereas only 33, 25, and 13 % yields were obtained without the catalyst. The comparison was even starker with the nitro‐substituted substrate **1 j**: product **3 j** was furnished in 78 % yield after ten minutes under gold catalysis, whereas the uncatalysed reaction provided a very poor 10 % yield. Extending the reaction time to one hour did not significantly improve the yield of product **3 j** in the absence of the catalyst (16 %). The free carboxylic acid **1 k** was not tolerated under the reaction conditions; in fact, substrates that bear acidic protons (e.g., **1 e**, **k**) were generally observed to be detrimental to the reaction success. The acetyl functionality in substrate **1 l** was tolerated in the uncatalysed reaction, although the desired product **3 l** was yielded in a modest 42 % after 30 min. In contrast, the faster gold‐catalysed reaction yielded a 1:1 ratio of the desired product **3 l** and the undesired α‐iodination product. The fluoro‐substituted arylboronic acid **1 m** reacted sluggishly and produced a significant amount of the homocoupling product (4,4′‐difluoro‐1,1′‐biphenyl) in both the gold‐catalysed and uncatalysed reactions. Pleasingly, the extremely sterically demanding substrate **1 n** underwent iododeboronation smoothly in the presence of the gold catalyst to give the desired product **3 n** in a good yield of 78 % (vs. 14 % uncatalysed yield), albeit with a longer reaction time of three hours to account for the steric hindrance. For electron‐poor and sterically hindered arylboronic acids, the uncatalysed reaction was generally low yielding and extremely sluggish, and gold catalysis could be used to significantly improve the reaction times and yields.

The opposite pattern emerged for heterocyclic and N‐containing arylboronic acid substrates (Table 3, **1 o**–**r**). Both heterocyclic boronic acids **1 o** and **p** produced a complex mixture of products under gold catalysis, but they iododeboronated cleanly under uncatalysed conditions to give 51 and 78 % of the desired aryl iodides, respectively. Similarly, amine‐substituted arylboronic acids **1 q** and **r** provided a complex mixture under gold catalysis, but they were successfully iododeboronated under uncatalysed conditions to give **3 q** and **r** in 100 and 32 % yield, respectively. The complex mixture that resulted from gold catalysis is most likely due to over‐iodination20a of these highly electron‐rich aryl substrates. Unsurprisingly, the more electron‐rich amine in **1 t** also resulted in a complex mixture, but this time, under both the catalysed and uncatalysed procedures. The amide functionality in substrate **1 s** seemed to shut the reaction down under both conditions. Although there are some limitations to the type of N‐substituents that are tolerated, the uncatalysed procedure was generally preferred for heterocyclic and N‐substituted arylboronic acids.

**Figure 1:**
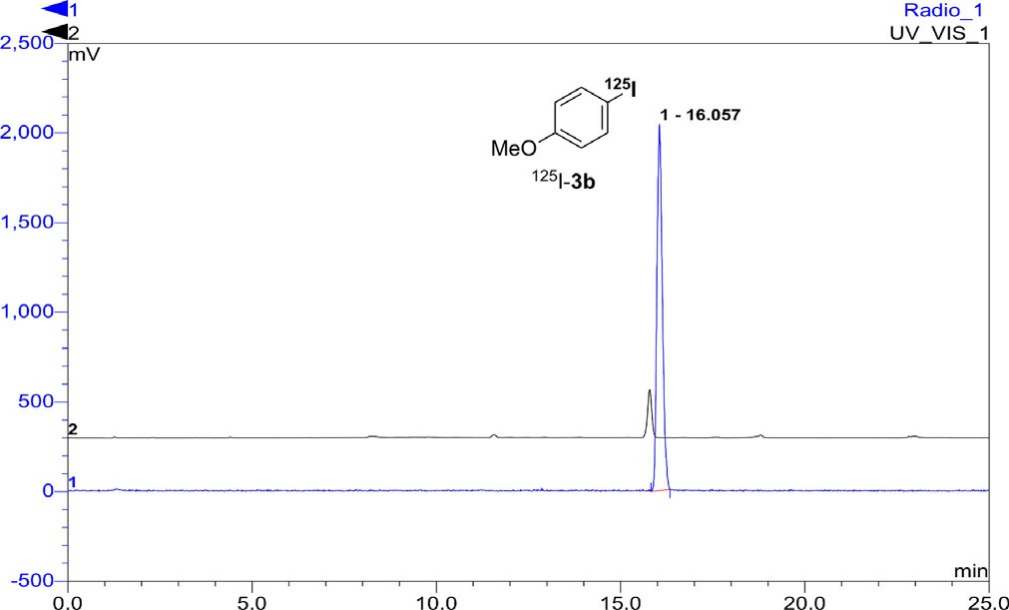
Chromatograph obtained by analytical radio‐HPLC (blue) of the reaction mixture from the radioiodination of boronic acid **1 b**, which shows a quantitative yield of radioiodide incorporation. An overlay of the UV/Vis HPLC trace (black) of product **3 b** is also shown.

As our studies demonstrated that gold‐catalysed *ipso*‐substitution of arylboronic acids by using NIS could be performed rapidly and efficiently under mild conditions, we were keen to investigate the application of this method into the radioiodination of arenes. Radioactive iodine is normally supplied in the form of NaI; therefore, initial studies involved the iodination of 4‐methoxybenzeneboronic acid (**1 b**) with NIS, which was prepared in situ by pre‐stirring NaI and *N*‐chlorosuccinimide (NCS).^25^ Under our standard gold‐catalysed reaction conditions, the desired product **3 b** was formed in 80 % yield. These general conditions were investigated for the radioiodination of boronic acid **1 b**, in which radiochemical yields (RCY) were determined by radio‐HPLC analysis of the crude product.^26^ At a micromolar scale, the gold‐catalysed *ipso*‐substitution reaction was easily modified for radioiodination. [^125^I]NaI (4–6 MBq solution in water) was used as the limiting reagent, and the reaction gave the desired radiolabelled product ^125^I‐**3 b** in 63 % RCY (Table 4, entry 1). Extending the reaction time to 20 min was found to be optimal and gave a quantitative RCY of product ^125^I‐**3 b** (entry 3). The corresponding radio‐HPLC for this transformation showed a particularly clean reaction with no other radiolabelled by‐products (Figure 1). It should be noted that radioiodination can be done without the use of PPh_3_AuNTf_2_. Notably, in parallel with the cold studies, the thermally‐mediated reaction was less efficient and gave only 47 % RCY after 20 min (entry 4).

**Table 4:** Optimisation of gold‐mediated radioiodination of boronic acid **1 b**.^[a]^

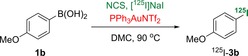			
Entry	*t* [min]	PPh_3_AuNTf_2_ [mol %]	RCY [%]
1	5	50	63
2	15	50	96
3	20	50	100
4	20	0	47

[a] All reactions were performed with a 4–6 MBq solution of [^125^I]NaI in water (0.01 mL). Reactions were performed under thermal heating conditions in a sealed tube.

In radioiodination reactions, a rapid transformation that gives the product cleanly is crucial for generating the target radiolabelled compound in high radiochemical purity, radioactive yield, and molar activity. Therefore, the use of gold catalysis for these radioiodinations rather than the thermally mediated reaction is particularly advantageous because the reactions are faster, more efficient, and easier to purify.

The scope of the optimised gold‐mediated radioiodination reaction was next examined for a range of arylboronic acids (Table 5). Under these conditions, electron‐rich and electron‐deficient arenes with various substitution patterns were found to be suitable substrates for the reaction, and they reacted to give the ^125^I‐labelled products in excellent RCY (92–100 %). Only two arylboronic acids required further optimisation: The initial reactions of ethyl ester and trifluoromethyl analogues **1 h** and **i** under the optimised conditions (90 °C, 20 min) produced radio‐HPLC traces with several radiolabelled by‐products. Repeating the reactions at a lower temperature of 80 °C allowed a cleaner transformation, and despite the need for a slightly longer reaction time (30 min), this gave the products ^125^I‐**3 h** and **i** in excellent RCY. Only sterically hindered boronic acid **1 n** showed no reaction under these conditions, even after 30 min. As observed for the cold iododeborination of this compound, a significantly longer reaction time was likely required for this substrate.

**Table 5:** Substrate scope of gold‐mediated radioiododeboronation.

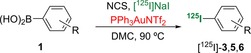
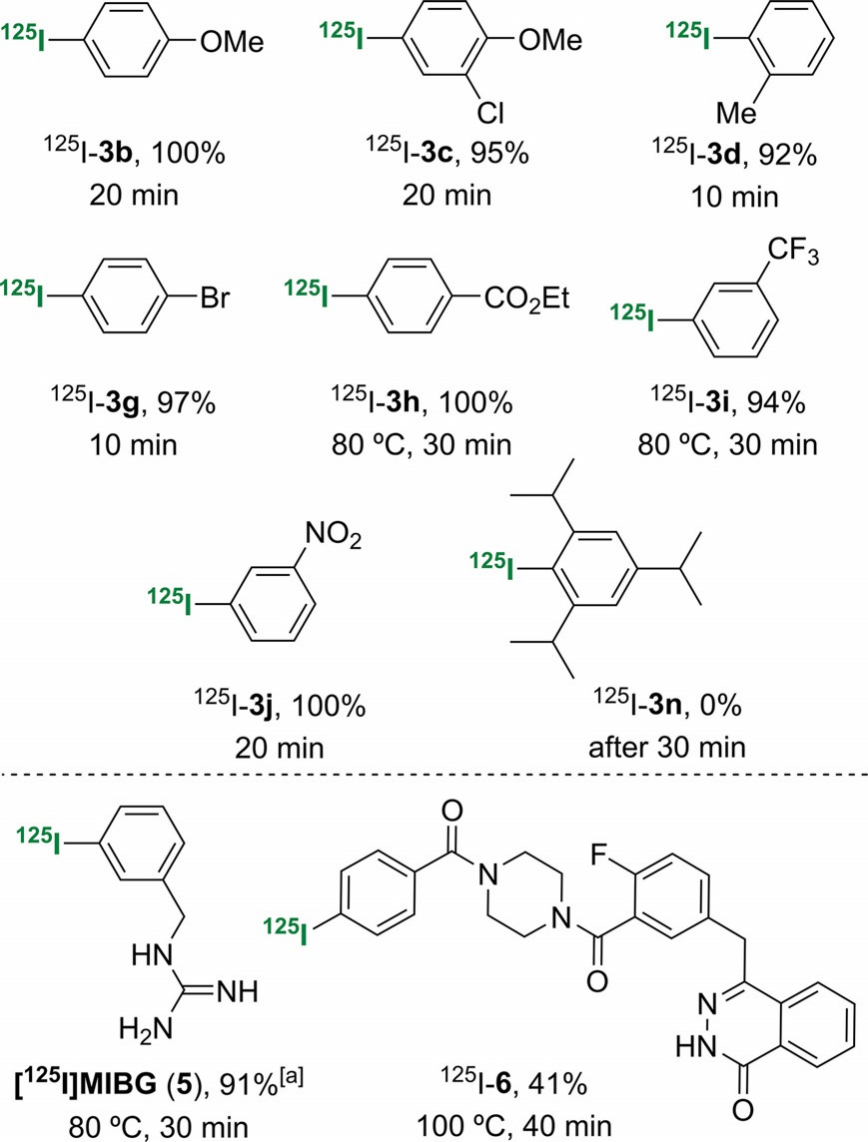

[a] After ^125^I‐iododeborination, a second step that involved HCl‐mediated removal of the Boc‐protecting groups was performed to generate [^125^I]MIBG.

Having shown that this transformation could be used for the radioiododeboronation of simple arylboronic acids, we next investigated the application of this method for radiolabelling biologically active compounds and imaging agents (Table 5). The first target was *meta*‐[^125^I]iodobenzylguanidine (MIBG, **5**). In the ^123^I‐form, MIBG is a commercially available radiopharmaceutical that is used for the SPECT imaging of human norepinephrine transporter‐expressing cancers.^27^ In the ^131^I‐form, MIBG is used for targeted radionuclide therapy.^28^ A di‐Boc‐protected boronic acid analogue of MIBG was efficiently prepared in one step by the coupling of 3‐(aminomethyl)benzeneboronic acid with di‐Boc‐protected 1*H*‐pyrazole‐1‐carboxamidine.^29^ After some optimisation, gold‐mediated *ipso*‐substitution of this arylboronic acid was found to be most effective at 80 °C with a reaction time of 30 min. This produced the corresponding ^125^I‐labelled compound in quantitative RCY. The reaction mixture was then treated with hydrochloric acid to remove the Boc protecting groups, and this gave [^125^I]MIBG (**5**) in 91 % RCY over the two steps. Gold‐mediated radioiododeboronation was also effective for the preparation of phthalazinone **6**, a nanomolar inhibitor and SPECT imaging agent of poly(ADP‐ribose) polymerase‐1 (PARP‐1), which is a diagnostic and therapeutic target for cancer.^30^ Despite the complex structure of this substrate, which contained the amide and N‐heterocycle moieties, gold‐mediated radioiododeborination was achieved by using a reaction temperature of 100 °C and gave ^125^I‐**6** in 41 % RCY.^31^

**Scheme 2:**
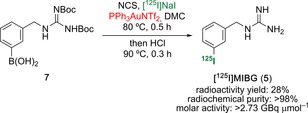
Radiosynthesis of [^125^I]MIBG (**5**).

Following these results, we decided to validate the radioiododeboronation method with the synthesis and purification of [^125^I]MIBG (Scheme 2). Boronic acid **7** was treated with [^125^I]NaI (10.16 MBq) by using our previously optimised gold‐mediated *ipso*‐radioiodination reaction. Following the removal of the Boc protecting groups under acidic conditions and HPLC purification, [^125^I]MIBG (**5**) was isolated in 28 % radioactivity yield. Compound **5** had a radiochemical purity of >98 % and a molar activity of >2.73 GBq μmol^−1^. Identification of the product was confirmed by HPLC, with co‐elution of a sample of unlabelled MIBG. These results compared favourably with other approaches for the preparation of radiolabelled MIBG, which demonstrates the potential of this methodology for widespread use in generating radioiodine‐labelled tracers.9a,9b, 27a, 32

## Conclusions

Fast, air and water stable *ipso*‐iododeboronation reactions in the presence of NIS and the green solvent dimethylcarbonate have been developed. The gold‐catalysed reaction was significantly preferred for electron‐deficient and sterically hindered arylboronic acid substrates, whereas the uncatalysed reaction provided very poor yields. For electron‐rich boronic acids, the gold‐catalysed reaction also provided much higher yields than the uncatalysed procedure. However, heterocyclic and N‐containing arylboronic acids reacted more favourably under uncatalysed conditions. Moreover, the reaction was tolerant of halogen moieties (Br and Cl), which is complementary to the commonly used halogen‐exchange method to form aryl iodides.^33^ The gold‐catalysed iododeboronation reaction was shown to be a highly amenable procedure for the ^125^I‐labelling of arenes, which constitutes the first example of an application of homogenous gold catalysis to selective radiosynthesis. Under the optimised radiochemistry conditions, both electron‐rich and electron‐poor aryl boronic acids were rapidly converted to the radioiodinated products in excellent RCY. This general method was validated with the efficient synthesis and isolation of radioiodinated MIBG, a tracer that is used for the imaging of cancer. Current studies are ongoing to investigate the extension of this methodology for the preparation of existing and novel SPECT imaging agents.

## Experimental Section

### General procedure A: gold(I)‐catalysed reactions

Boronic acid **1** (0.10 mmol, 1.0 equiv), NIS (0.10 mmol, 1.0 equiv), PPh_3_AuNTf_2_ (3.7 mg, 5 mol %), and DMC (0.4 mL) were added to a microwave tube and heated under microwave irradiation at 90 °C for 5 min. The resulting solution was passed through a silica plug and washed with hexanes/diethyl ether (20:1) to yield product **3**. The crude product was purified by column chromatography as needed.

### General procedure B: uncatalysed reaction

Boronic acid **1** (0.10 mmol, 1.0 equiv), NIS (0.10 mmol, 1.0 equiv), and DMC (0.4 mL) were added to a microwave tube and heated under microwave irradiation at 90 °C for 5 min. The resulting solution was passed through a silica plug and washed with hexanes/diethyl ether (20:1) to yield product **3**. The crude product was purified by column chromatography as needed.

### General procedure C: radioiodination

A solution of [^125^I]NaI in water (0.01 mL, 4–6 MBq) was added to a solution of *N*‐chlorosuccinimide (0.50 mg, 3.9 μmol) in DMC (0.1 mL). A solution of 4‐methoxybenzeneboronic acid **1 b** (0.60 mg, 3.9 μmol) and Ph_3_PAuNTf_2_ (3.0 mg, 2.0 μmol) in dimethylcarbonate (0.1 mL) was added, and the reaction mixture heated to 90 °C for 20 min. The reaction mixture was removed by syringe and diluted with acetonitrile/water (1:1, 0.5 mL). Analysis of this solution by analytical radio‐HPLC showed a radiochemical yield of 100 %.

## Conflict of interest

The authors declare no conflict of interest.

## Supporting information

As a service to our authors and readers, this journal provides supporting information supplied by the authors. Such materials are peer reviewed and may be re‐organized for online delivery, but are not copy‐edited or typeset. Technical support issues arising from supporting information (other than missing files) should be addressed to the authors.

Supplementary
