# Supplementary material for: Rapid Iododeboronation with and without Gold Catalysis: Application to Radiolabelling of Arenes
Source: Chemistry. 2017 Dec 14;24(4):937–43. doi: 10.1002/chem.201704534 (PMC5814724; doi:10.1002/chem.201704534)
Supplement: Supplementary file 1 — Supplementary [file CHEM-24-937-s001.pdf]

CHEMISTRY
A European Journal

Supporting Information

Rapid Iododeboronation with and without Gold Catalysis: Application to Radiolabelling of Arenes

Stacey Webster,[a] Kerry M. O'Rourke,[b] Conor Fletcher,[a] Sally L. Pimlott,[c] Andrew Sutherland,*[b] and Ai-Lan Lee*[a]

chem_201704534_sm_miscellaneous_information.pdf

Supporting Information

Contents

A) Development of Cold Procedure

1. **General Experimental** 3
2. **Experimental** 5

B) Development of Hot Procedure

3. **General Experimental** 13
4. **Experimental** 14

C) Spectra and chromatograms

5. **^{1}H and ^{13}C Spectra** 24
6. **Radio-HPLC and UV-Vis HPLC Chromatograms** 42

7. **References** 47

A) Development of Cold Procedure

1. General Experimental

Chemical shifts (δ in ppm) were referenced to tetramethylsilane (TMS) or to residual solvent peaks. *J* values are given in Hz and s, bs, d, dd, ddd, dt, t, td, tt, q, qn, sext and m abbreviations correspond to singlet, broad singlet, doublet, doublet of doublet, doublet of doublet of doublets, doublet of triplets, triplet, triplet of doublets, triplet of triplets quartet, quintet, sextet and multiplet. Mass spectra were obtained at the EPSRC National Mass Spectrometry Service Centre in Swansea. Infrared spectra were obtained deposited neat or as a chloroform solution to a diamond/ZnSe plate. All boronic acids were purchased and used without further purification unless otherwise stated. Dimethylcarbonate (DMC) was purchased and used without further purification. If poor yields were obtained, the dimethyl carbonate was left open to air for 24 - 48 h before use in order to make sure that the solvent is wet enough to aid transmetallation. CEM Microwave Discover was used for microwave heating, using sealed tubes and external surface sensor. The gold(I)-catalysed reactions were carried out without the need for dry solvents or inert atmosphere, unless stated otherwise.

General procedure A: Gold(I)-catalysed reactions

Boronic acid **1** (0.10 mmol, 1.0 equiv.), NIS (0.10 mmol, 1.0 equiv.), PPh_3AuNTf_2 (3.7 mg, 5 mol%) and DMC (0.4 ml) were added to a microwave tube and heated under microwave irradiation at 90 ^{o}C for 5 minutes. The resulting solution was passed through a silica plug and washed with 20:1 hexane/ether to yield product **3**. The crude product was purified by column chromatography as needed.

General procedure B: No catalyst

Boronic acid **1** (0.10 mmol, 1.0 equiv.), NIS (0.10 mmol, 1.0 equiv.) and DMC (0.4 ml) were added to a microwave tube and heated under microwave irradiation at 90 ^{o}C for 5 minutes. The resulting solution was passed through a silica plug and washed with 20:1 hexane/ether to yield product **3**. The crude product was purified by column chromatography as needed.

Thermal vs. Microwave Heating:

It should be noted that although microwave heating is used in our substrate scope studies (Table 3) for practical reasons, we also tested the reactions under thermal heating in a sealed tube. Pleasingly, the reaction works just as efficiently, if not slightly better under thermal heating. Therefore, thermal heating can be used should a microwave reactor not be available.

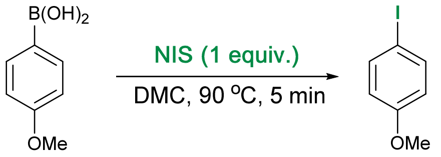

1b **3b**

PPh_3AuNTf_2 (5 mol%)	Thermal heating	90%, >20:1 **3b:4b**
	µwave heating	92%, 18:1 **3b:4b**
No catalyst	Thermal heating	80%, >20:1 **3b:4b**
	µwave heating	73%, >20:1 **3b:4b**

Wet vs. Dry Solvent:

The reaction works more efficiently in wet solvent compared to anhydrous solvent. We have previously described that water aids the transmetallation between PPh_3AuNTf_2 and arylboronic acids,[1] which may explain the better yields in non-anhydrous solvents.

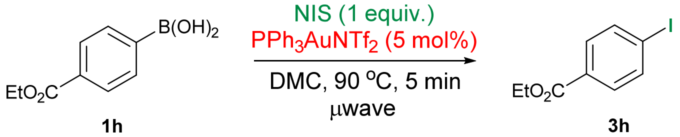

1h **3h**

Anhydrous solvent:	58%
Wet solvent:	78%

.

2. Experimental

1-Iodo-4-methoxybenzene (3b)[2]

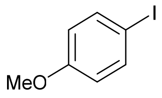

General procedure A followed to yield a mixture of products **3b** and **4b** in a 18:1 ratio as a white solid (92%, 21.6 mg, 0.092 mmol).

General procedure B followed to yield product **3b** as a white solid (73%, 17.4 mg, 0.074 mmol).

Procedure for forming NIS *in situ*

A solution of NaI (15.3 mg, 0.102 mmol, 1.0 equiv) and NCS (13.2 mg, 0.099 mmol, 1.0 equiv.) in DMC (0.4 ml) was stirred at room temperature for 10 mins. Boronic acid **1b** (15.2 mg, 0.10 mmol, 1.0 equiv.) and PPh_3AuNTf_2 (3.7 mg, 5 mol%) was added to the reaction mixture and heated under microwave irradiation for 5 mins at 90 ^oC. The crude mixture was then passed through a silica plug and washed with 20:1 hexane/ether to yield product **3b** as a white solid (80%, 19.4 mg, 0.08 mmol).

This procedure can also be carried out without the addition of PPh_3AuNTf_2 to yield product **3b** as a white solid (57%, 13.9 mg, 0.057 mmol).

Procedure for thermal heating

Boronic acid **1b** (15.3 mg, 0.101 mmol, 1.0 equiv.), NIS (22.6 mg, 0.100 mmol, 1.0 equiv.), PPh_3AuNTf_2 (3.8 mg, 5 mol%) and DMC (0.4 ml) were added to a sealed tube and heated in a silicon oil bath at 90 ^oC for 5 mins. The crude mixture was then passed through a silica plug and washed with 20:1 hexane/ether to yield product **3b** as a white solid (90%, 22.0 mg, 0.090 mmol).

This procedure can also be carried out without the addition of PPh_3AuNTf_2 to yield product **3b** as a white solid (73%, 19.2 mg, 0.073 mmol).

Mp: 56-58 ^oC ($CDCl_3$) [Lit.[3] mp 53-55 ^oC]; v_{max} (cm^{-1}) 3065, 2962, 2937 (C-H), 1484, 1455, 1439 (Ar C-C), 1242 (C-O); ^1H NMR (300 MHz, $CDCl_3$) δ 7.56 (2H, d, J = 6.8 HZ, Ar-H), 6.68 (2H, d, J = 6.8 Hz, Ar-H), 3.78 (3H, s, OCH_3); ^{13}C NMR (75.5 MHz, $CDCl_3$) δ 159.6 (C), 138.3 (CH), 116.5 (CH), 82.8 (C), 55.5 (CH_3).

4-Iodo-1,2-diemethoxybenzene (3a)[4]

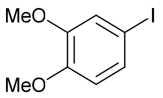

General procedure A followed but the reaction was carried out at 100 °C instead of 90 °C, to yield a mixture of products **3a** and **4a** in a 12:1 ratio as a yellow oil (85%, 22.5 mg, 0.085 mmol).

General procedure B followed but the reaction was carried out at 100 °C instead of 90 °C, to yield product **3a** as a yellow oil (70%, 18.4 mg, 0.070 mmol).

v_{max} (cm^{-1}) 2944, 2947, 2848 (C-H), 1447, 1453, 1435 (Ar C-C), 1196 (C-O); ^{1}H NMR (300 MHz, $CDCl_3$) δ 7.22 (1H, dd, *J* = 8.4, 2.0 Hz, Ar-H), 7.12 (1H, d, *J* = 2.0 Hz, Ar-H), 6.62 (1H, d, *J* = 8.4 Hz, Ar-H), 3.85 (3H, s, OCH_3), 3.84 (3H, s, OCH_3); ^{13}C NMR (75.5 MHz, $CDCl_3$) δ 149.9 (C), 149.2 (C), 129.9 (CH), 120.5 (CH), 113.3 (CH), 82.4 (C), 56.2 (CH_3), 56.1 (CH_3).

2-Chloro-4-iodo-1-methoxybenzene (3c)[5]

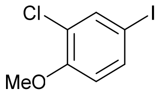

General procedure A followed, to yield a mixture of products **3c** as a white solid (100%, 26.6 mg, 0.10 mmol).

General procedure B followed to yield product **3c** as a white solid (77%, 20.6 mg, 0.077 mmol).

Mp: 96-97 °C ($CDCl_3$) [Lit.[6] mp 93-95 °C]; v_{max} (cm^{-1}) 3066, 2934, 2837 (C-H), 1476, 1460, 1437 (Ar C-C), 1250 (C-O); ^{1}H NMR (300 MHz, $CDCl_3$) δ 7.65 (1H, d, *J* = 2.2 Hz, Ar-H), 7.51 (1H, dd, *J* = 8.7, 2.2 Hz, Ar-H), 6.68 (1H, d, *J* = 8.7 Hz, Ar-H), 3.88 (3H, s, OCH_3); ^{13}C NMR (75.5 MHz, $CDCl_3$) δ 155.2 (C), 138.3 (CH), 136.7 (CH), 123.9 (C), 114.1 (CH), 82.0 (C), 56.3 (CH_3).

1-Iodo-2-methylbenzene (3d)[2]

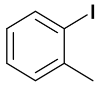

General procedure A followed but the reaction was carried out at 100 ^oC instead of 90 ^oC, to yield product **3d** as a yellow oil (100%, 21.5 mg, 0.100 mmol).

General procedure B followed but the reaction was carried out at 100 ^oC instead of 90 ^oC, to yield product **3d** and an unidentified side product as a yellow oil (62%, 13.2 mg, 0.062 mmol).

v_{max} (cm^{-1}) 3055, 2919 (C-H), 1473, 1462, 1453 (Ar C-C); ^1H NMR (400 MHz, $CDCl_3$) δ 7.81 (1H, d, *J* = 7.6 Hz, Ar-H), 7.23-7.25 (2H, m, Ar-H), 6.84-6.96 (1H, m, Ar-H) 2.44 (3H, s, CH_3); ^{13}C NMR (75.5 MHz, $CDCl_3$) δ 141.5 (C), 139.1 (CH), 129.9 (CH), 128.3 (CH), 127.5 (CH), 101.3 (C), 28.3 (CH_3).

2-(4-Iodophenoxy)tetrahydro-2*H*-pyran (3f)[7]

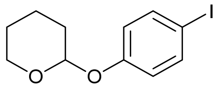

General procedure A followed to yield a complex mixture of products.

General procedure B followed but 4 Å molecular sieves (1 bead) was added to reaction mixture (to neutralise any trace acids). The crude was passed through an alumina plug to yield product **3f** as a yellow oil (60%, 17.6 mg, 0.060 mmol).

v_{max} (cm^{-1}) 2943, 2873 (C-H), 1453, 1440, 1389 (Ar C-C), 1235 (C-O); ^1H NMR (300 MHz, $CDCl_3$) δ 7.55 (2H, d, *J* = 8.9 Hz, Ar-H), 6.83 (2H, d, *J* = 8.9 Hz, Ar-H), 5.38 (1H, t, *J* = 3.2 Hz, OCHO), 3.81-3.90 (1H, m, $OC\underline{H}_2CH_2$), 3.59 (1H, m, $OC\underline{H}_2CH_2$), 1.89-2.07 (1H, m, alkyl-H), 1.81-1.89 (2H, m, alkyl-H), 1.54-1.75 (3H, m, alkyl-H); ^{13}C NMR (75.5 MHz, $CDCl_3$) δ 157.1 (C), 138.3 (CH), 119.0 (CH), 96.5 (CH), 84.1 (C), 62.1 (CH_2), 30.4 (CH_2), 25.3 (CH_2), 18.8 (CH_2).

1-Bromo-4-iodobenzene (3g)[8]

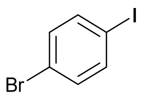

General procedure A followed to yield product **3g** as a white solid (71%, 20.0 mg, 0.071 mmol).

General procedure B followed to yield product **3g** as a white solid (33%, 9.2 mg, 0.033 mmol).

Mp: 94-96 °C ($CDCl_3$) [Lit.[9] mp 91-92 °C (ethanol)]; ν_{max} (cm^{-1}) 3071, 2923 (C-H), 1484, 1467, 1447 (Ar C-C); ^1H NMR (300 MHz, $CDCl_3$) δ 7.54 (2H, d, *J* = 8.6 Hz, Ar-H), 7.23 (2H, d, *J* = 8.6 Hz, Ar-H); ^{13}C NMR (75.5 MHz, $CDCl_3$) δ 139.2 (CH), 133.6 (CH), 122.3 (C), 92.2 (C).

Ethyl 4-iodobenzoate (3h)[4]

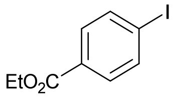

General procedure A followed to yield product **3h** as a colourless oil (78%, 21.4 mg, 0.078 mmol).

General procedure B followed to yield product **3h** as a colourless oil (25%, 7.0 mg, 0.025 mmol).

ν_{max} (cm^{-1}) 3069, 2976, 2934 (C-H), 1715 (C=O), 1476, 1463, 1444 (Ar C-C), 1266 (C-O); ^1H NMR (400 MHz, $CDCl_3$) δ 7.80 (2H, d, *J* = 8.8 Hz, Ar-H), 7.74 (2H, d, *J* = 8.8 Hz, Ar-H), 4.36 (2H, q, *J* = 7.1 Hz, $OC\underline{H}_2CH_3$), 1.39 (3H, t, *J* = 7.1 Hz, $OCH_2C\underline{H}_3$); ^{13}C NMR (75.5 MHz, $CDCl_3$) δ 166.2 (C), 137.9 (CH), 131.1 (CH), 130.2 (C), 100.7 (C), 61.4 (CH_2), 14.4 (CH_3).

1-Iodo-3-(trifluoromethyl)benzene (3i)[10]

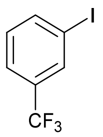

General procedure A followed to yield product **3i** as a brown oil (62%, 16.7 mg, 0.062 mmol).

General procedure B followed to yield product **3i** as a brown oil (13%, 3.4 mg, 0.013 mmol).

ν_{max} (cm^{-1}) 3070, 2975 (C-H), 1474, 1421 (Ar C-C); ^{1}H NMR (300 MHz, $CDCl_3$) δ 7.96 (1H, br s, Ar-H), 7.90 (1H, d, *J* = 7.9 Hz, Ar-H), 7.60 (1H, d, *J* = 8.6 Hz, Ar-H), 7.22 (1H, t, *J* = 7.9 Hz, Ar-H); ^{13}C NMR (75.5 MHz, $CDCl_3$) δ 141.0 (CH, q, *J* = 1.1 Hz), 134.4 (CH, q, *J* = 3.9 Hz), 132.8 (C, t, *J* = 32.8 Hz), 130.5 (CH), 124.6 (CH, q, *J* = 14.1 Hz), 121.3 (C, *J* = 273.2 Hz), 94.0 (C).

1-Iodo-3-nitrobenzene (3j)[11]

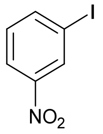

General procedure A followed but reaction time increased to 10 mins to yield product **3j** as a yellow oil (78%, 19.2 mg, 0.077 mmol).

General procedure B followed but reaction time increased to 60 mins using 1.1 equiv. of NIS to yield product **3j** as a yellow oil (16%, 4.0 mg, 0.016 mmol).

ν_{max} (cm^{-1}) 3088, 2858 (C-H), 1518, 1340 (NO_2), 1460, 1418 (Ar C-C); ^{1}H NMR (300 MHz, $CDCl_3$) δ 8.57 (1H, t, *J* = 1.9 Hz, Ar-H), 8.21 (1H, ddd, *J* = 7.9, 1.9, 1.0 Hz), 8.03 (1H, ddd, *J* = 7.9, 1.9, 1.0 Hz), 7.26 (1H, t, *J* = 7.9 Hz); ^{13}C NMR (75.5 MHz, $CDCl_3$) δ 148.7 (C), 143.6 (CH), 132.6 (CH), 130.8 (CH), 122.9 (CH), 93.6 (C).

1-(4-iodophenyl)ethan-1-one (3l)[2]

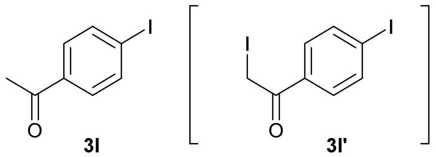

3l [**3l'**]

General procedure A followed to yield a 1:1 ratio of **3l** and **3l'** (45%, 10.9 mg, 0.045 mmol) as an inseparable mix.

General procedure B followed to yield product **3l** as white solid (35%, 8.6 mg, 0.035 mmol).

Mp: 86-87 ^{o}C ($CDCl_3$) [Lit.[12] mp 86-87 ^{o}C (benzene)]; ν_{max} (cm^{-1}) 2972 (C-H), 1667 (C=O) 1471, 1389 (Ar C-C); ^{1}H NMR (300 MHz, $CDCl_3$) δ 7.83 (2H, d, *J* = 8.6 Hz, Ar-H), 7.66 (2H, d, *J* = 8.6 Hz, Ar-H), 2.57 (3H, s, CH_3); ^{13}C NMR (75.5 MHz, $CDCl_3$) δ 197.5 (C), 138.0 (CH), 136.5 (C), 129.9 (CH), 101.2 (C), 26.6 (CH_3).

4-Iodo-1,3,4-triisopropylbenzene (3n)[13]

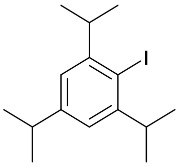

With gold catalyst: Boronic acid **1n** (24.7 mg, 0.100 mmol, 1.0 equiv.), NIS (24.9 mg, 0.111 mmol, 1.1 equiv.), PPh_3AuNTf_2 (3.6 mg, 5 mol%) and DMC (0.4 ml) were added to the microwave tube and heated under microwave irradiation for 3 h at 90 ^{o}C. The crude mixture was passed through a silica plug and washed with hexane to yield product **3n** as a colourless oil (78%, 25.6 mg, 0.078 mmol).

Without gold catalyst: Boronic acid **1n** (24.7 mg, 0.100 mmol, 1.0 equiv.), NIS (24.9 mg, 0.111 mmol, 1.1 equiv.) and DMC (0.4 ml) were added to the microwave tube and heated under microwave irradiation for 3 h at 90 ^{o}C. The crude mixture was passed through a silica plug and washed with hexane to yield product **3n** as a colourless oil (14%, 4.6 mg, 0.014 mmol).

ν_{max} (cm^{-1}) 2958, 2926, 2868 (C-H), 1460, 1420, 1382 (Ar C-C); ^{1}H NMR (300 MHz, $CDCl_3$) δ 6.97 (2H, s, Ar-H), 3.41 (2H, sept, *J* = 6.9 Hz, $(CH_3)_2C\underline{H}CCI$), 2.89 (1H, sept,

J = 6.9 Hz, $(CH_3)_2C\underline{H}C$), 1.27 (6H, d, *J* = 6.9 Hz, $(C\underline{H}_3)_2CHC$), 1.25 (12H, d, *J* = 6.9 Hz, $(C\underline{H}_3)_2CHCCl$); ^{13}C NMR (75.5 MHz, $CDCl_3$) δ 150.9 (C), 149.0 (C), 122.2 (CH), 105.9 (C), 39.5 (CH), 34.0 (CH), 24.1 (CH_3), 23.6 (CH_3).

6-Chloro-3-iodo-2-methoxypyridine (3o)[14]

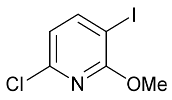

General procedure A followed to yield a complex mixture of products.

General procedure B followed to yield product **3o** as a white solid (51%, 13.6 mg, 0.050 mmol).

Mp: 65-66 ^{o}C ($CDCl_3$); ν_{max} (cm^{-1}) 2951 (C-H), 1462, 1409, 1365 (Ar C-C), 1141 (C-O); ^{1}H NMR (300 MHz, $CDCl_3$) δ 7.93 (1H, d, *J* = 7.8 Hz, Ar-H), 6.70 (1H, d, *J* = 7.8 Hz, Ar-H), 3.99 (3H, s, OCH_3); ^{13}C NMR (75.5 MHz, $CDCl_3$) δ 161.9 (C), 149.8 (CH), 148.8 (C), 118.2 (CH), 76.8 (C), 55.5 (CH_3).

2-Iodobenzo[*b*]thiophene (3p)[11]

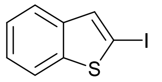

General procedure A followed to yield a complex mixture of products.

General procedure B followed to yield product **3p** as a white solid (78%, 20.0 mg, 0.077 mmol).

Mp: 68-69 ^{o}C ($CDCl_3$) [Lit.[15] mp 63-65 ^{o}C (hexane)]; ν_{max} (cm^{-1}) 3053 (C-H), 1494, 1454, 1420 (Ar C-C), 1141 (C-O); ^{1}H NMR (300 MHz, $CDCl_3$) δ 7.74-7.89 (1H, m, Ar-H), 7.68-7.73 (1H, m, Ar-H), 7.52-7.55 (1H, m, Ar-H), 7.27-7.31 (2H, m, Ar-H); ^{13}C NMR (75.5 MHz, $CDCl_3$) δ 144.5 (C), 140.9 (C), 133.9 (CH), 124.6 (CH), 124.5 (CH), 122.4 (CH), 121.4 (CH), 78.5 (C).

4-Iodo-*N,N*-diphenylaniline (3q)[16]

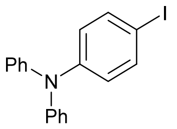

General procedure A followed to yield a complex mixture of products.

General procedure B followed to yield product **3q** as a yellow oil (100%, 24.9 mg, 0.100 mmol).

ν_{max} (cm^{-1}) 3034 (C-H), 1481, 1311 (Ar C-C); ^1H NMR (300 MHz, $CDCl_3$) δ 7.41 (2H, d, *J* = 8.9 Hz, Ar-H), 7.13-7.21 (4H, m, Ar-H), 6.92-7.02 (6H, m, Ar-H), 6.74 (2H, d, *J* = 8.9 Hz, Ar-H); ^{13}C NMR (75.5 MHz, $CDCl_3$) δ 147.9 (C), 147.4 (C), 138.2 (CH), 129.5 (CH), 125.4 (CH), 124.7 (CH), 123.5 (CH), 84.9 (C).

9-(4-Iodophenyl)-9*H*-carbazole (3r)[17]

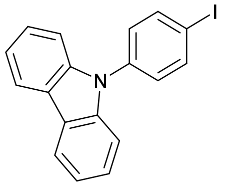

General procedure A followed to yield a complex mixture of products.

General procedure B followed to yield product **3r** as an orange oil (32%, 11.8 mg, 0.032 mmol).

ν_{max} (cm^{-1}) 3058 (C-H), 1450, 1483, 1493 (Ar C-C); ^1H NMR (300 MHz, $CDCl_3$) δ 8.14 (2H, dt, *J* = 7.7, 1.1 Hz, Ar-H), 7.93 (2H, d, *J* = 8.7 Hz, Ar-H), 7.26-7.45 (8H, m, Ar-H); ^{13}C NMR (75.5 MHz, $CDCl_3$) δ 140.5 (C), 139.2 (CH), 137.7 (C), 129.1 (CH), 126.2 (CH), 123.7 (C), 120.5 (CH), 120.4 (CH), 109.7 (CH), 92.2 (C).

B) Development of Hot Procedure

3. General Experimental

All reagents and starting materials were obtained from commercial sources and used as received unless otherwise stated. Dry solvents (dichloromethane) were purified using a PureSolv 500 MD solvent purification system. All reactions were performed open to air unless otherwise stated. Brine is defined as a saturated aqueous solution of sodium chloride. Room temperature refers to 20–25 °C. Flash column chromatography was carried out using Fisher Matrix silica 60. Macherey-Nagel aluminium-backed plates, pre-coated with silica gel 60 (UV254) were used for thin layer chromatography and were visualised using UV light (254/365 nm) then potassium permanganate solution. ^{1}H and ^{13}C NMR spectra were recorded on a Bruker DPX 400 (^{1}H: 400 MHz; ^{13}C: 101 MHz) spectrometer or a Bruker 500 (^{1}H: 500 MHz; ^{13}C: 126 MHz) spectrometer with chemical shift values reported in ppm relative to a residual solvent peak and in the solvent stated. Assignment of ^{1}H NMR signals is based on COSY experiments. Assignment of ^{13}C NMR signals is based on HSQC and/or DEPT experiments. All coupling constants, *J*, are quoted in Hz. Infrared spectra were recorded using Golden Gate apparatus on a JASCO FTIR 410 spectrometer directly as either a solid or liquid. Mass spectra were obtained using a JEOL JMS-700 spectrometer. Melting points were determined on a Gallenkamp melting point apparatus. 4-[4''-Fluoro-3''-(piperazine-1'''-carbonyl)benzyl]-2*H*-phthalazin-1-one was prepared as previously reported.[18] *N*-(3-Iodobenzyl)guanidine was purchased from Sigma-Aldrich and used as the HPLC standard for the development of the radiosynthesis of *N*-(3-[^{125}I]iodobenzyl)guanidine.

4. Experimental

{3-[2,3-Bis(*tert*-butoxycarbonyl)guanidino]methylphenyl}boronic acid (7)[19]

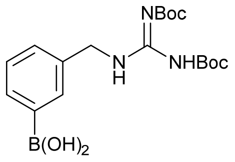

N,N'-Bis(*tert*-butoxycarbonyl)-1*H*-pyrazole-1-carboxamidine (50 mg, 0.16 mmol) and 3-(aminomethyl)benzeneboronic acid hydrochloride (33 mg, 0.18 mmol) were dissolved in methanol (5 mL). Triethylamine (0.10 mL, 0.48 mmol) was added and the mixture stirred for 12 h at room temperature. The solvent was removed *in vacuo* and the crude material was purified by flash column chromatography, eluting with 50% ethyl acetate in petroleum ether to give {3-[2,3-bis(*tert*-butoxycarbonyl)guanidino]methylphenyl}boronic acid (**7**) as a yellow solid (47 mg, 75%). Spectroscopic data were consistent with the literature.[19]

Mp 111–113 °C; δ_H (400 MHz, CD_3OD) 1.47 (9H, s, O*t*Bu), 1.50 (9H, s, O*t*Bu), 4.55 (2H, s, CH_2), 7.29–7.39 (2H, m, 2 × ArH), 7.53–7.75 (2H, m, 2 × ArH); δ_C (101 MHz, CD_3OD) 26.9 (3 × CH_3), 27.2 (3 × CH_3), 44.2 (CH_2), 79.1 (C), 83.2 (C), 127.6 (CH), 128.8 (d, $J_{C\text{-}C\text{-}C\text{-}B}$ 50.2 Hz, CH), 132.6 (d, $J_{C\text{-}C\text{-}B}$ 39.3 Hz, 2 × CH), 136.5 (C), 152.8 (C), 156.1 (C), 163.2 (C) (signal for carbon directly attached to boron is not observed); *m/z* (ESI) 394 (MH^+. 20%).

4-{3'-[4''-(Benzoyl-4'''-boronic acid)piperazine-1''-carbonyl]-4'-fluorobenzyl}-2*H*-phthalazin-1-one

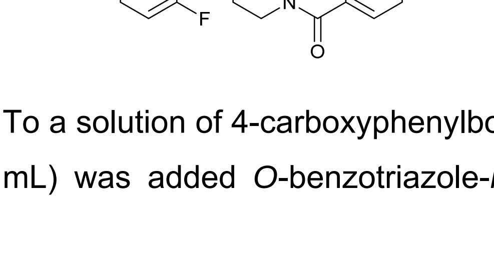

To a solution of 4-carboxyphenylboronic acid (50.0 mg, 0.300 mmol) in acetonitrile (6 mL) was added *O*-benzotriazole-*N,N,N',N'*-tetramethyluroniumhexafluorophosphate

(125 mg, 0.330 mmol) and *N*,*N'*-diisopropylethylamine (100 µL, 0.450 mmol) and the solution was stirred for 1 h at room temperature. 4-[4''-Fluoro-3''-(piperazine-1'''-carbonyl)benzyl]-2*H*-phthalazin-1-one (110 mg, 0.30 mmol) was then added and the mixture was stirred at room temperature for 48 h. The mixture was diluted with water (10 mL), saturated aqueous sodium bicarbonate (10 mL) and extracted with ethyl acetate (3 × 10 mL). The organic layers were combined and washed with brine (30 mL). The organic layer was dried ($MgSO_4$), filtered and concentrated *in vacuo* to give the crude product. Purification was carried out by flash column chromatography, eluting with 80% ethyl acetate in petroleum ether to give 4-{3'-[4''-(benzoyl-4'''-boronic acid)piperazine-1''-carbonyl]-4'-fluorobenzyl}-2*H*-phthalazin-1-one as a white solid (50.0 mg, 33%).

Mp 166–168 °C; ν_{max} (neat)/cm^{-1} 3017 (NH), 2970 (CH), 1740 (CO), 1729 (CO), 1366, 1229, 1217; δ_H (400 MHz, CD_3OD) 3.46–3.82 (8H, m, 4 × NCH_2), 4.37 (2H, s, 7'-H_2), 6.84 (2H, d, *J* 7.5 Hz, 3'''-H and 5'''-H), 7.15 (1H, t, *J* 8.8 Hz, 5'-H), 7.31 (2H, d, *J* 7.5 Hz, 2'''-H and 6'''-H), 7.36 (1H, d, *J* 6.1 Hz, 2'-H), 7.45–7.50 (1H, m, 4'-H), 7.80 (1H, t, *J* 7.8 Hz, 7-H), 7.85 (1H, t, *J* 7.8 Hz, 6-H), 7.93 (1H, d, *J* 7.8 Hz, 5-H), 8.35 (1H, d, *J* 7.8 Hz, 8-H); δ_C (101 MHz, CD_3OD) 29.4 (2 × CH_2), 36.9 (CH_2), 41.6 (2 × CH_2), 114.9 (2 × CH), 115.8 (CH, d, $J_{C\text{-}C\text{-}F}$ 21.8 Hz), 123.2 (C, d, $J_{C\text{-}C\text{-}F}$ 17.1 Hz), 125.3 (C), 125.4 (CH), 126.1 (CH), 127.8 (C), 128.7 (CH, d, $J_{C\text{-}C\text{-}C\text{-}F}$ 3.8 Hz), 129.1 (2 × CH), 129.5 (C), 131.4 (CH), 132.0 (CH, d, $J_{C\text{-}C\text{-}C\text{-}F}$ 8.0 Hz), 133.6 (CH), 135.0 (C, d, $J_{C\text{-}C\text{-}C\text{-}C\text{-}F}$ 3.8 Hz), 146.2 (C), 157.0 (C, d, $J_{C\text{-}F}$ 247.2 Hz), 159.6 (C), 160.9 (C), 165.9 (C), 171.8 (C); *m/z* (ESI) 537.1718 (MNa^+. $C_{27}H_{24}BFN_4NaO_5$ requires 537.1716).

4-[3'-[4''-(4'''-Iodobenzoyl)piperazine-1''-carbonyl]-4'-fluorobenzyl]-2*H*-phthalazin-1-one (6)

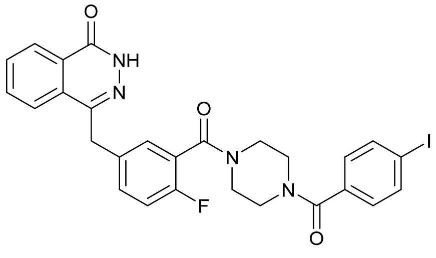

To a solution of 4-iodobenzoic acid (42.0 mg, 0.170 mmol) in *N*,*N*'-dimethylformamide (7 mL) was added triethylamine (37.0 µL, 0.272 mmol), followed by *O*-benzotriazole-

N,N,N',N'-tetramethyluroniumhexafluorophosphate (71.0 mg, 0.187 mmol) and the mixture was stirred at room temperature for 2 h. 4-[4''-Fluoro-3''-(piperazine-1'''-carbonyl)benzyl]-2*H*-phthalazin-1-one (60.0 mg, 0.170 mmol) was added and the mixture was stirred for a further 48 h. Water (14 mL) was then added, followed by 1 h of stirring after which the mixture was cooled to 0 °C. The resulting precipitate was collected by vacuum filtration and washed with diethyl ether (4 × 10 mL) and hexane (4 × 10 mL). Purification by flash column chromatography, using 4% methanol in dichloromethane gave 4-[3'-[4''-(4'''-iodobenzoyl)piperazine-1''-carbonyl]-4'-fluorobenzyl]-2*H*-phthalazin-1-one (**6**) (34.8 mg, 34%) as a white foam.

ν_{max}/cm^{-1} (neat) 3198 (NH), 2899 (CH), 1628 (CO), 1587 (C=C), 1427, 1254, 1225, 1001, 747; δ_H (400 MHz, $CDCl_3$) 3.14–4.02 (8H, m, 4 × NCH_2), 4.28 (2H, s, 7'-H_2), 7.04 (1H, t, *J* 7.8 Hz, 5'-H), 7.14 (2H, d, *J* 8.0 Hz, 3'''-H and 5'''-H), 7.29–7.37 (2H, m, 2'-H and 6'-H), 7.67–7.84 (5H, m, ArH), 8.42–8.51 (1H, m, 8-H), 10.96 (1H, br s, NH); δ_C (101 MHz, $CDCl_3$) 37.7 (CH_2), 42.1 (2 × CH_2), 47.1 (2 × CH_2), 96.6 (C), 116.2 (d, $J_{C\text{-}C\text{-}F}$ 21.7 Hz, CH), 123.6 (d, $J_{C\text{-}C\text{-}F}$ 17.7 Hz, C), 125.0 (CH), 127.2 (CH), 128.4 (C), 128.9 (2 × CH), 129.3 (d, $J_{C\text{-}C\text{-}C\text{-}F}$ 3.6 Hz, CH), 129.5 (C), 131.7 (CH), 131.9 (d, $J_{C\text{-}C\text{-}C\text{-}F}$ 8.0 Hz, CH), 133.7 (CH), 134.4 (C), 134.6 (d, $J_{C\text{-}C\text{-}C\text{-}C\text{-}F}$ 3.7 Hz, C), 137.9 (2 × CH), 145.5 (C), 157.1 (d, $J_{C\text{-}F}$ 247.1 Hz, C), 160.4 (C), 165.2 (C), 169.7 (C); *m/z* (ESI) 619.0597 (MNa^+. $C_{27}H_{22}FIN_4NaO_3$ requires 619.0613).

Radiochemistry Methodology

General Experimental for Radioiodination of Aryl Boronic Acids with [^{125}I]NaI

Reductant free [^{125}I]NaI was purchased from Perkin Elmer (product number NEZ033H005MC) with a specific radioactivity of 643.8 GBq/mg in 0.1 M NaOH (pH 12–14) aqueous solution. The concentration of batch one was 13.85 GBq/mL and the concentration of batch two was 12.75 GBq/mL. All radiochemical yields were determined by radio-HPLC analysis of the crude product.

Analytical Radio-HPLC Method for Determination of Radioiodide Incorporation

Analytical HPLC was performed with a Dionex Ultimate 3000 HPLC system equipped with a Flowstar LB 513 NaI scintillation detector and a DAD-3000 UV detector using a Synergi 4 µm Hydro-RP 80 Å column (150 × 4.6 mm) with 10 mm guard cartridge, UV 254 nm and flow 1 mL/min. The mobile phase for the analysis of substrates was water:acetonitrile. Analysis of the reaction mixture (to assess radioiodide incorporation) used a gradient profile of water and acetonitrile, as shown below.

Time (mins)	%MeCN
0–20	10–95
20–24	95
24–25	95–10
25–30	10

Co-elution with the UV signal from the ^{127}I-compound was used to confirm identity of the ^{125}I-product from each reaction described.

General Method for Radioiodination: 4-[^{125}I]Iodoanisole (^{125}I-3b)

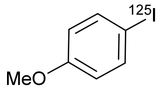

To a solution of *N*-chlorosuccinimide (0.50 mg, 3.9 µmol) in dimethylcarbonate (0.1 mL) was added a 4–6 MBq solution of [^{125}I]NaI in water (0.01 mL). A solution of 4-methoxybenzeneboronic acid (0.60 mg, 3.9 µmol) and $Ph_3PAuNTf_2$ (3.0 mg, 2.0 µmol) in dimethylcarbonate (0.1 mL) was added and the reaction mixture heated to 90 °C for 0.3 h. The reaction mixture was then removed by syringe and diluted with a 1:1 mixture of acetonitrile and water (0.5 mL). Analysis of this solution by analytical radio-HPLC showed a radiochemical yield of 100%.

2-Chloro-4-[^{125}I]iodoanisole (^{125}I-3c)

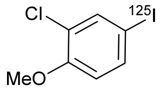

The reaction was done using 3-chloro-4-methoxybenzeneboronic acid (0.60 mg, 3.2 µmol) as described in the general procedure. Analysis by radio-HPLC gave a radiochemical yield of 95%.

2-Methyl-[^{125}I]iodobenzene (^{125}I-3d)

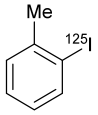

The reaction was done using 2-methylbenzeneboronic acid (0.60 mg, 4.4 µmol) as described in the general procedure, except that the reaction was complete in 0.15 h. Analysis by radio-HPLC gave a radiochemical yield of 92%.

4-Bromo-[^{125}I]iodobenzene (^{125}I-3g)

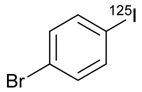

The reaction was done using 4-bromobenzeneboronic acid (0.60 mg, 3.0 µmol) as described in the general method, except that the reaction was complete in 0.15 h. Analysis by radio-HPLC gave a radiochemical yield of 97%.

Ethyl 4-[^{125}I]iodobenzoate (^{125}I-3h)

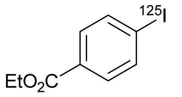

The reaction was done using 4-ethoxycarbonylbenzeneboronic acid (0.60 mg, 3.1 µmol) as described in the general procedure, except that the reaction was heated to 80 °C for 0.5 h. Analysis by radio-HPLC gave a radiochemical yield of 100%.

3-Trifluoromethyl-[^{125}I]iodobenzene (^{125}I-3i)

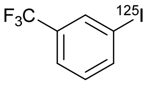

The reaction was done using 3-(trifluoromethyl)benzeneboronic acid (0.60 mg, 3.2 µmol) as described in the general procedure, except that the reaction was heated to 80 °C for 0.5 h. Analysis by radio-HPLC gave a radiochemical yield of 94%.

3-Nitro-[^{125}I]iodobenzene (^{125}I-3j)

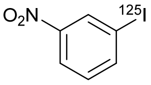

The reaction was done using 3-nitrobenzeneboronic acid (0.6 mg, 3.6 µmol) as described in the general procedure. Analysis by radio-HPLC gave a radiochemical yield of 100%.

N-(3-[125I]Iodobenzyl)guanidine ([125I]MIBG, 5)

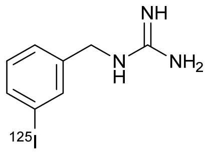

The radio-iododeboronation reaction was done using {3-[2,3-bis(*tert*-butoxycarbonyl)guanidino]methylphenyl}boronic acid (0.60 mg, 1.5 μmol) as described in the general procedure, except that $Ph_3PAuNTf_2$ (2.3 mg, 1.5 μmol) was used and the reaction was heated to 80 °C for 0.5 h. Analysis by radio-HPLC gave a radiochemical yield of 100% for *N*',*N*''-bis(*tert*-butoxycarbonyl)-*N*-3-[^{125}I]iodobenzylguanidine. To the reaction mixture was added 6 M hydrochloric acid (0.2 mL) and the reaction mixture stirred at 90 °C for 0.3 h. Analysis by radio-HPLC gave a radiochemical yield of 91% for *N*-(3-[^{125}I]iodobenzyl)guanidine.

4-{3′-[4″-(4‴-[^{125}I]Iodobenzoyl)piperazine-1″-carbonyl]-4′-fluorobenzyl}-2H-phthalazin-1-one (^{125}I-6)

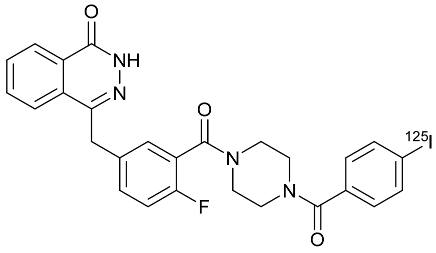

The reaction was done using 4-{3'-[4''-(benzoyl-4'''-boronic acid)piperazine-1''-carbonyl]-4'-fluorobenzyl}-2*H*-phthalazin-1-one (0.60 mg, 1.2 μmol) as described in the general procedure, except that $Ph_3PAuNTf_2$ (1.9 mg, 1.2 μmol) was used and the reaction was heated to 100 °C for 0.6 h. Analysis by radio-HPLC gave a radiochemical yield of 41%.

The use of arylBpin instead of arylboronic acid resulted in a lower yield:

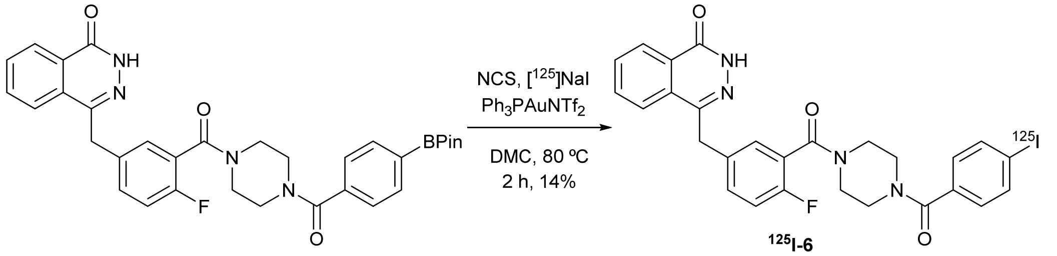

125I-6

Semi-Preparative Radio-HPLC Method for Purification of *N*-(3-[^{125}I]iodobenzyl)guanidine

Semi-preparative HPLC was performed with a Dionex Ultimate 3000 HPLC system equipped with a Knauer Advanced Scientific Instruments Smartline UV Detector 2500 and a photomultiplier tube (PMT) connected to a Lab Logic Flow-Count radiodetector and using a Synergi 4 µm Hydro-RP 80 Å column (150 × 10 mm) with 10 mm guard cartridge, UV 254 nm and flow 3 mL/min. A gradient profile of water and acetonitrile was used, as shown below.

Time (mins)	%MeCN
0–20	10–95
20–24	95
24–25	95–10
25–30	10

Synthesis and Purification of *N*-(3-[^{125}I]iodobenzyl)guanidine ([^{125}I]MIBG, 5)

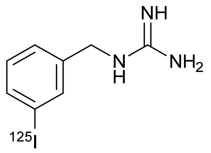

To a solution of *N*-chlorosuccinimide (0.5 mg, 3.9 μmol, 1.0) in dimethylcarbonate (0.1 mL) was added a 10.16 MBq solution of [^{125}I]NaI in water (0.01 mL). A solution of {3-[2,3-bis(*tert*-butoxycarbonyl)guanidino]methylphenyl}boronic acid (0.6 mg, 1.5 μmol) and $Ph_3PAuNTf_2$ (2 mg, 1.5 μmol) in dimethylcarbonate (0.1 mL) was added and the reaction mixture heated to 80 °C for 0.5 h. 6 M Hydrochloric acid (0.2 mL) was added and the reaction was heated to 90 °C for 0.3 h. The mixture was diluted in a 1:1 mixture of acetonitrile and water (0.5 mL) and the crude product was purified by semi-preparative HPLC. The fraction containing *N*-(3-[^{125}I]iodobenzyl)guanidine was evaporated to dryness, then reconstituted in 10.0% ethanol in 0.9% saline to afford *N*-(3-[^{125}I]iodobenzyl)guanidine in 28% radioactivity yield (estimated using the measured radioactivity of the isolated product). The injected MIBG was below the UV detection limits and therefore the molar activity was calculated using the lowest concentration of *N*-(3-[^{127}I]iodobenzyl)guanidine which could be detected. Using this value the molar activity was estimated to be greater than 2.73 GBq/μmol. The radiochemical purity of the final product was determined by analytical HPLC and was >98%. The identity of the product was confirmed by comparing the retention time of *N*-(3-[^{125}I]iodobenzyl)guanidine against the retention time *N*-(3-[^{127}I]iodobenzyl)guanidine.

5. ^{1}H and ^{13}C Spectra

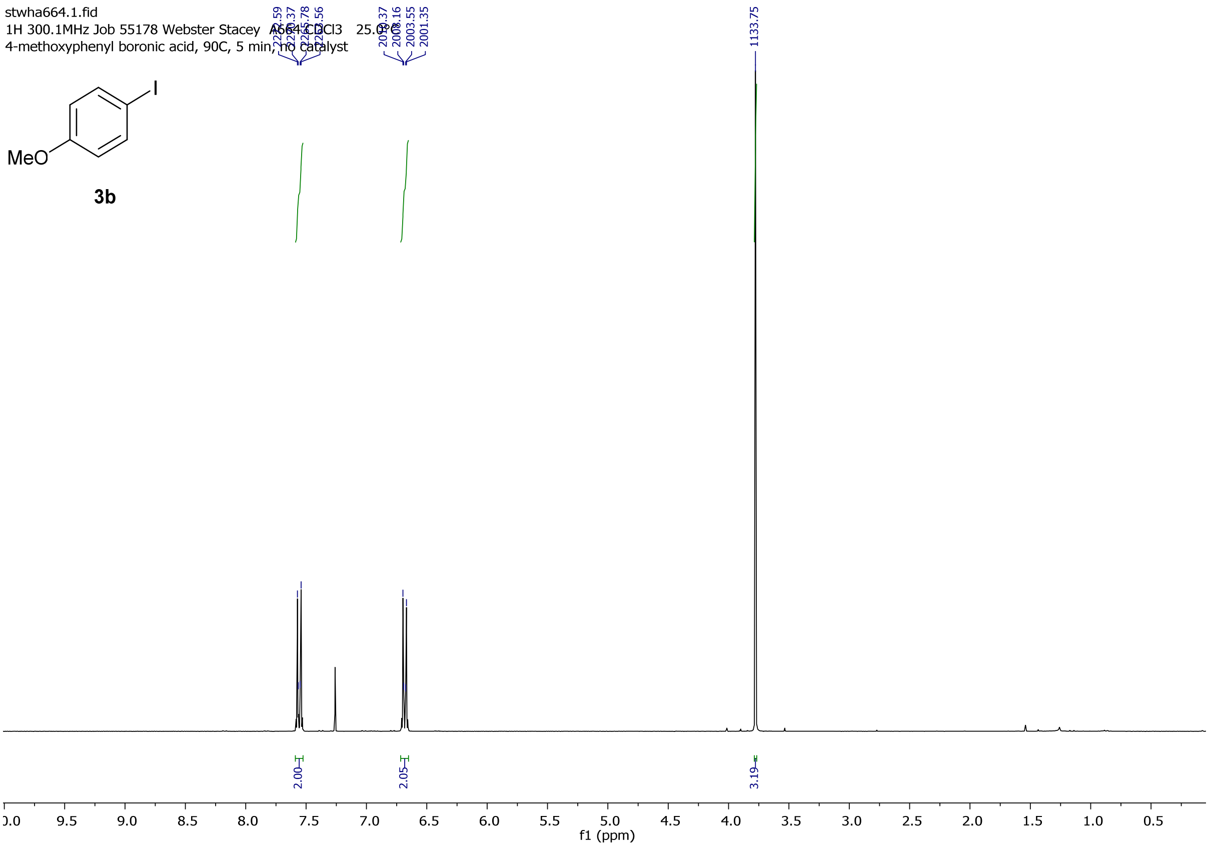

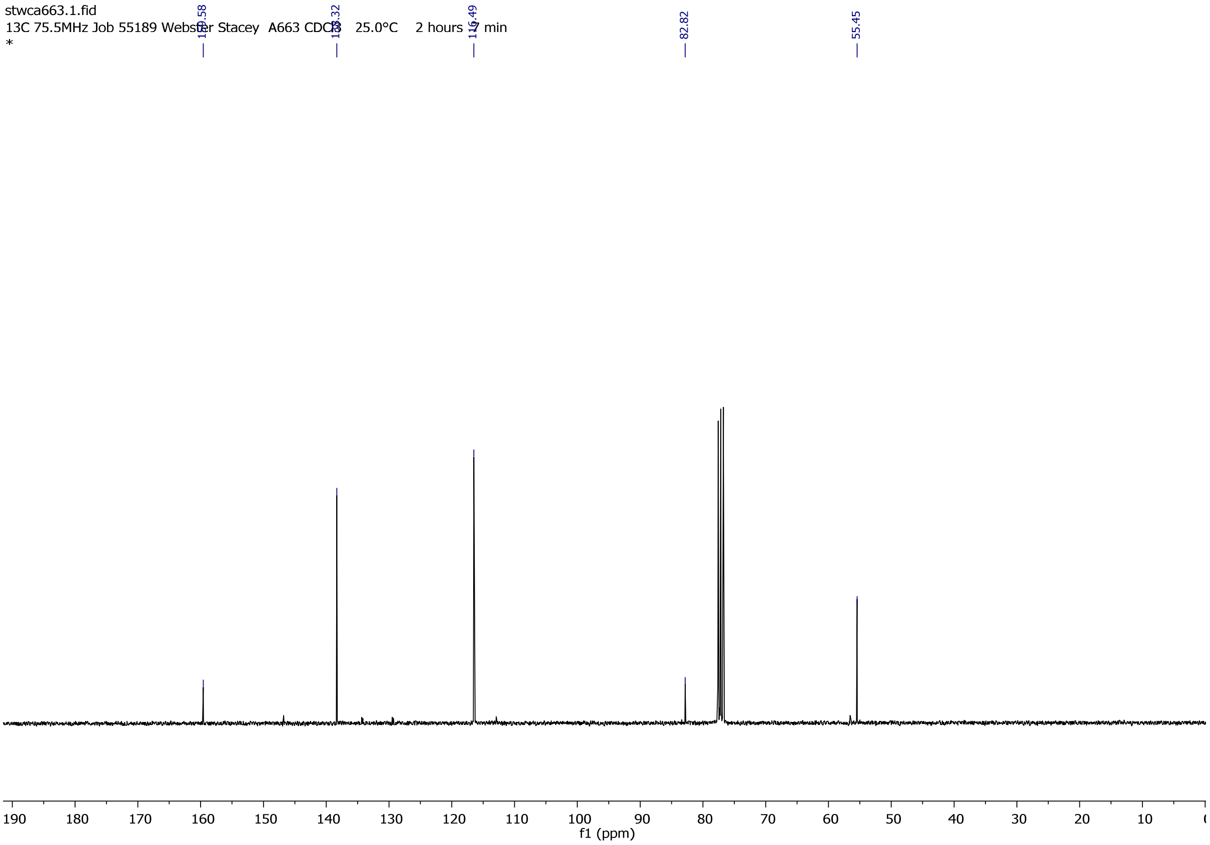

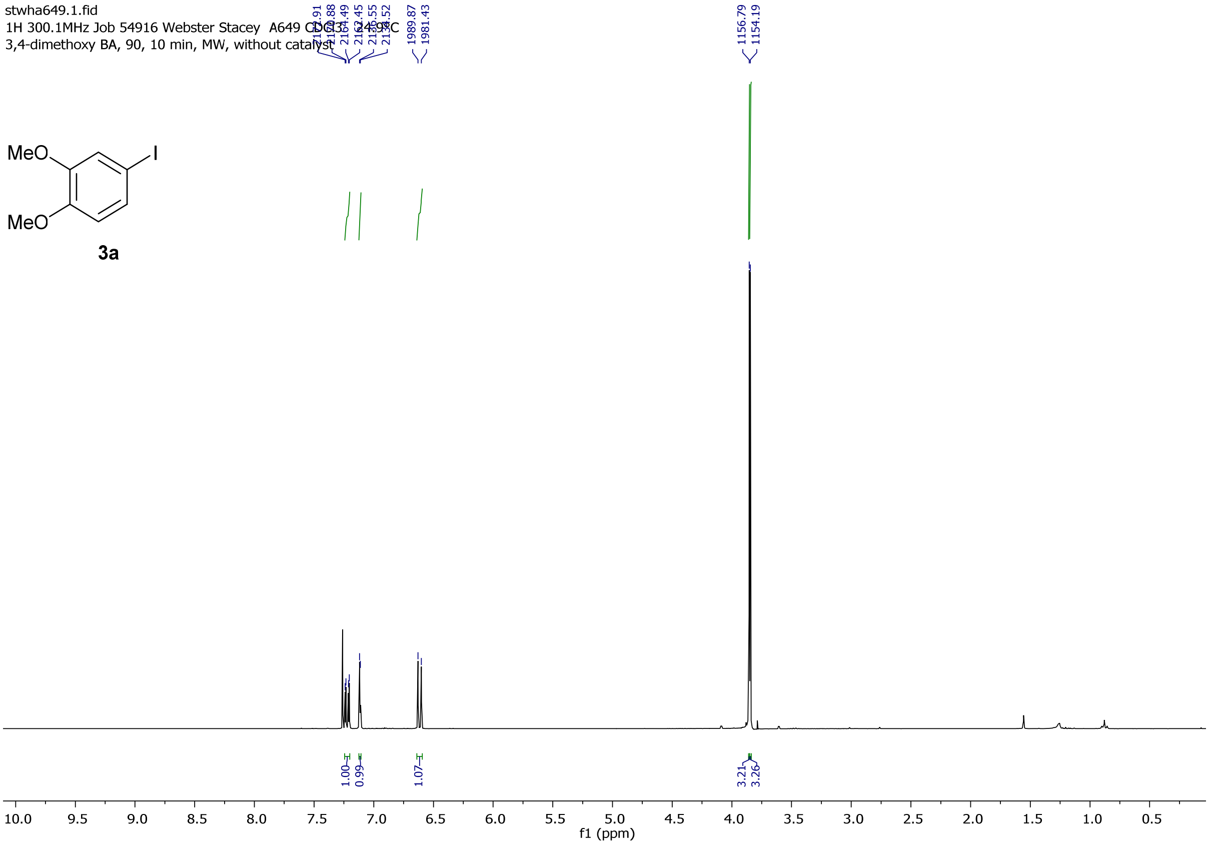
stwha649.1.fid
3,4-dimethoxy BA, 90, 10 min, MW, without catalyst
1989.87
1981.43
1156.79
1154.19
MeO
MeO
I
3a
1.00
0.99
1.07
3.21
3.26
10.0 9.5 9.0 8.5 8.0 7.5 7.0 6.5 6.0 5.5 5.0 4.5 4.0 3.5 3.0 2.5 2.0 1.5 1.0 0.5
f1 (ppm)

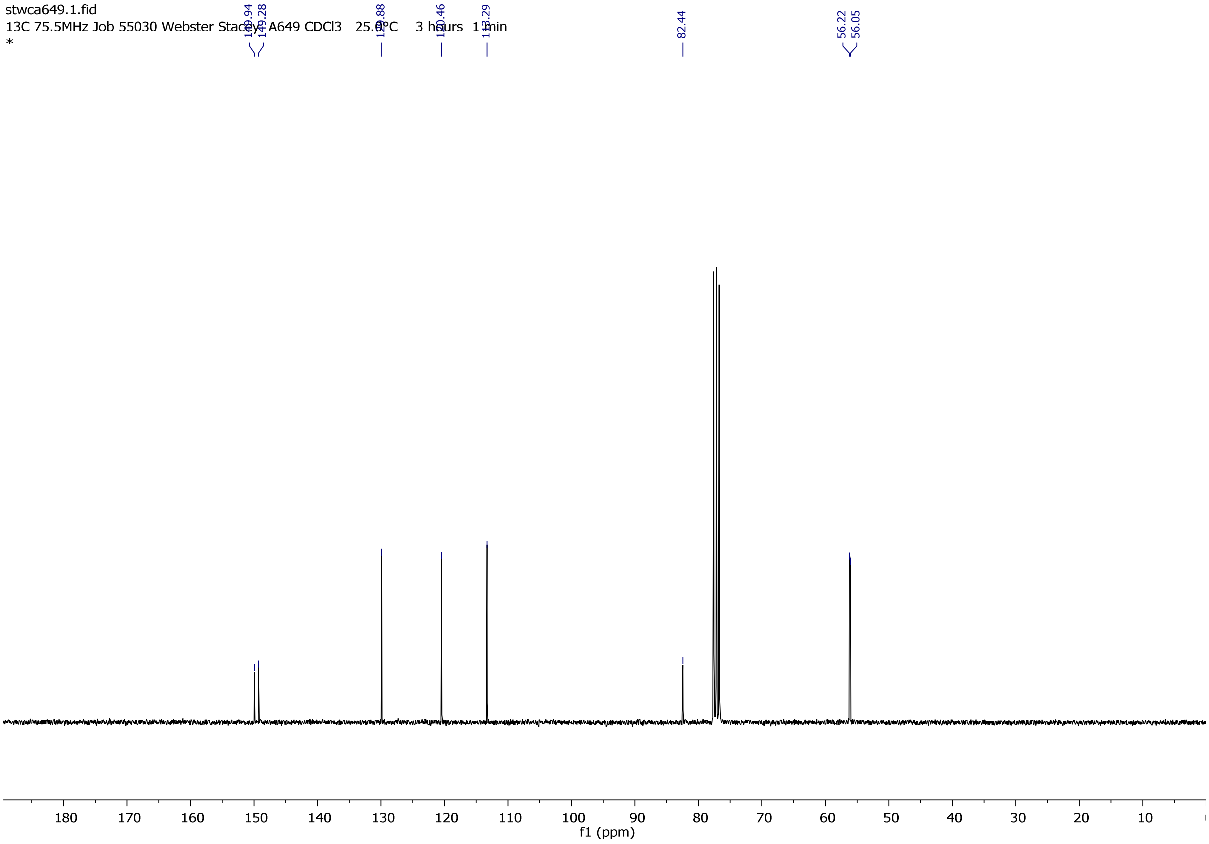
stwca649.1.fid
13C 75.5MHz Job 55030 Webster Stacey A649 CDCl3 25.0°C 3 hours 1 min
*
82.44
56.22
56.05
180 170 160 150 140 130 120 110 100 90 80 70 60 50 40 30 20 10
f1 (ppm)

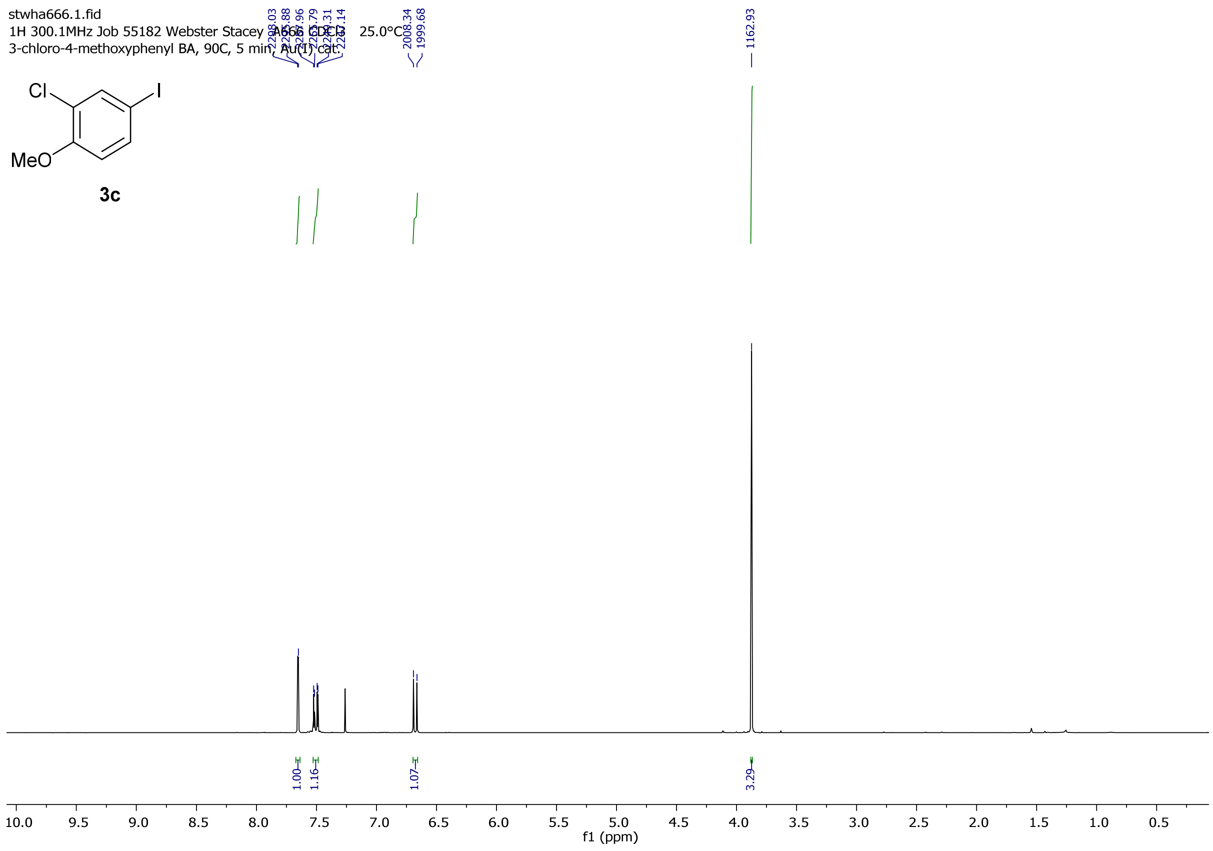
stwha666.1.fid
1H 300.1MHz Job 55182 Webster Stacey
25.0°C
3-chloro-4-methoxyphenyl BA, 90C, 5 min
Cl
I
MeO
3c
f1 (ppm)

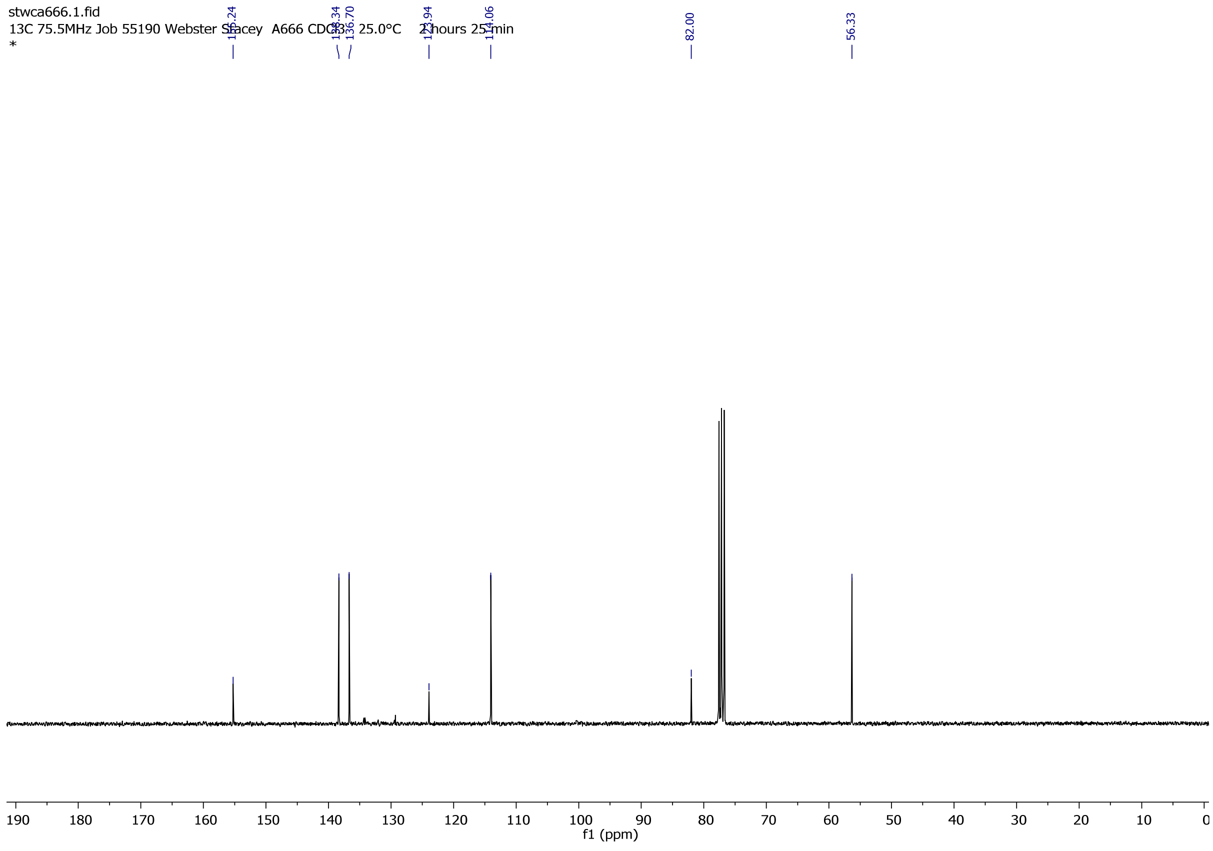
stwca666.1.fid
13C 75.5MHz Job 55190 Webster Stacey A666
25.0°C
*
82.00
56.33
f1 (ppm)

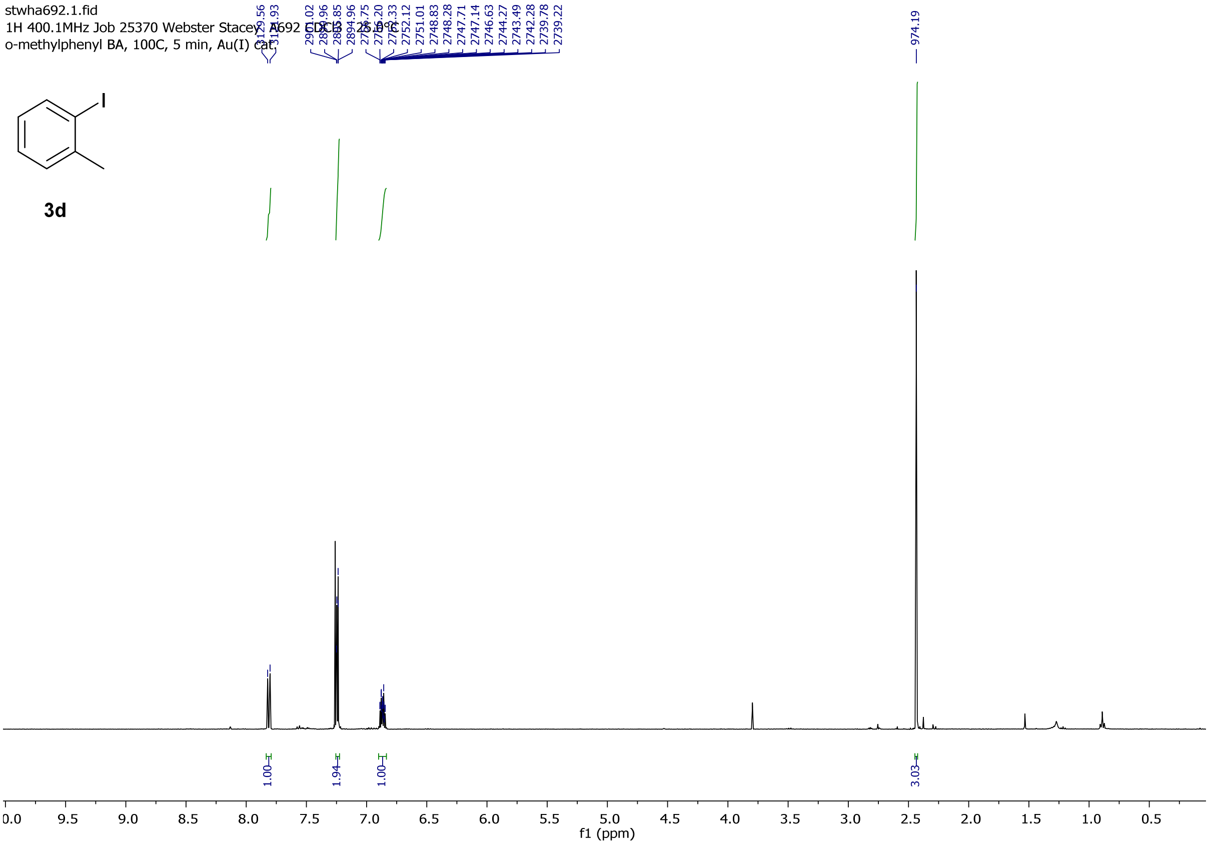
stwha692.1.fid
o-methylphenyl BA, 100C, 5 min, Au(I) cat.
3d
974.19
1.00
1.94
1.00
3.03
f1 (ppm)

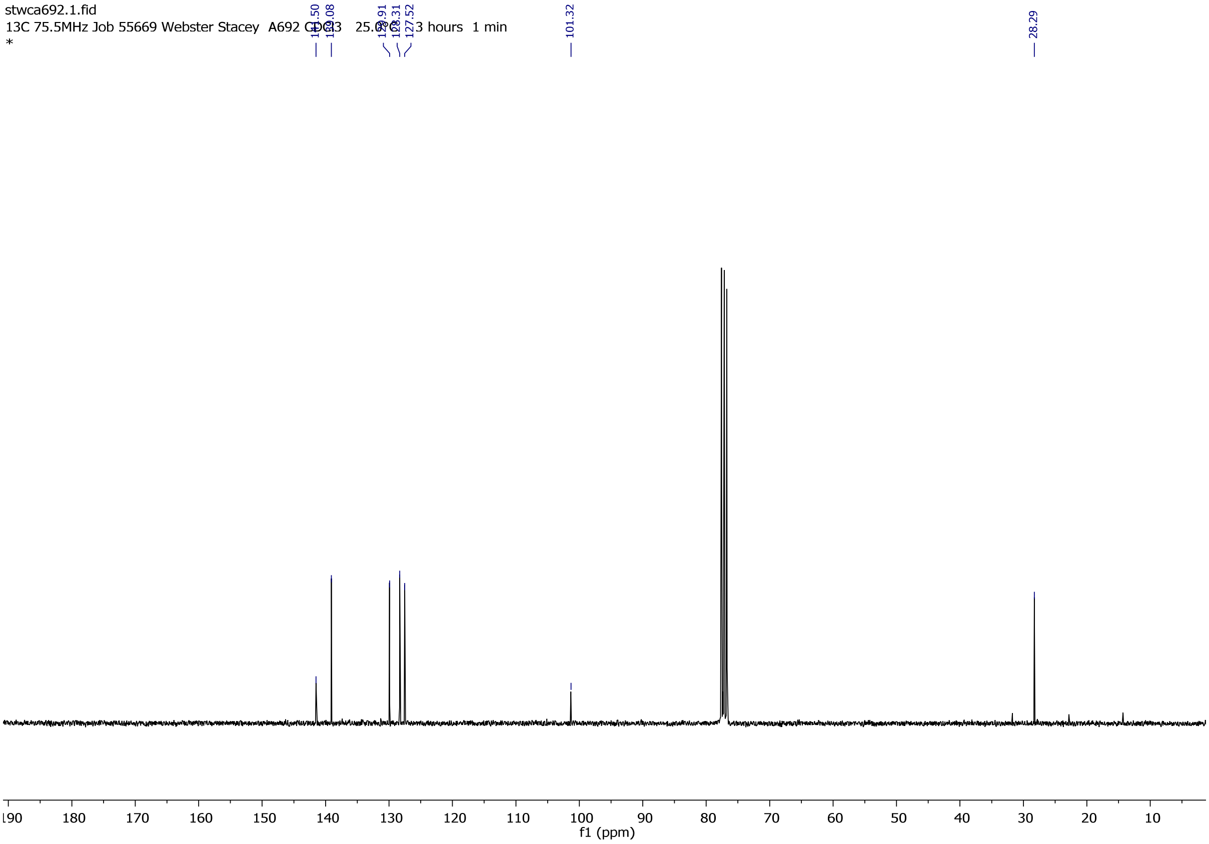
stwca692.1.fid
*
127.52
101.32
28.29
f1 (ppm)

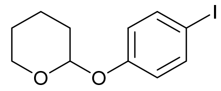

3f

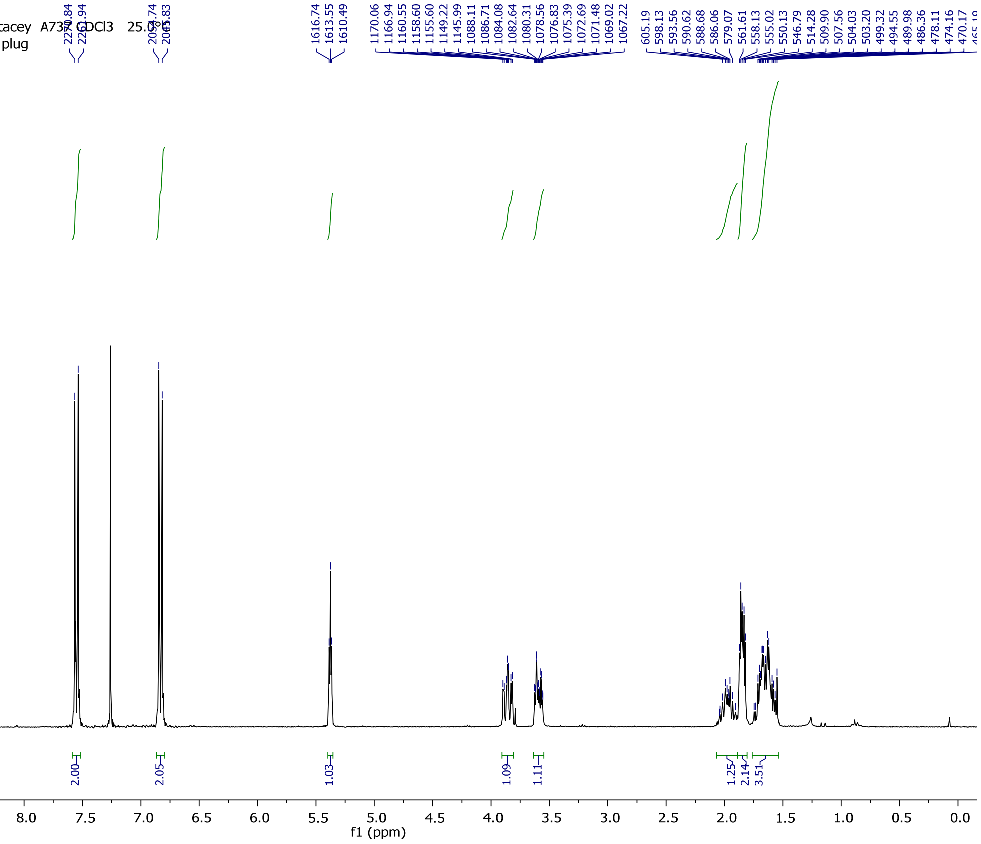
stwha737.1.fid
1H 300.1MHz Job 56487 Webster Stacey A737 CDCl3 25.0°C
THP BA, MS, 90 C, 5 mins, alumina plug
2.00
2.05
1.03
1.09
1.11
1.25
2.14
3.51
f1 (ppm)

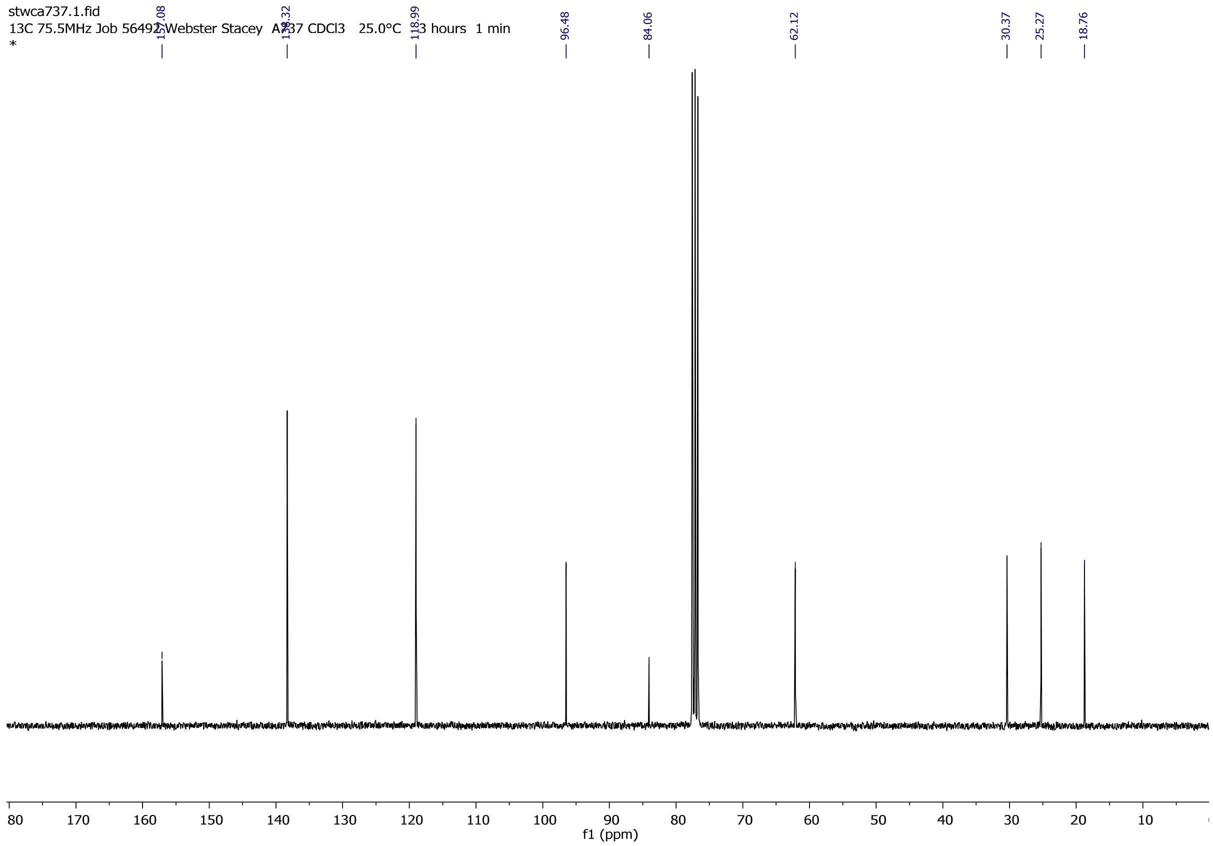
stwca737.1.fid
13C 75.5MHz Job 56492 Webster Stacey A737 CDCl3 25.0°C 3 hours 1 min
*
96.48
84.06
62.12
30.37
25.27
18.76
f1 (ppm)

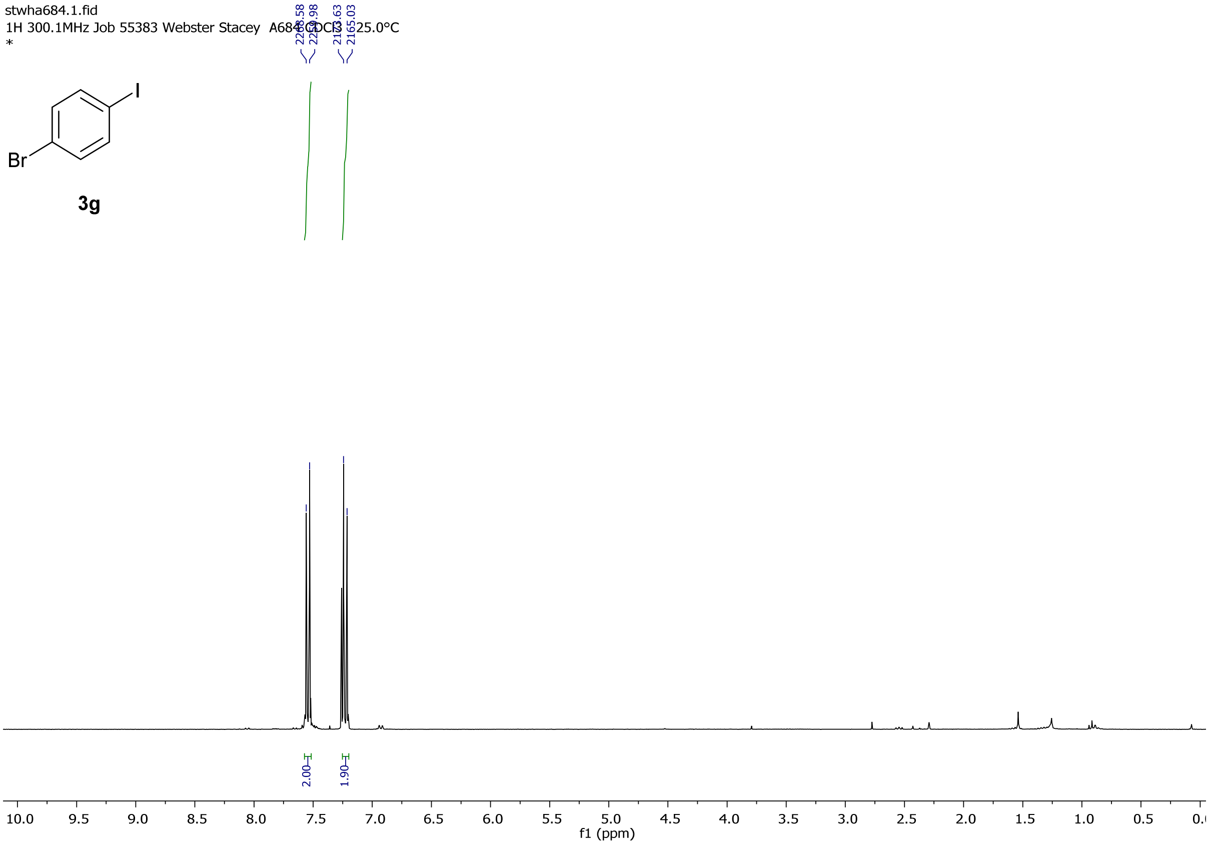
stwha684.1.fid
1H 300.1MHz Job 55383 Webster Stacey A684
Br
I
3g
2.00
1.90
f1 (ppm)

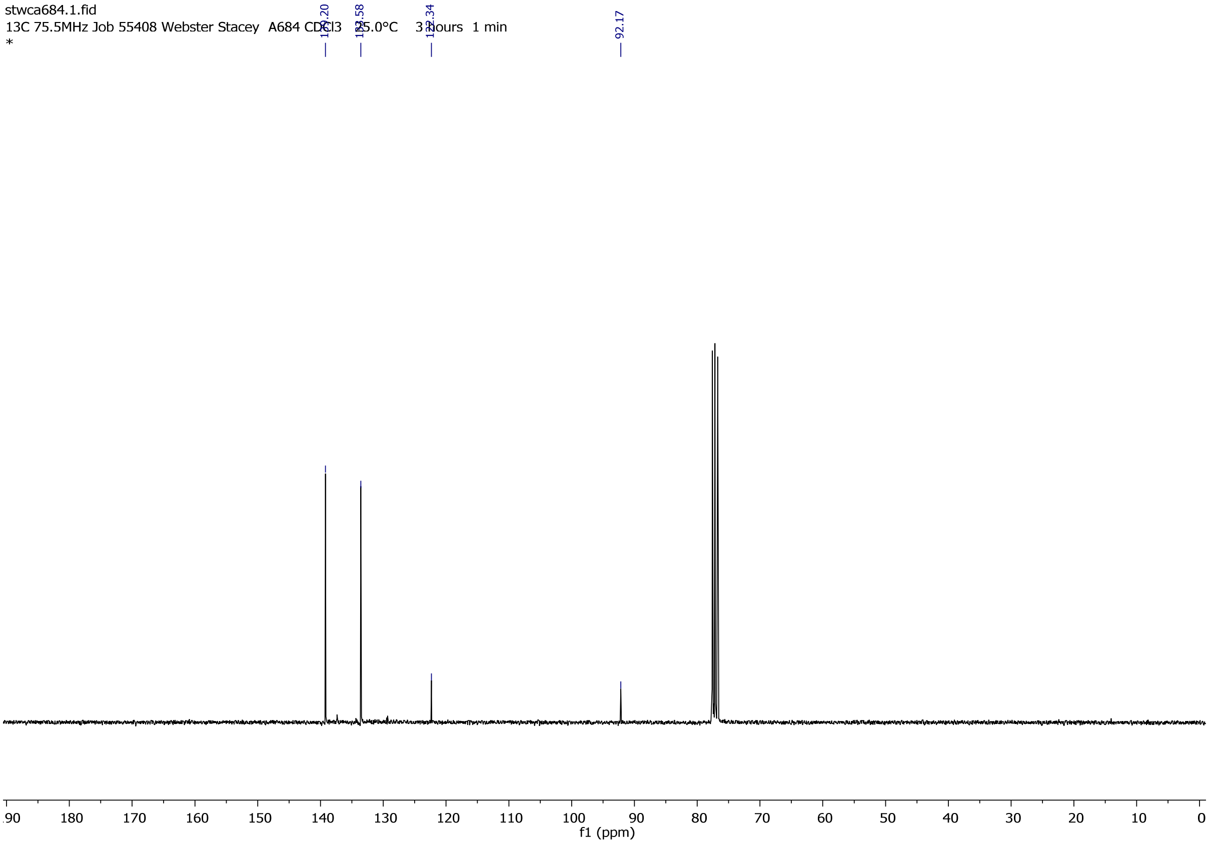
stwca684.1.fid
13C 75.5MHz Job 55408 Webster Stacey A684
92.17
f1 (ppm)

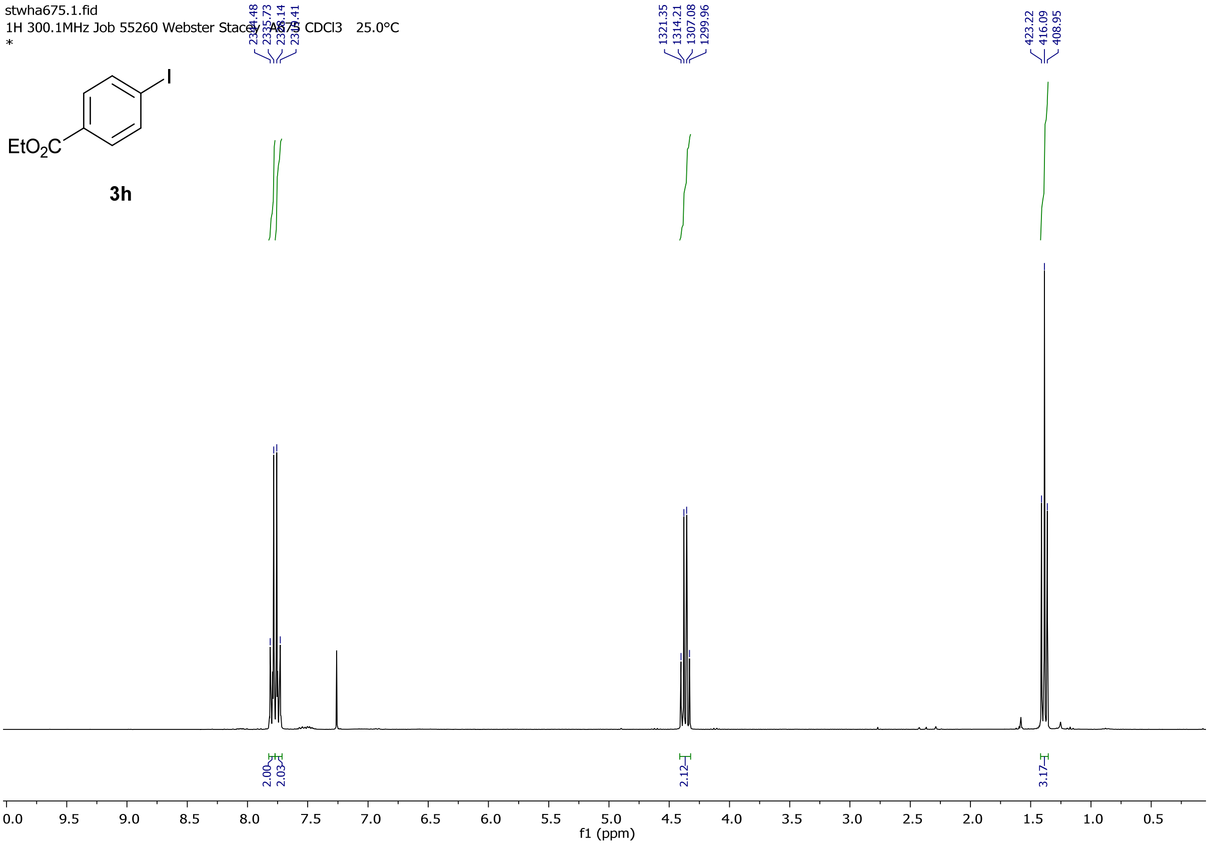
stwha675.1.fid
1H 300.1MHz Job 55260 Webster Stacey
CDCl3 25.0°C
EtO2C
I
3h
2335.73
1321.35
1314.21
1307.08
1299.96
423.22
416.09
408.95
2.00
2.03
2.12
3.17
f1 (ppm)

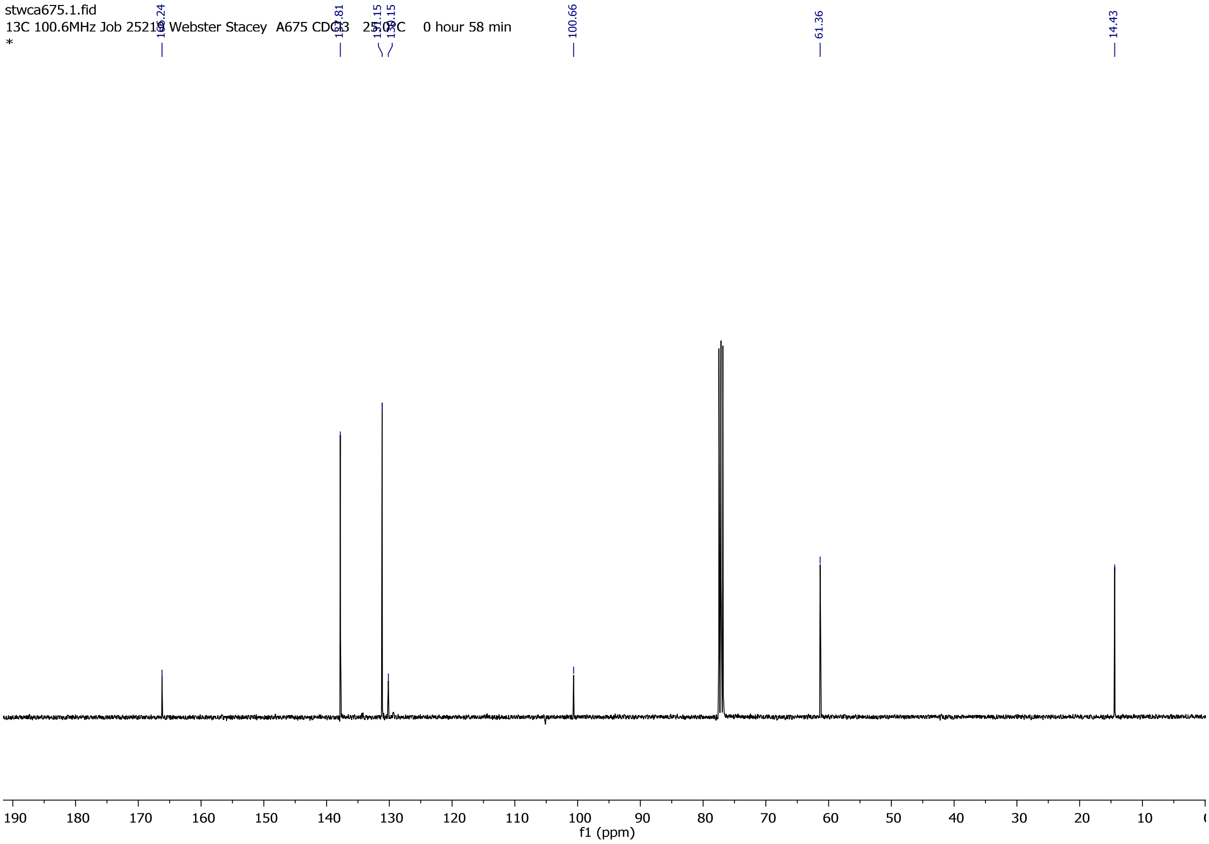
stwca675.1.fid
13C 100.6MHz Job
Webster Stacey A675
0 hour 58 min
100.66
61.36
14.43
f1 (ppm)

stwha679.1.fid
1H 300.1MHz Job 55345 Webster Stacey A679 CDCl3 25.0°C
*

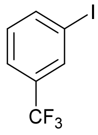

3i

Volatile product

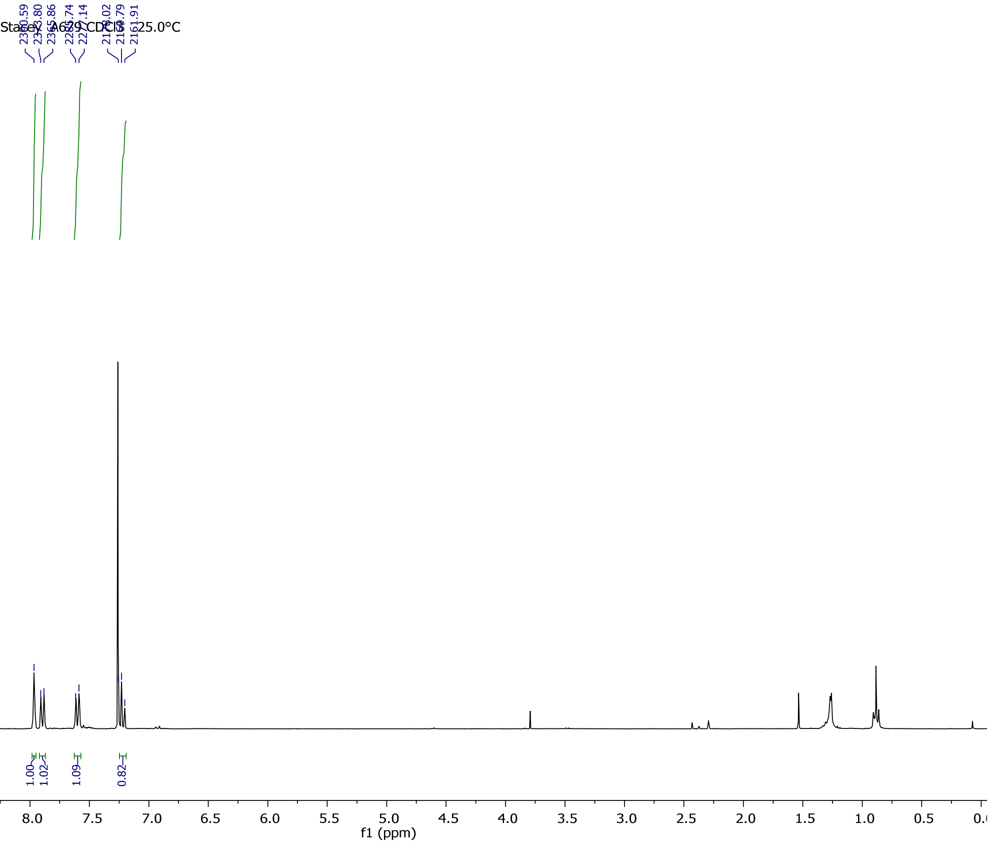

stwca679.1.fid
13C 75.5MHz Job 55366 Webster Stacey A679 CDCl3 25.0°C 3 hours 1 min
*

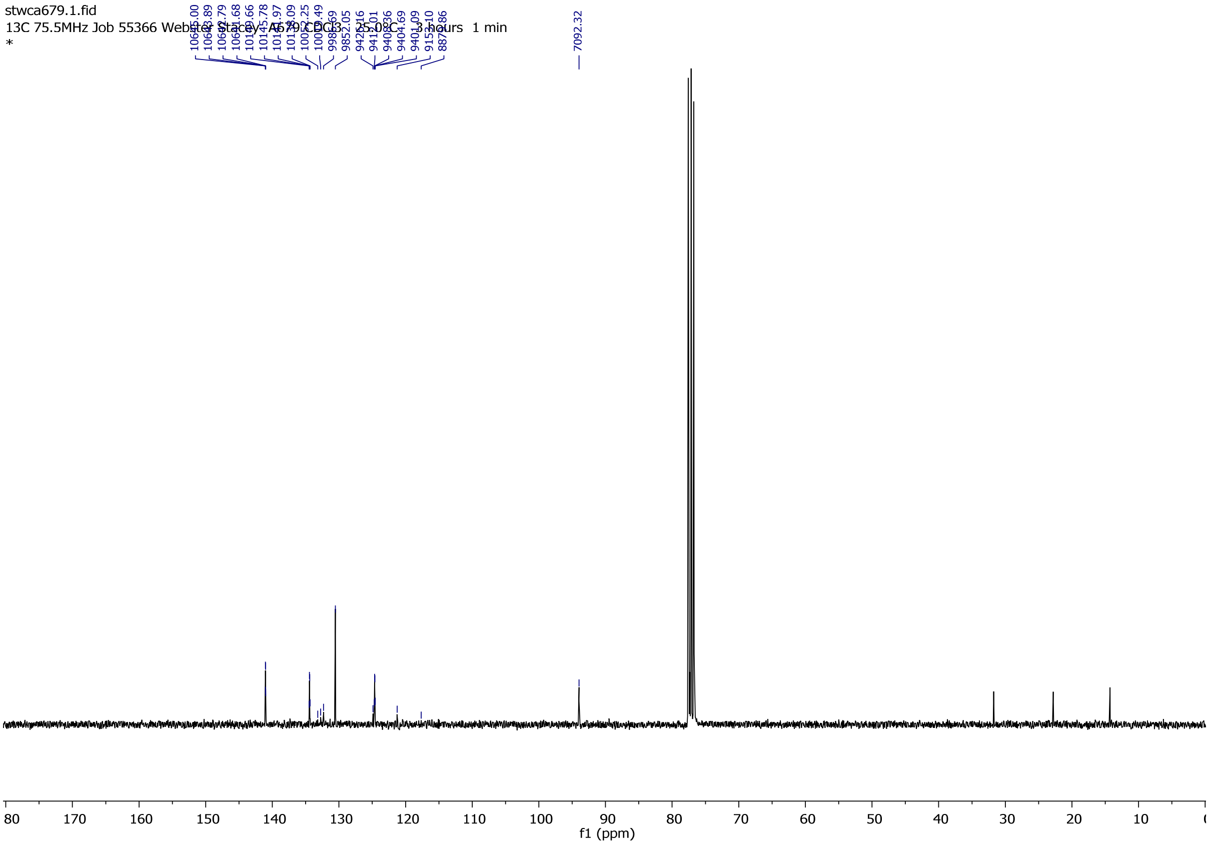

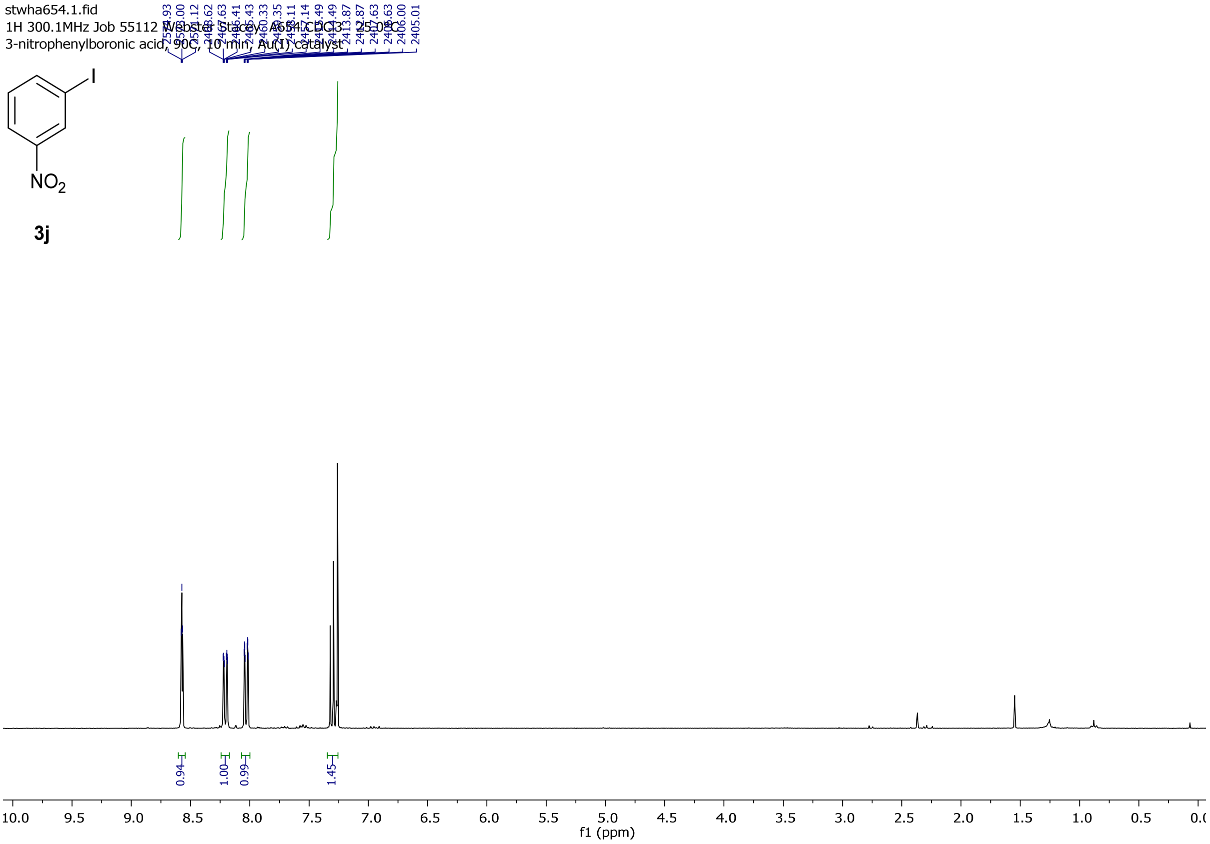
stwha654.1.fid
1H 300.1MHz Job 55112
3-nitrophenylboronic acid
I
NO_2
3j
0.94
1.00
0.99
1.45
f1 (ppm)

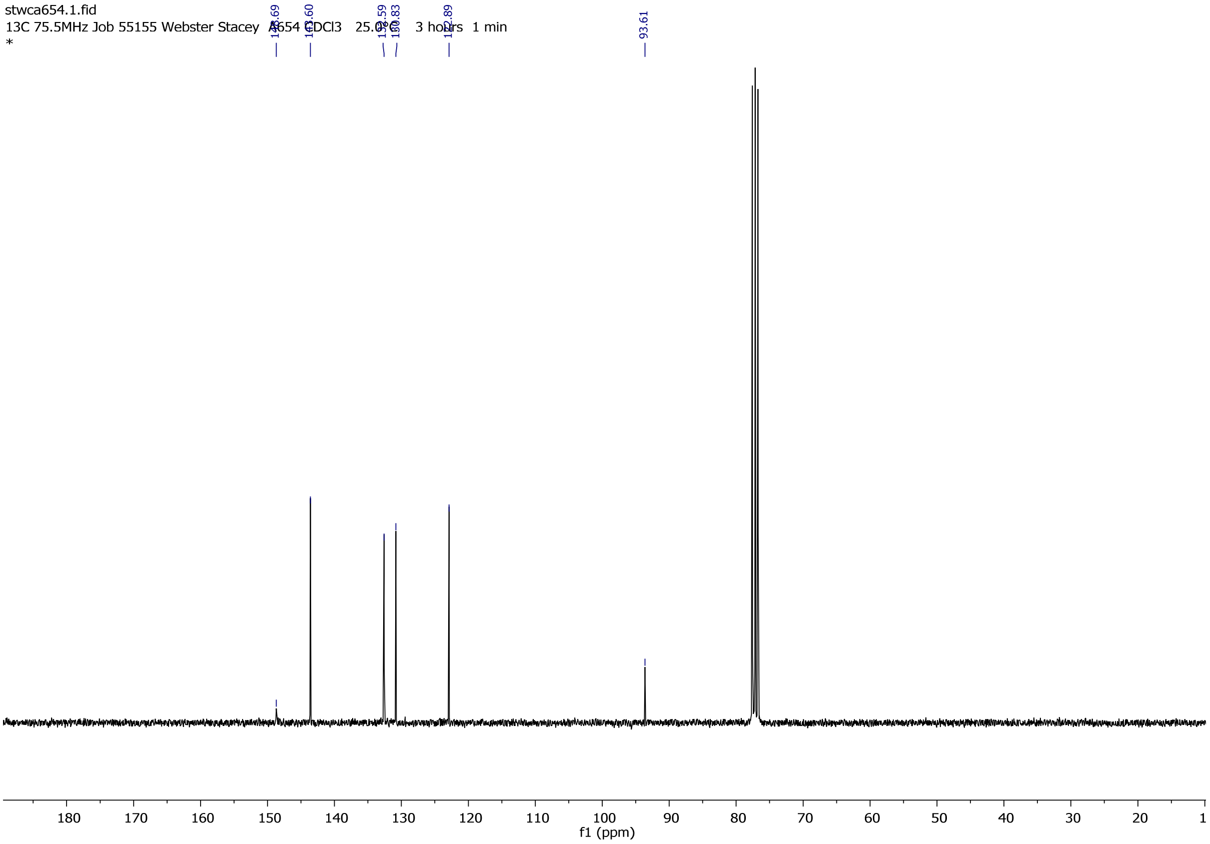
stwca654.1.fid
13C 75.5MHz Job 55155 Webster Stacey
*
93.61
f1 (ppm)

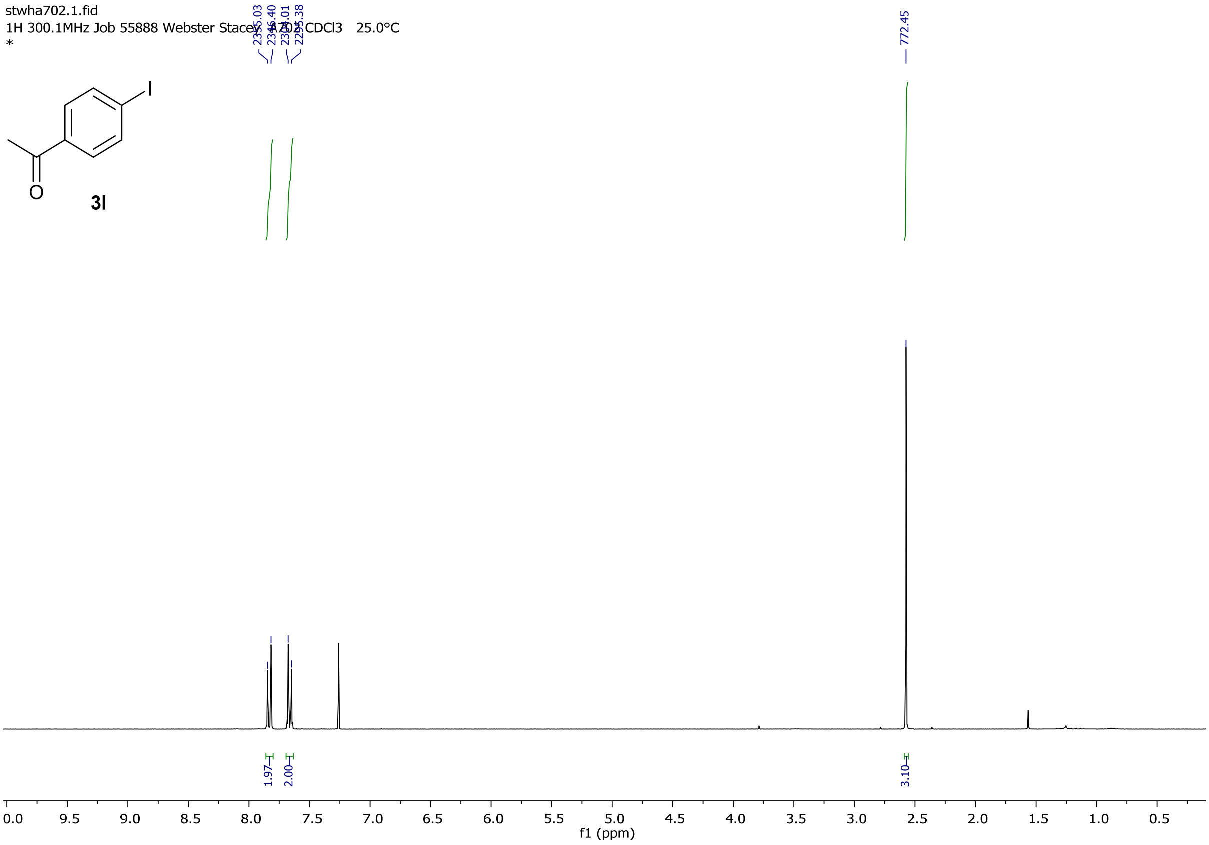
stwha702.1.fid
1H 300.1MHz Job 55888 Webster Stacey
CDCl3 25.0°C
772.45
3l
1.97
2.00
3.10
f1 (ppm)

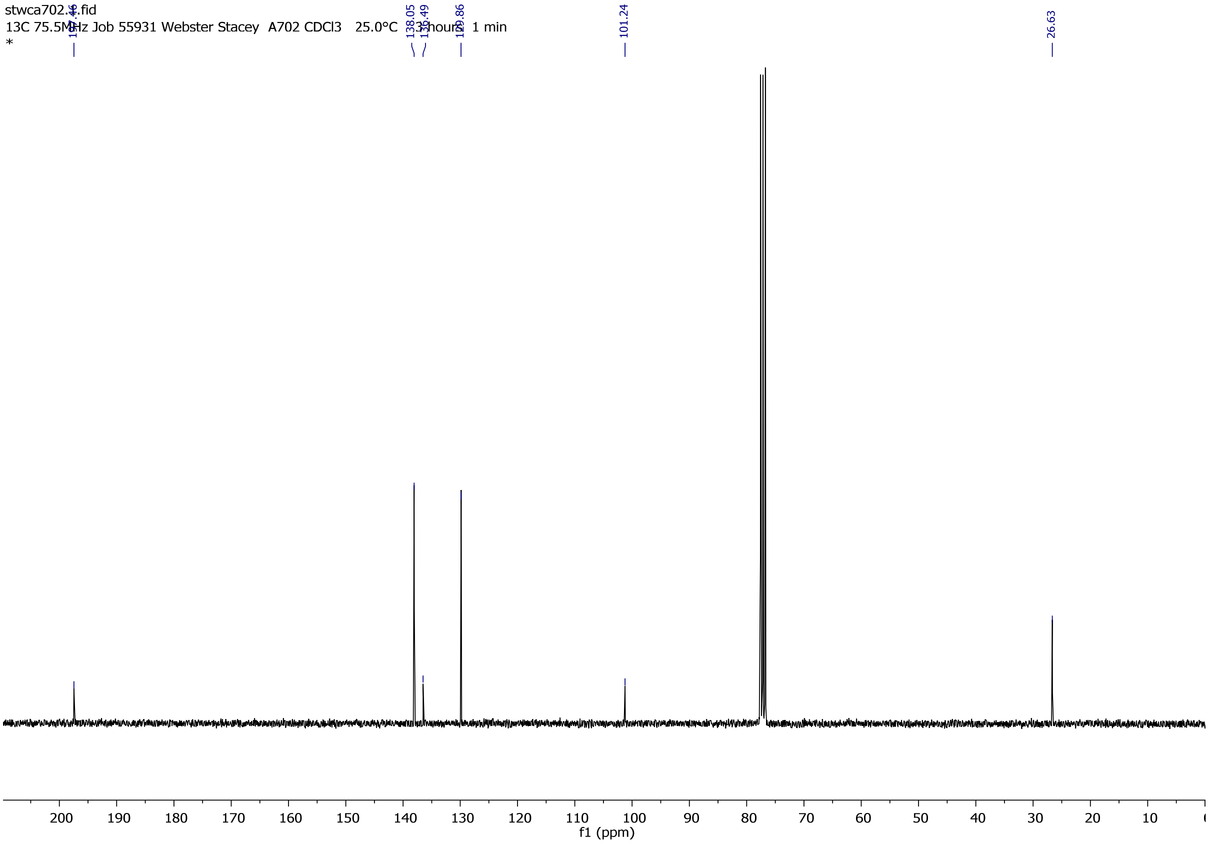
13C 75.5M
Job 55931 Webster Stacey A702 CDCl3 25.0°C
1 min
138.05
101.24
26.63
f1 (ppm)

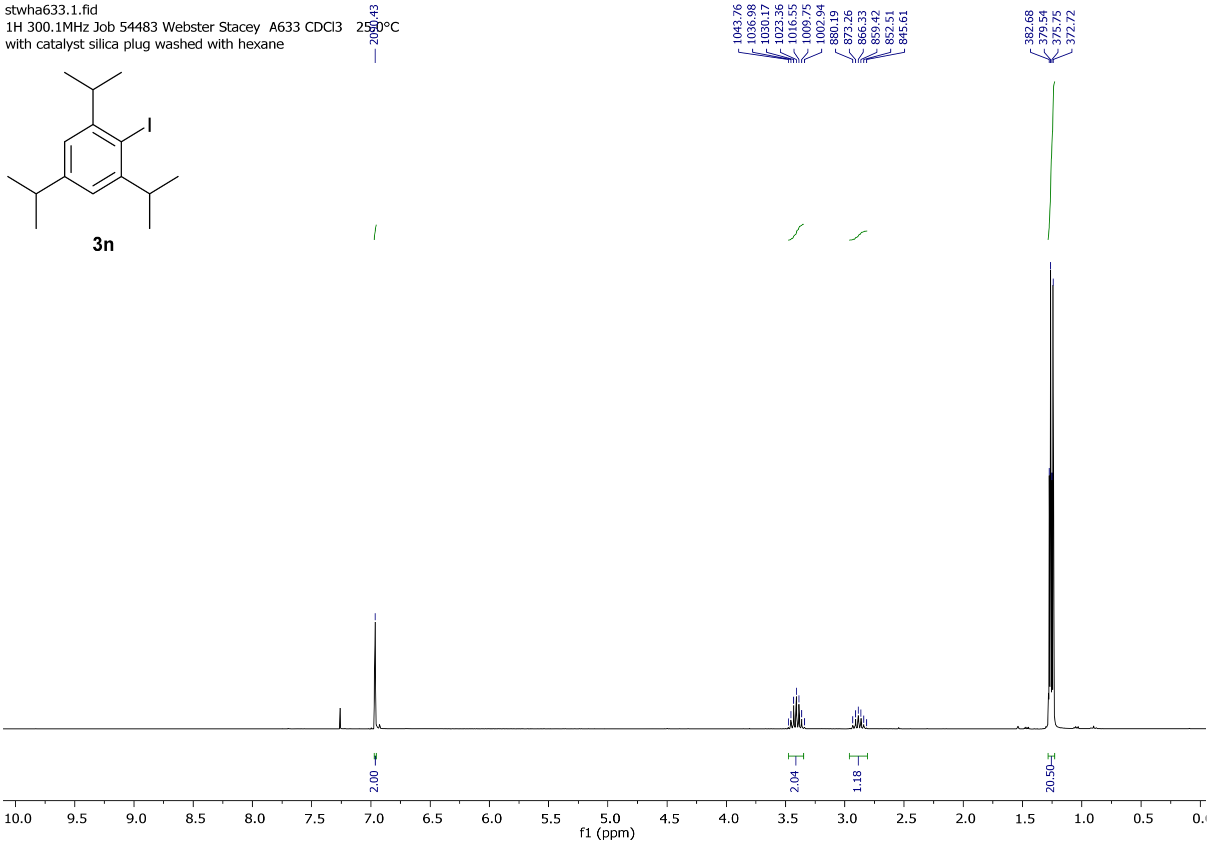
stwha633.1.fid
1H 300.1MHz Job 54483 Webster Stacey A633 CDCl3 25.0°C
with catalyst silica plug washed with hexane
I
3n
1043.76
1036.98
1030.17
1023.36
1016.55
1009.75
1002.94
880.19
873.26
866.33
859.42
852.51
845.61
382.68
379.54
375.75
372.72
2.00
2.04
1.18
20.50
f1 (ppm)

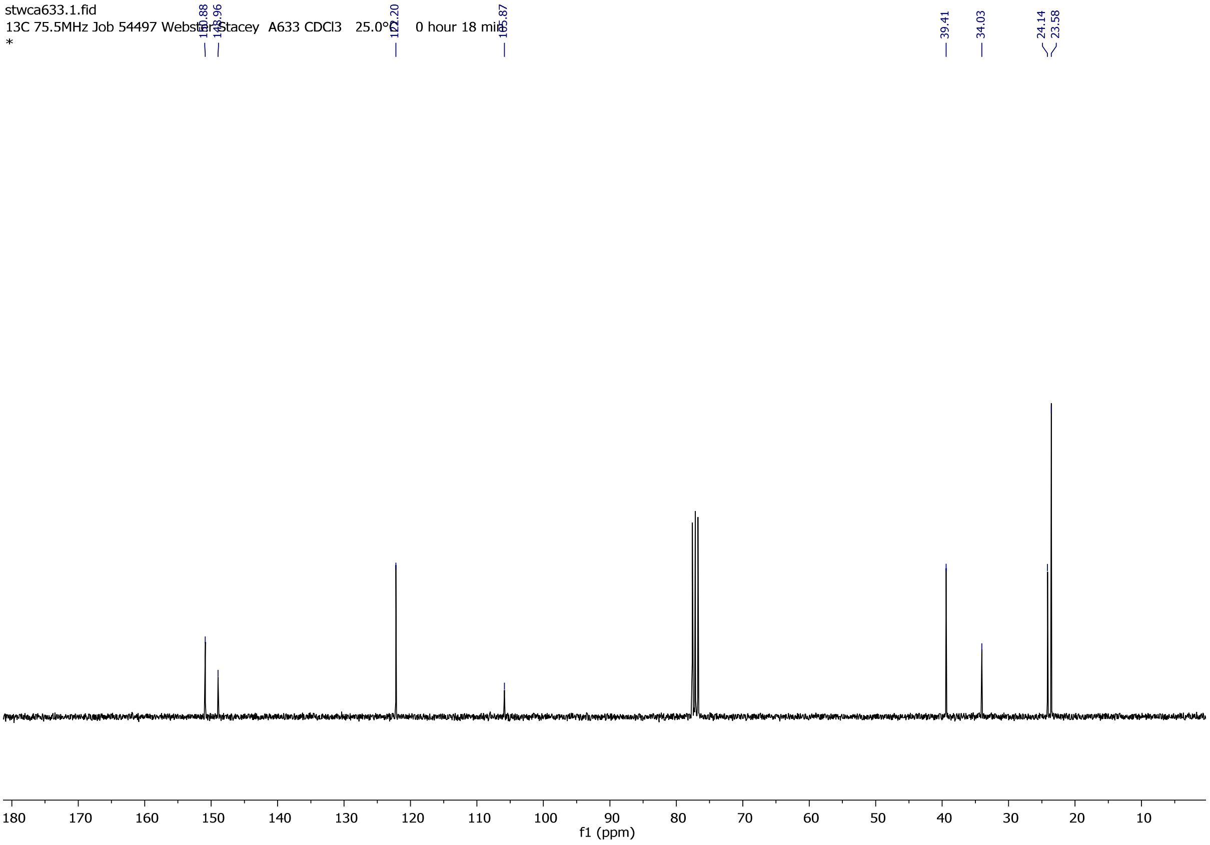
stwca633.1.fid
13C 75.5MHz Job 54497 Webster Stacey A633 CDCl3 25.0°C 0 hour 18 min
*
39.41
34.03
24.14
23.58
f1 (ppm)

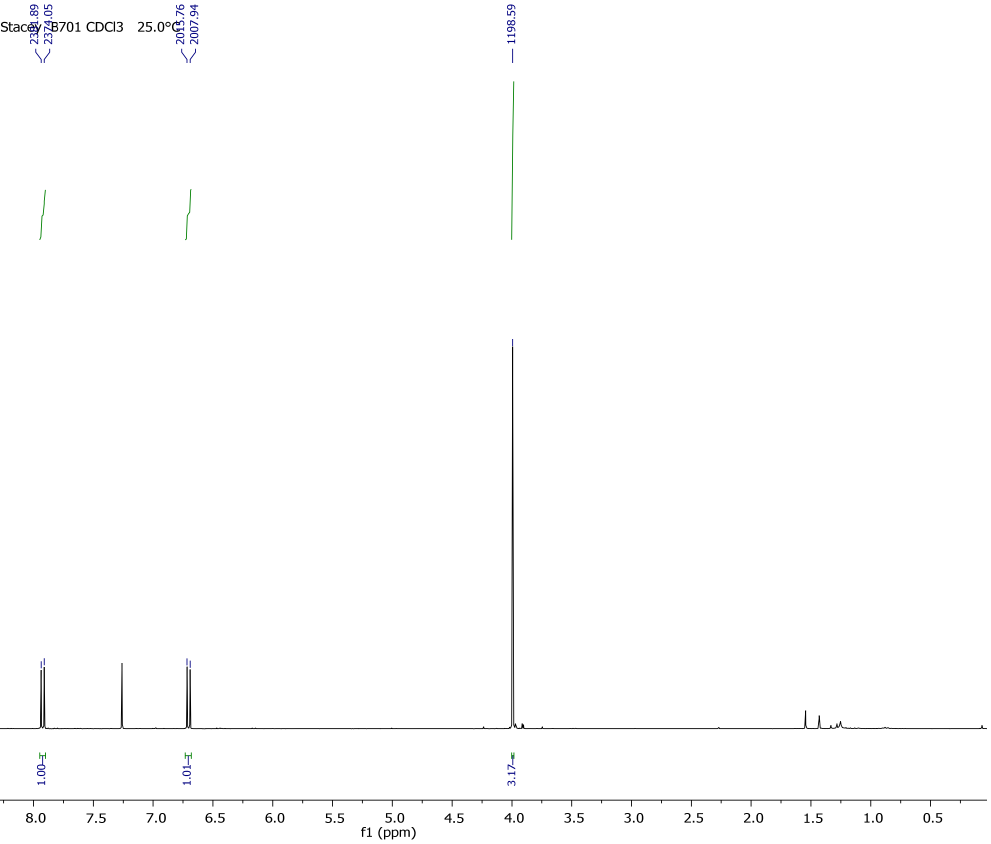
stwhb701.1.fid
1H 300.1MHz Job 55884 Webster Stacey B701 CDCl3 25.0°C
*
2381.89
2374.05
2013.76
2007.94
1198.59
1.00
1.01
3.17
10.0 9.5 9.0 8.5 8.0 7.5 7.0 6.5 6.0 5.5 5.0 4.5 4.0 3.5 3.0 2.5 2.0 1.5 1.0 0.5
f1 (ppm)

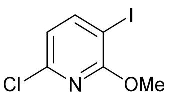
Cl
N
OMe
I

3o

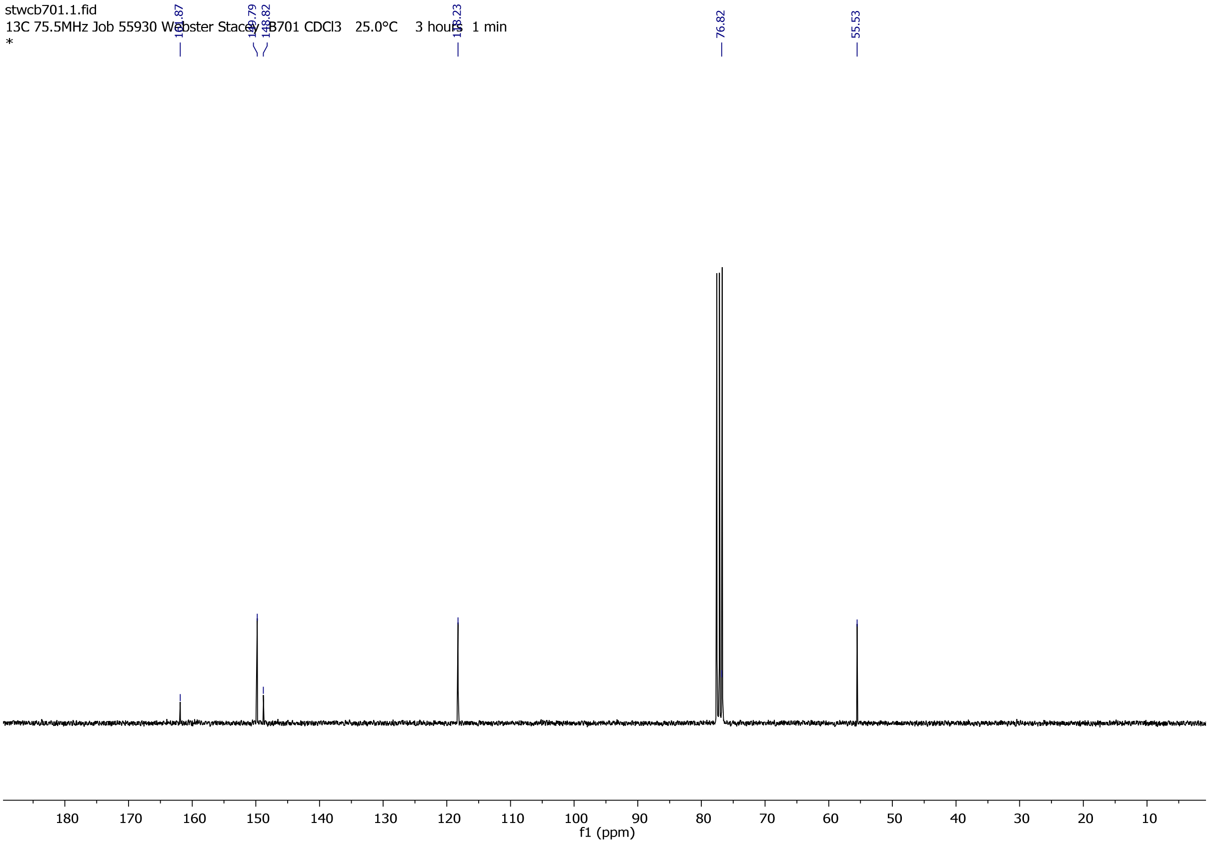
stwcb701.1.fid
13C 75.5MHz Job 55930 Webster Stacey B701 CDCl3 25.0°C 3 hours 1 min
*
76.82
55.53
180 170 160 150 140 130 120 110 100 90 80 70 60 50 40 30 20 10
f1 (ppm)

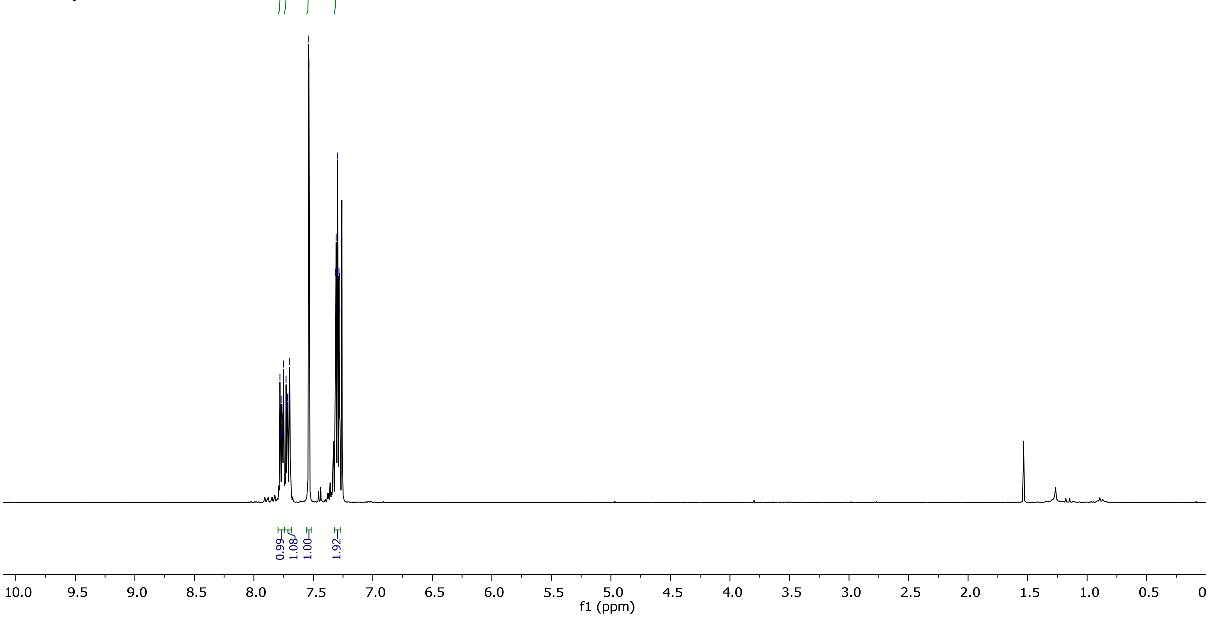

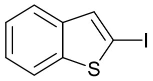

3p

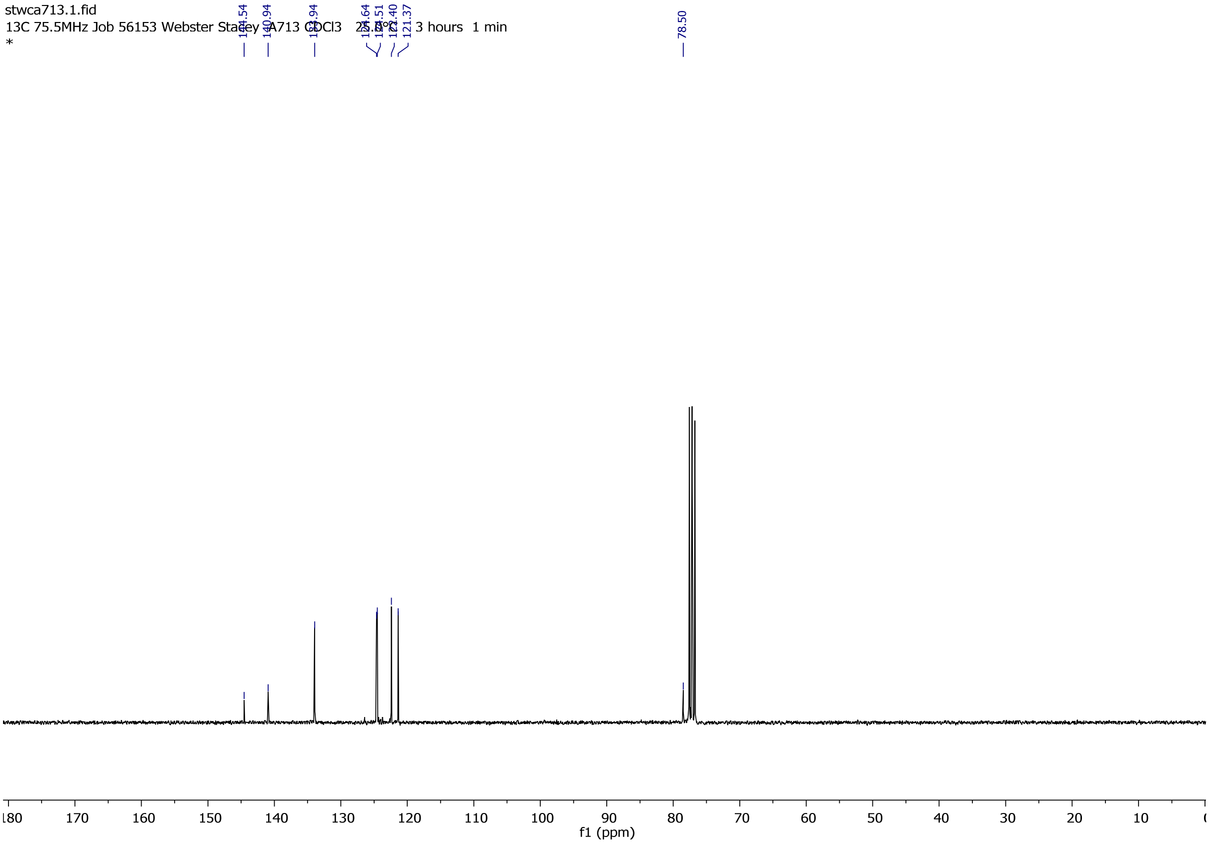

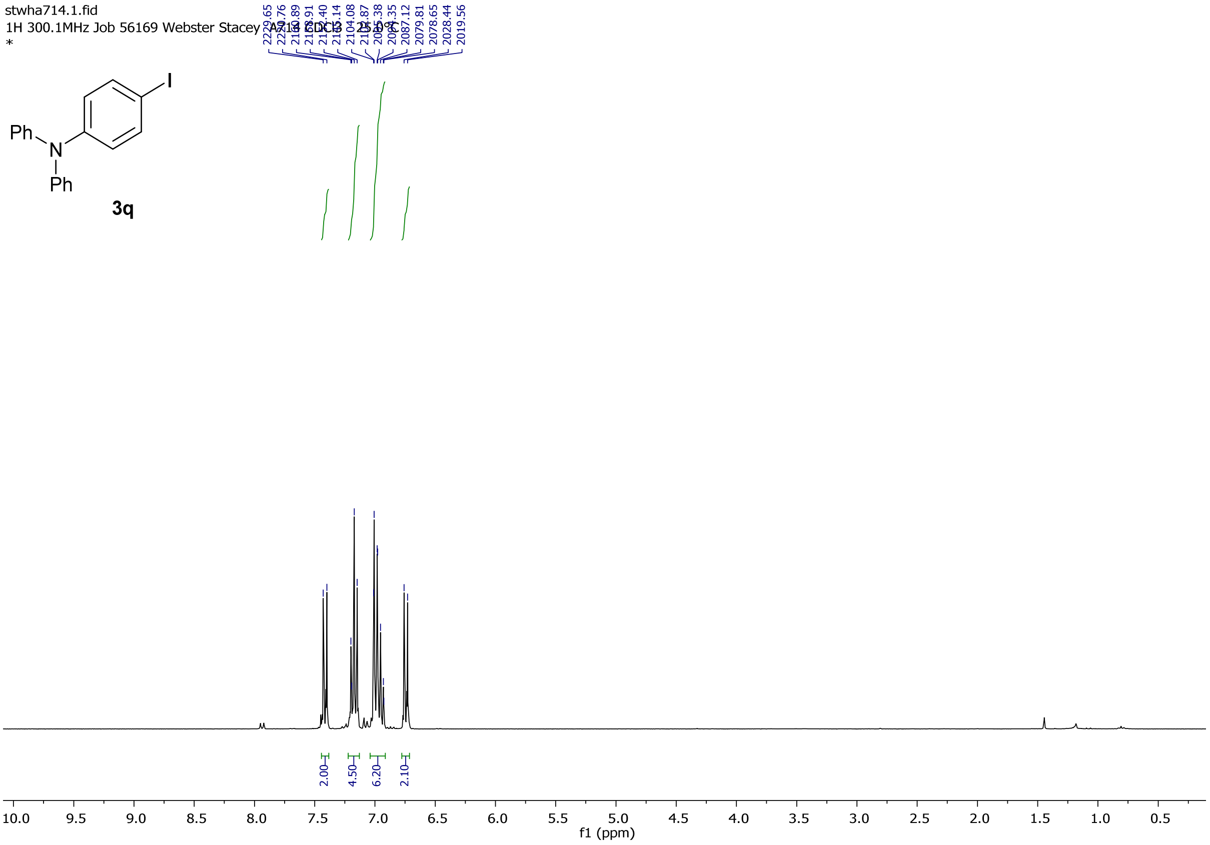
stwha714.1.fid
1H 300.1MHz Job 56169 Webster Stacey
*
Ph
N
Ph
I
3q
2.00
4.50
6.20
2.10
10.0
9.5
9.0
8.5
8.0
7.5
7.0
6.5
6.0
5.5
5.0
4.5
4.0
3.5
3.0
2.5
2.0
1.5
1.0
0.5
f1 (ppm)

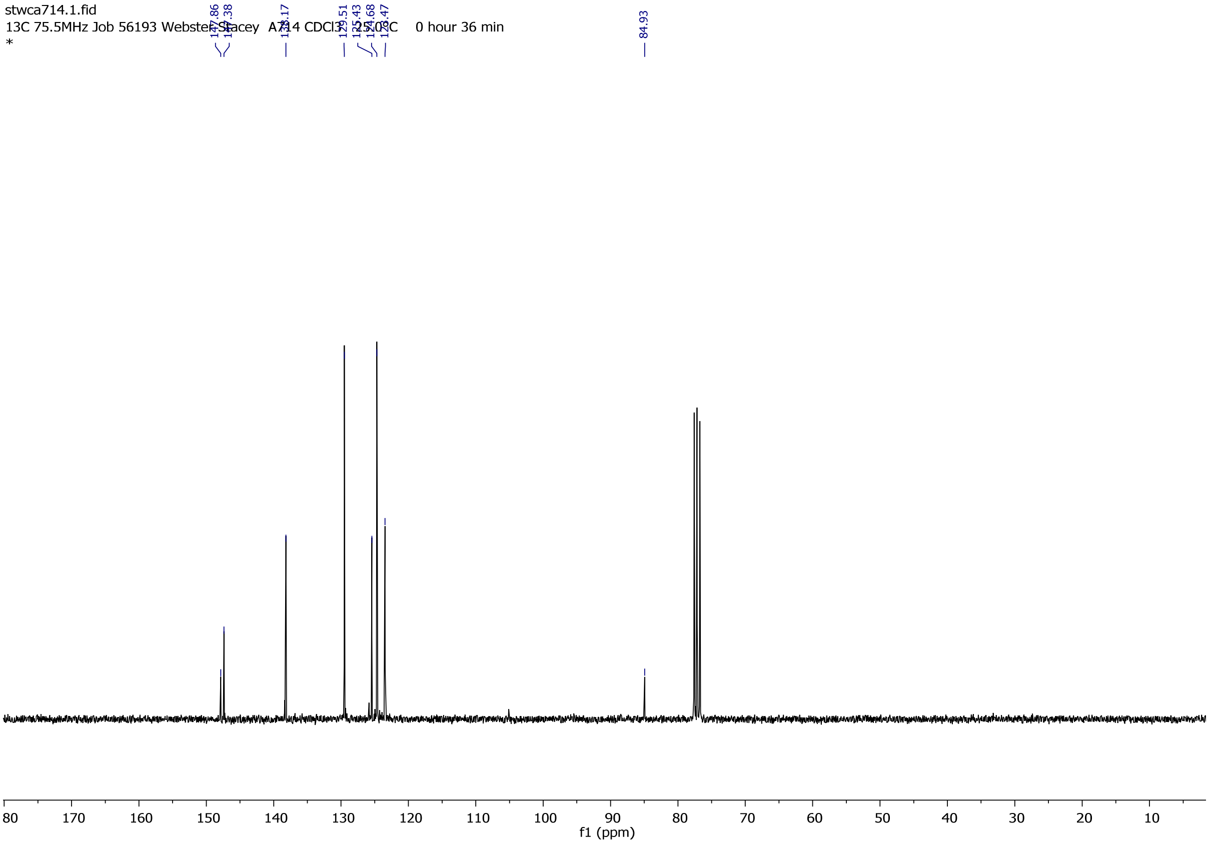
stwca714.1.fid
13C 75.5MHz Job 56193 Webster Stacey
0 hour 36 min
*
84.93
170
160
150
140
130
120
110
100
90
80
70
60
50
40
30
20
10
f1 (ppm)

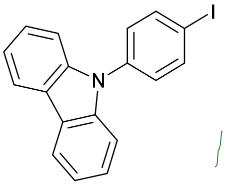

3r

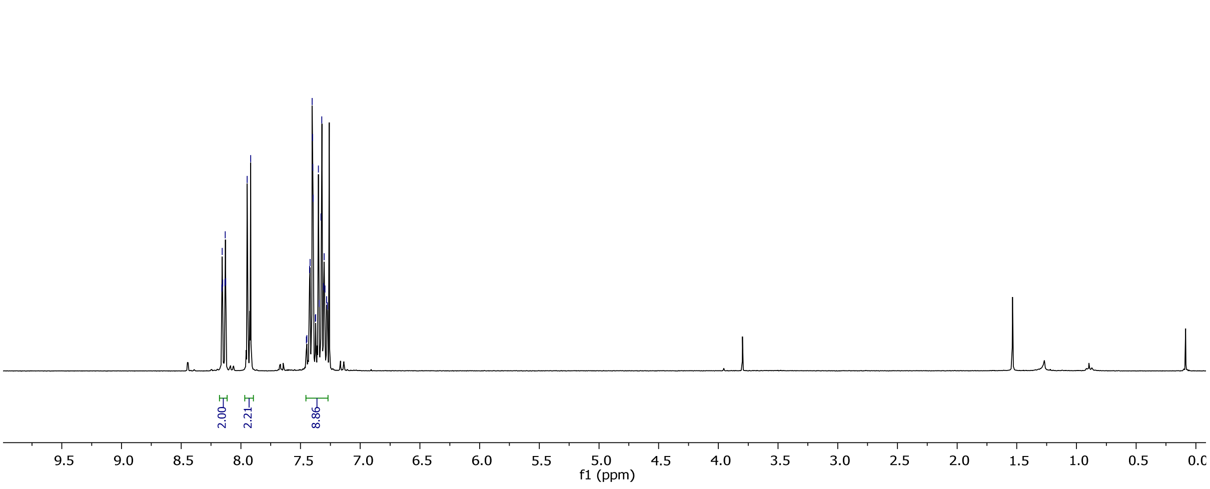
stwha766.1.fid
1H 300.1MHz Job 57233 Webster Stacey A766 CDCl3 24.9°C
*
2.00
2.21
8.86
9.5
9.0
8.5
8.0
7.5
7.0
6.5
6.0
5.5
5.0
4.5
4.0
3.5
3.0
2.5
2.0
1.5
1.0
0.5
f1 (ppm)

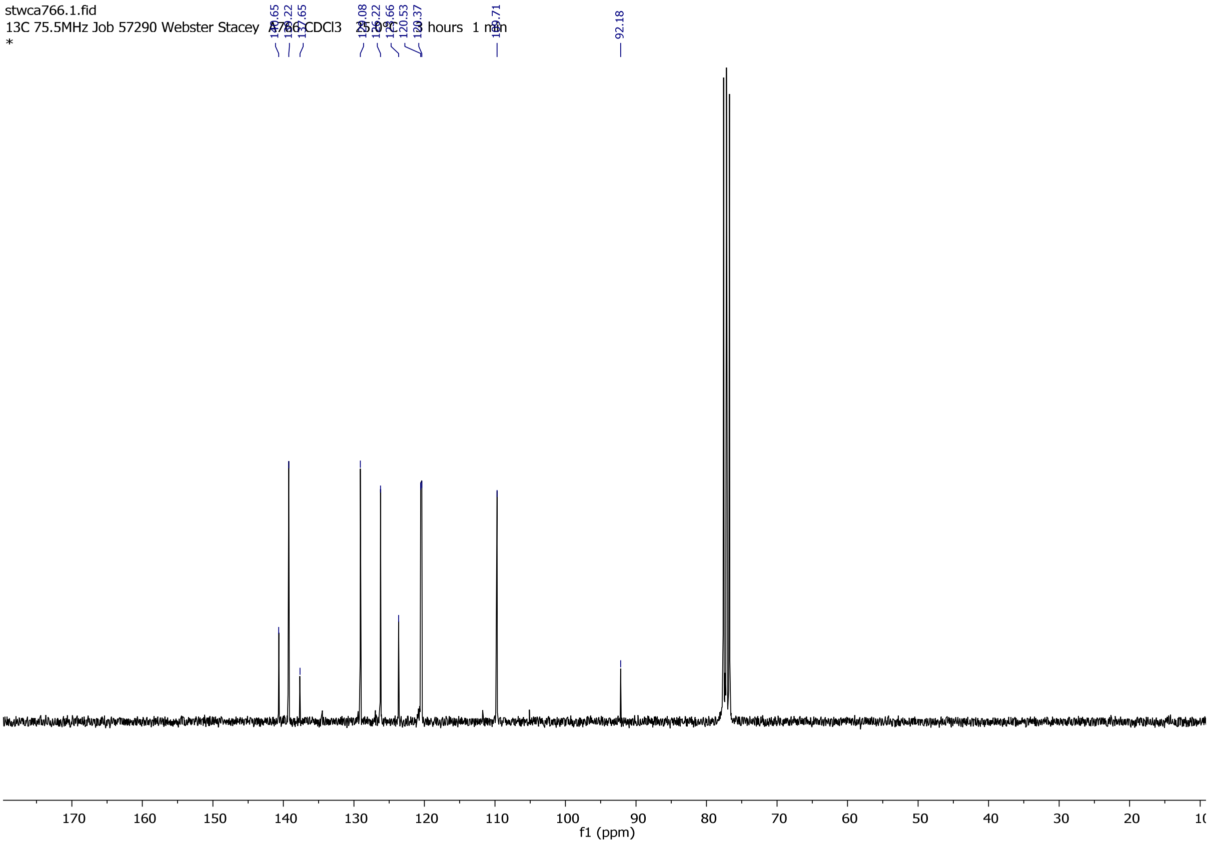
stwca766.1.fid
13C 75.5MHz Job 57290 Webster Stacey A766 CDCl3 25.0°C 3 hours 1 min
*
120.53
92.18
170
160
150
140
130
120
110
100
90
80
70
60
50
40
30
20
f1 (ppm)

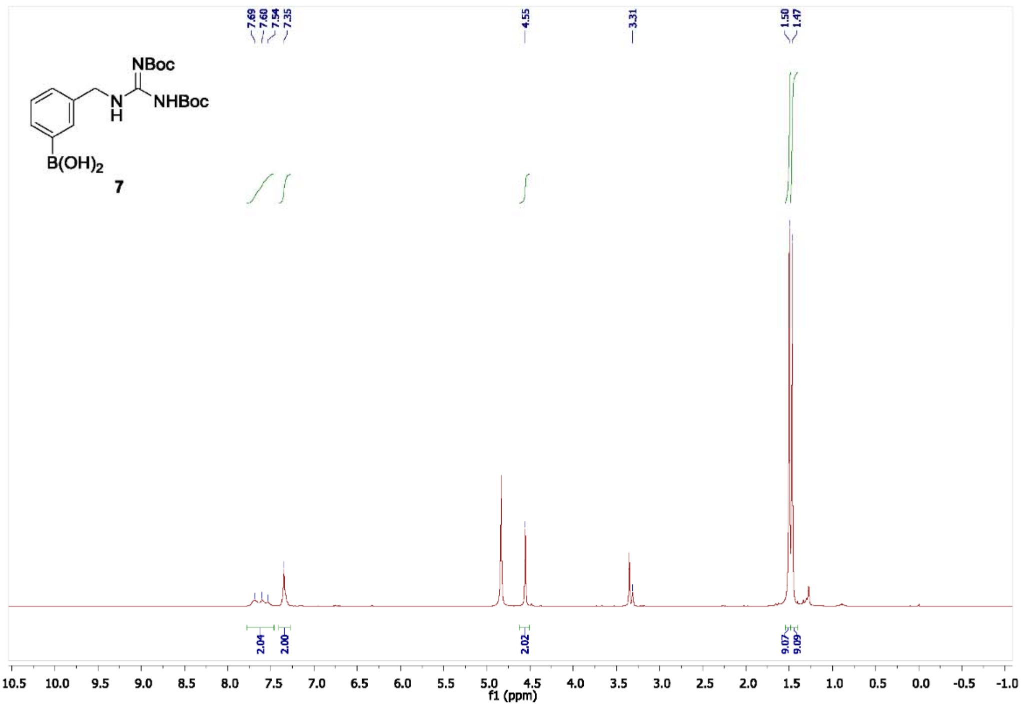

7.69
7.60
7.54
7.35
4.55
3.31
1.50
1.47
NBoc
N
H
NHBoc
B(OH)2
7
2.04
2.00
2.02
9.07
9.09
10.5 10.0 9.5 9.0 8.5 8.0 7.5 7.0 6.5 6.0 5.5 5.0 4.5 4.0 3.5 3.0 2.5 2.0 1.5 1.0 0.5 0.0 -0.5 -1.0
f1 (ppm)

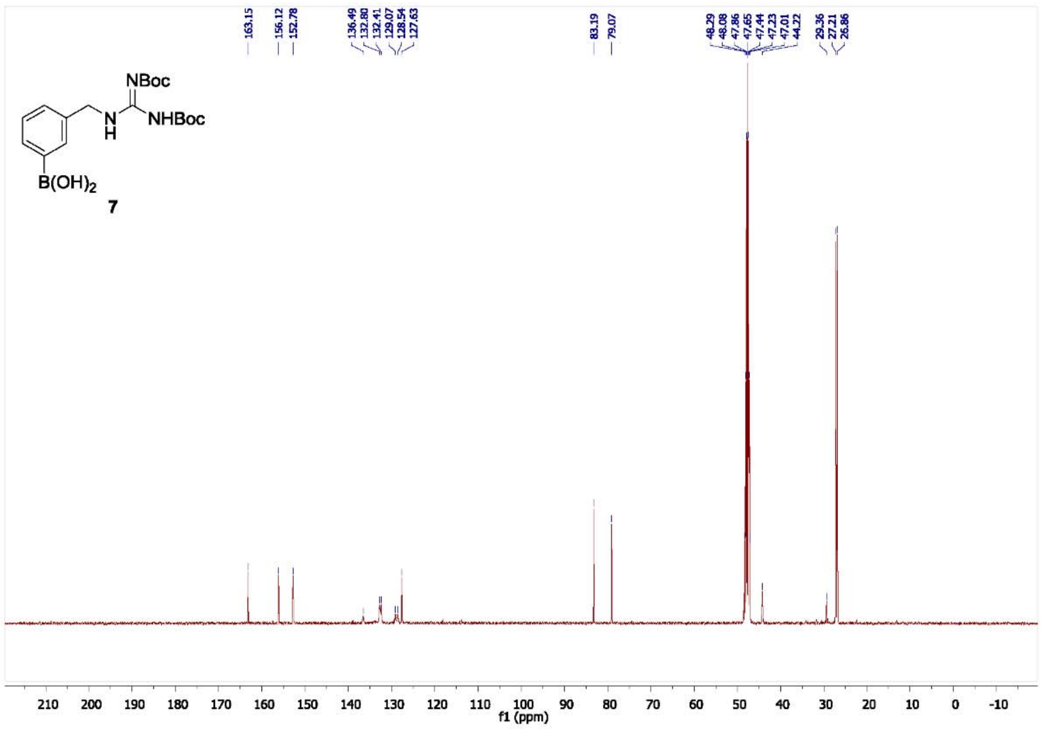

163.15
156.12
152.78
136.49
132.80
132.41
129.07
128.54
127.63
83.19
79.07
48.29
48.08
47.86
47.65
47.44
47.23
47.01
44.22
29.36
27.21
26.86
NBoc
N
H
NHBoc
B(OH)2
7
210 200 190 180 170 160 150 140 130 120 110 100 90 80 70 60 50 40 30 20 10 0 -10
f1 (ppm)

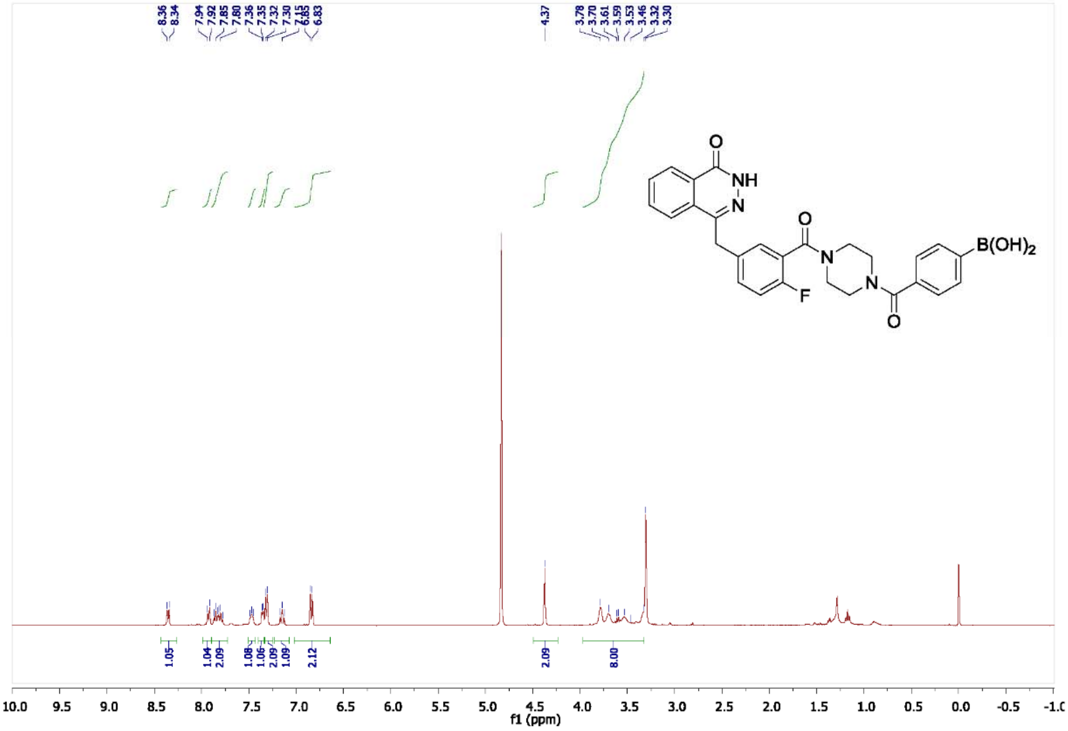
8.36
8.34
7.94
7.92
7.85
7.80
7.36
7.35
7.32
7.30
7.15
6.85
6.83
4.37
3.78
3.70
3.61
3.59
3.53
3.46
3.32
3.30
O
NH
N
O
N
N
O
F
B(OH)2
1.05
1.04
2.09
1.08
1.06
2.09
1.09
2.12
2.09
8.00
f1 (ppm)

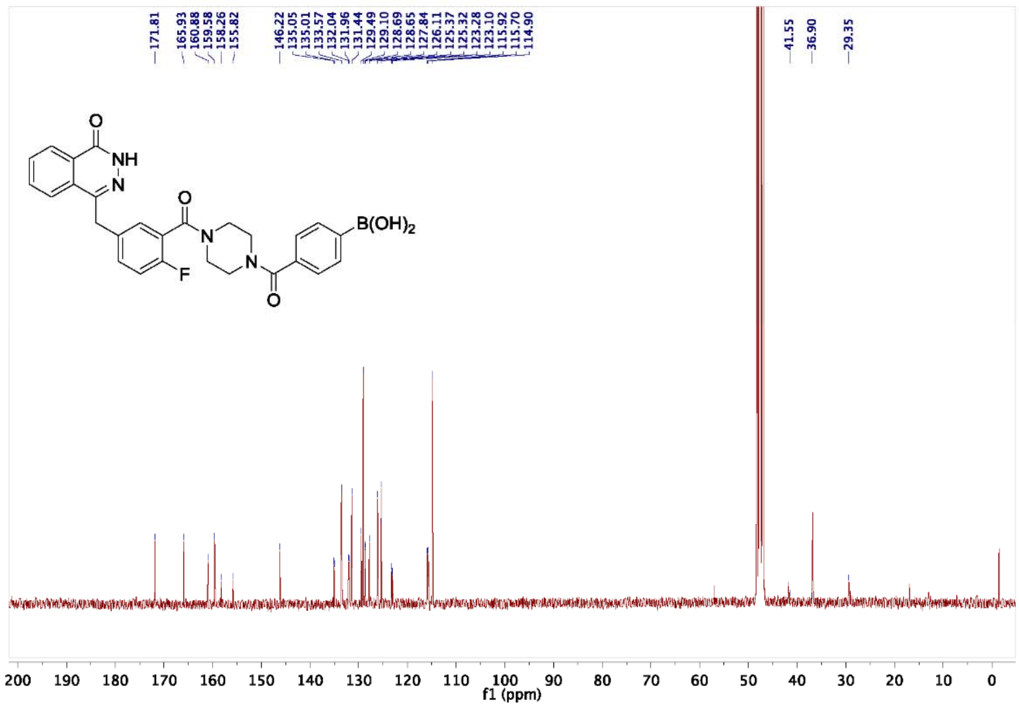
171.81
165.93
160.88
159.58
158.26
155.82
146.22
135.05
135.01
133.57
132.04
131.96
131.44
129.49
129.10
128.69
128.65
127.84
126.11
125.37
125.32
123.28
123.10
115.92
115.70
114.90
41.55
36.90
29.35
O
NH
N
O
N
N
O
F
B(OH)2
f1 (ppm)

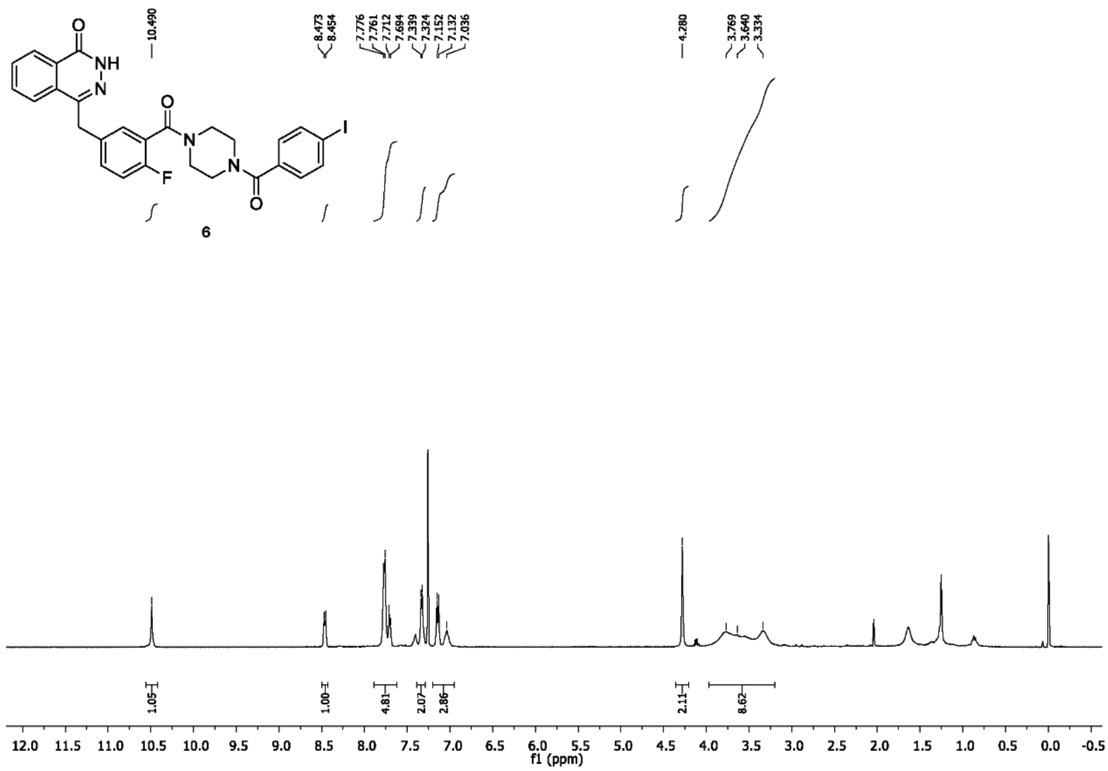
6
f1 (ppm)

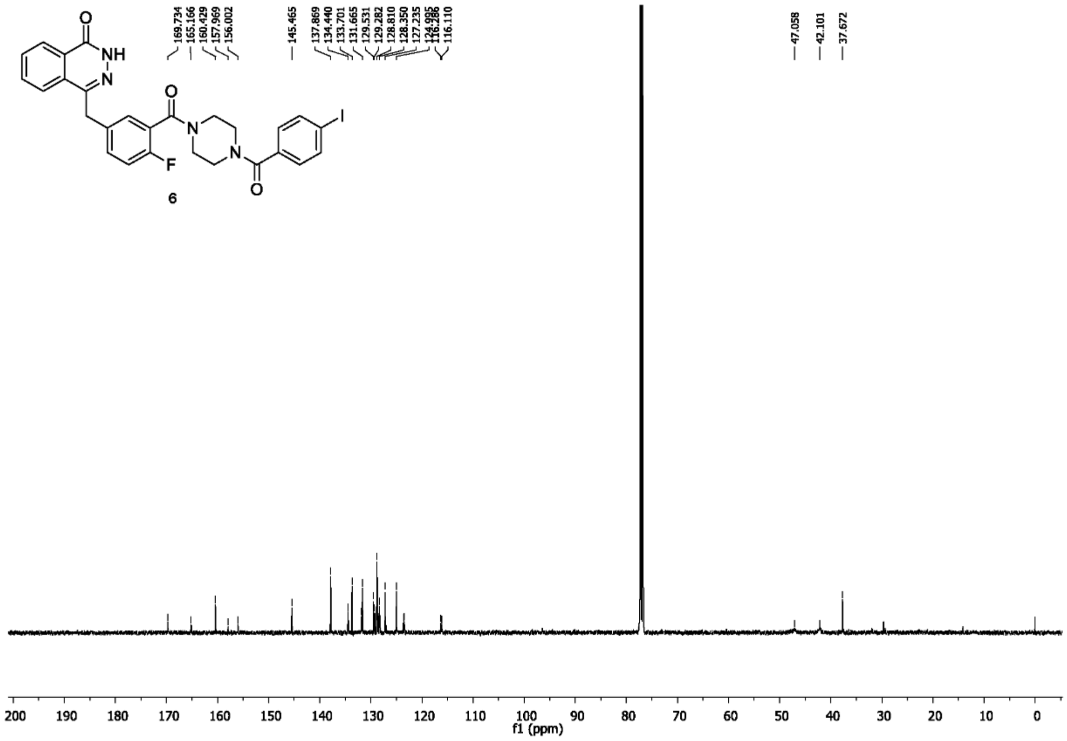
6
f1 (ppm)

6. Radio-HPLC and UV-Vis HPLC Chromatograms for all Compounds

For target compounds an overlay of the UV-Vis HPLC trace (in black) is provided along with the radio-HPLC trace (in blue):

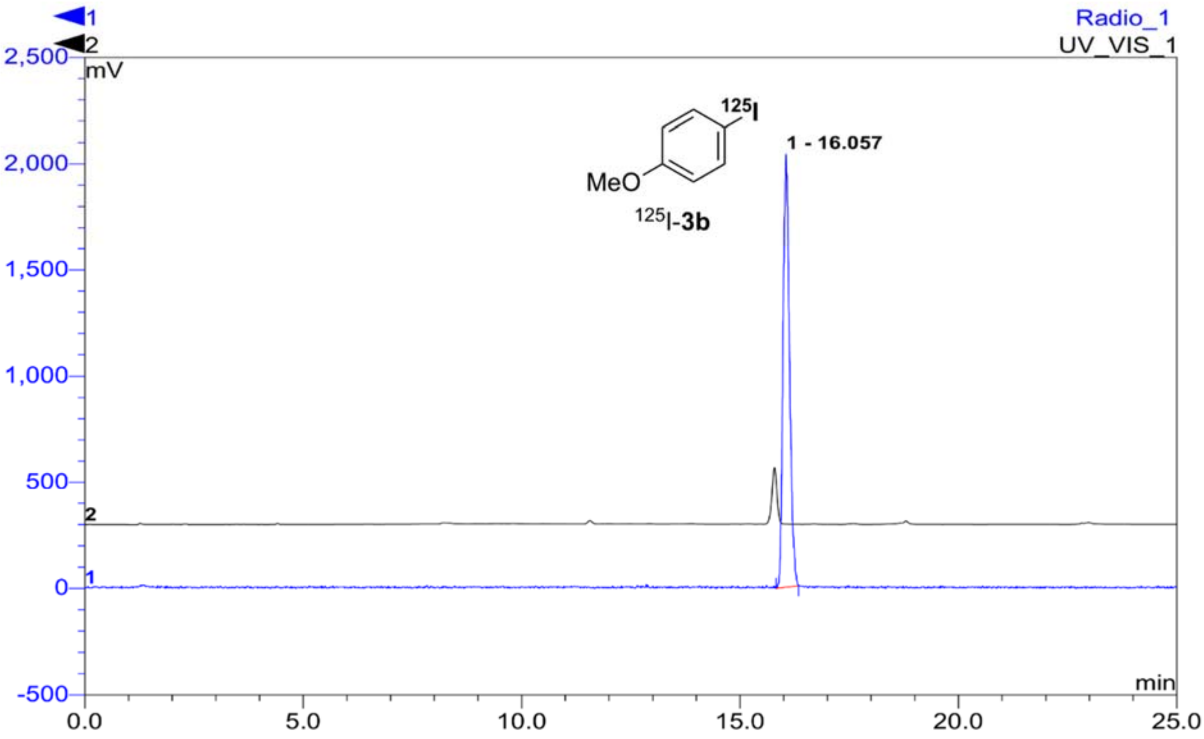

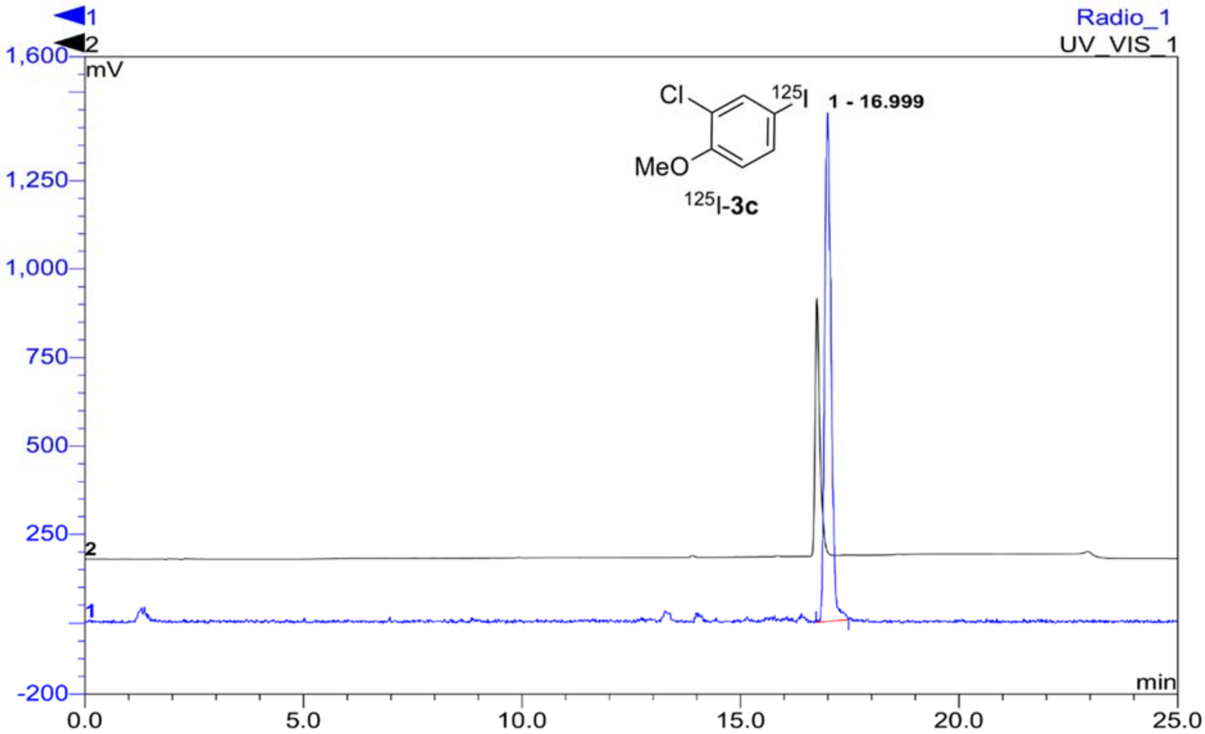
Radio_1
UV_VIS_1
mV
min
1 - 16.999
Cl
MeO
125I
125I-3c
1,600
1,250
1,000
750
500
250
-200
0.0
5.0
10.0
15.0
20.0
25.0

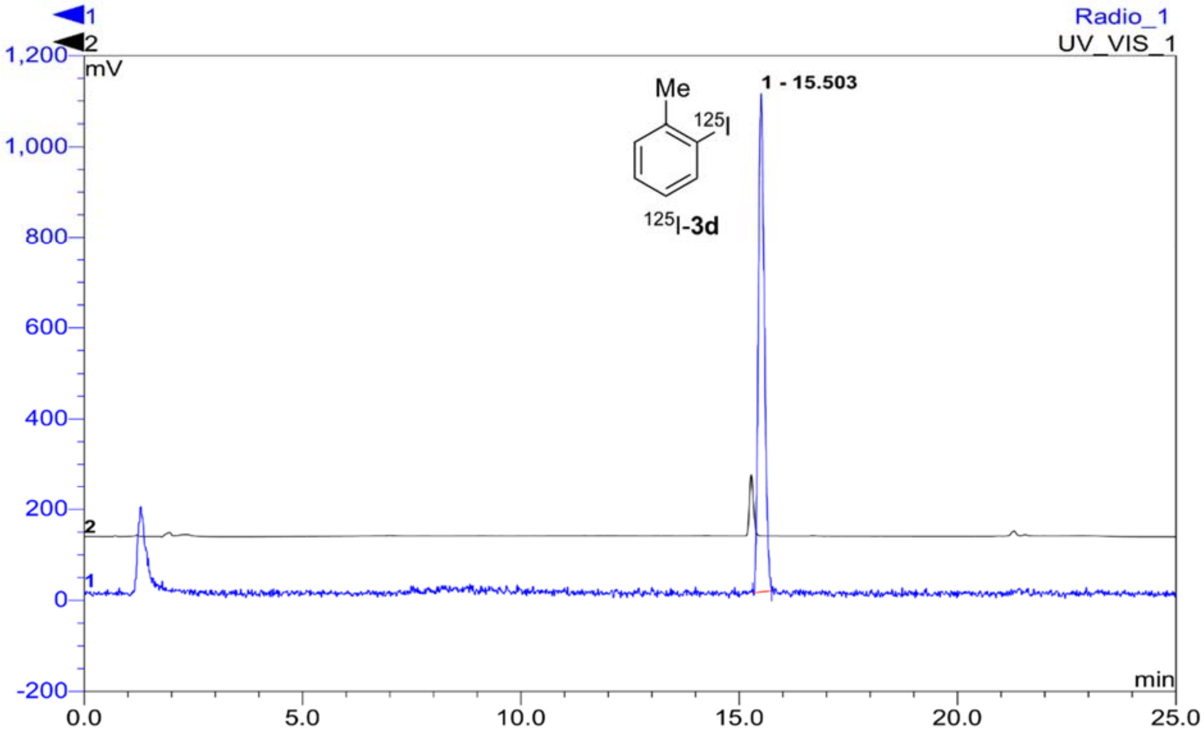
Radio_1
UV_VIS_1
mV
min
1 - 15.503
Me
125I
125I-3d
1,200
1,000
800
600
400
200
0
-200
0.0
5.0
10.0
15.0
20.0
25.0

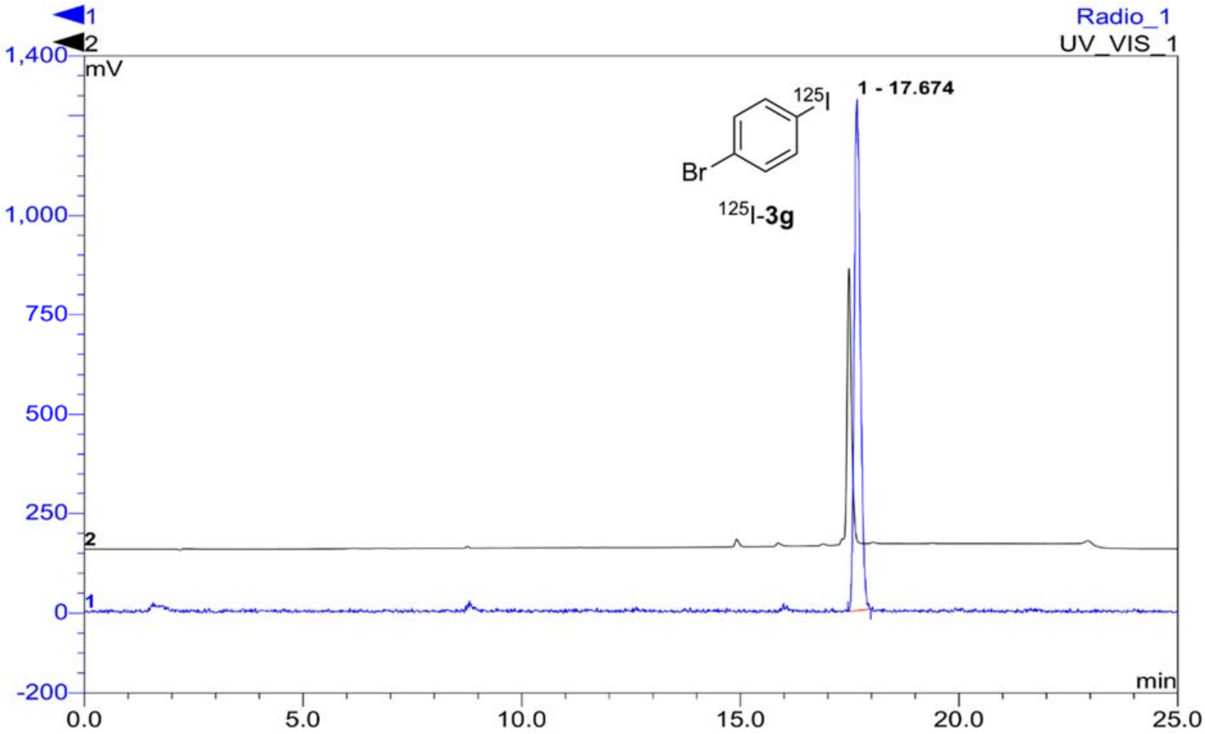
Radio_1
UV_VIS_1
mV
1 - 17.674
125I
Br
125I-3g
min
1,400
1,000
750
500
250
0
-200
0.0
5.0
10.0
15.0
20.0
25.0

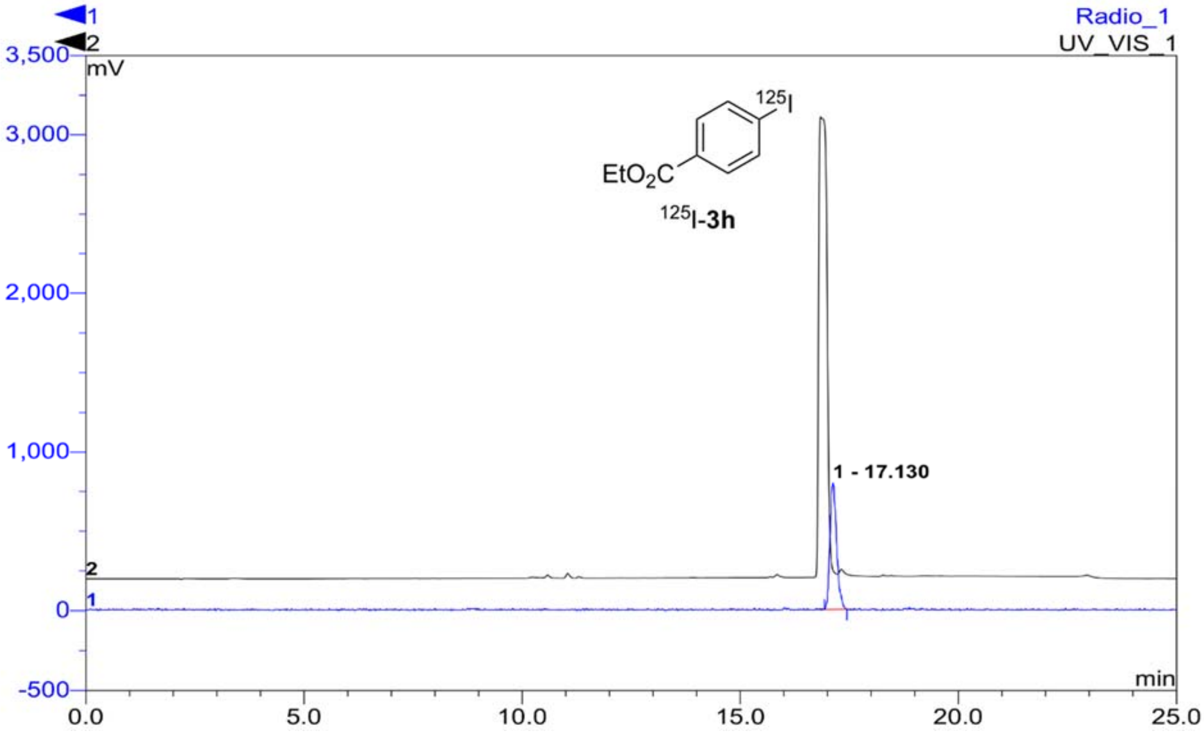
Radio_1
UV_VIS_1
mV
125I
EtO2C
125I-3h
1 - 17.130
min
3,500
3,000
2,000
1,000
0
-500
0.0
5.0
10.0
15.0
20.0
25.0

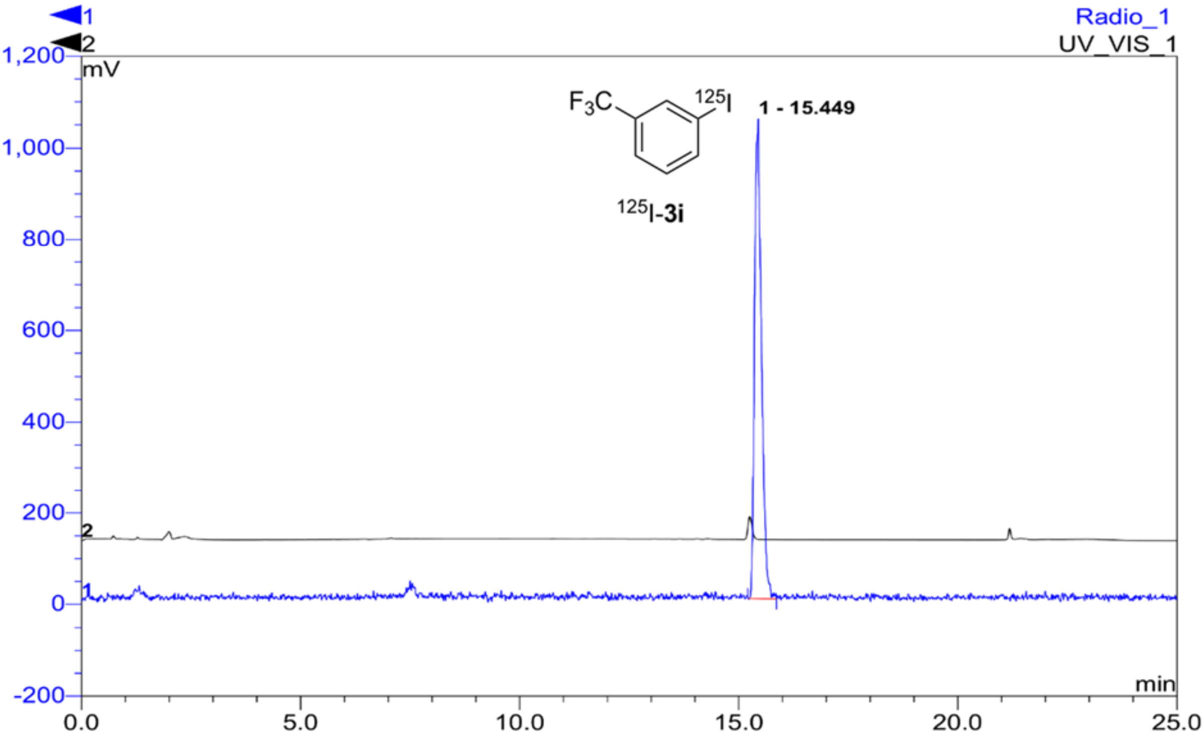
Radio_1
UV_VIS_1
mV
1,200
1,000
800
600
400
200
0
-200
F3C
125I
125I-3i
1 - 15.449
min
0.0
5.0
10.0
15.0
20.0
25.0

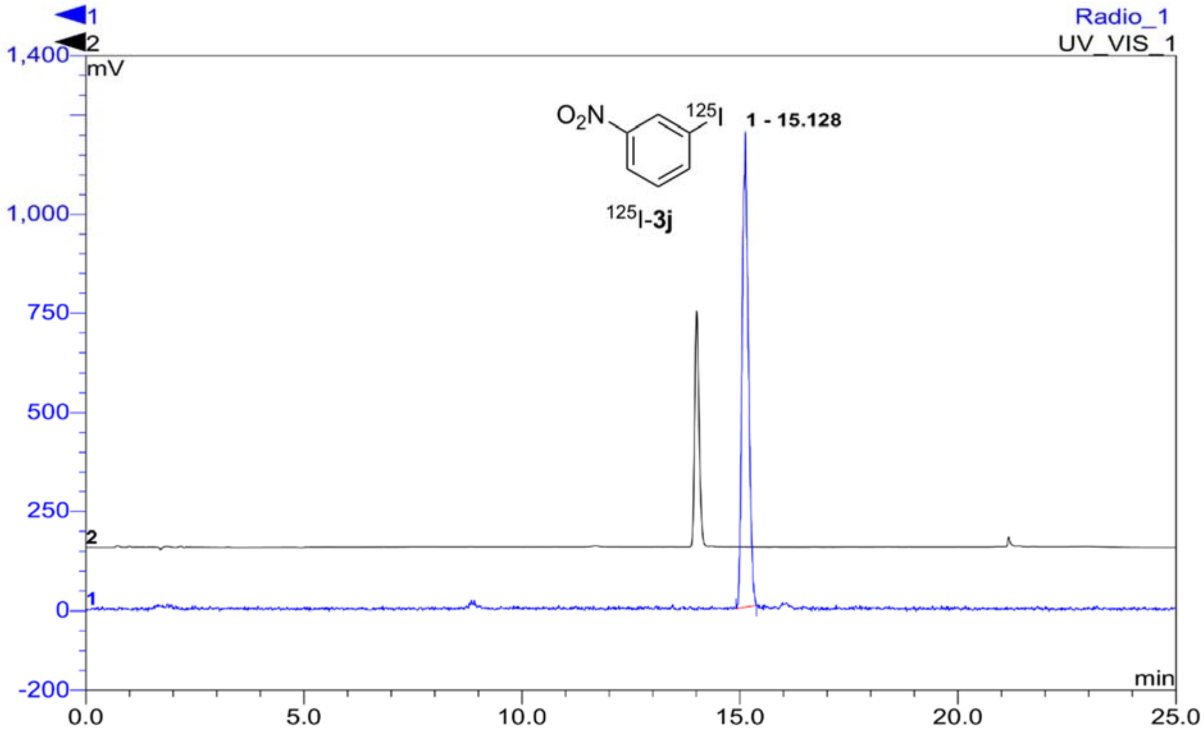
Radio_1
UV_VIS_1
mV
1,400
1,000
750
500
250
0
-200
O2N
125I
125I-3j
1 - 15.128
min
0.0
5.0
10.0
15.0
20.0
25.0

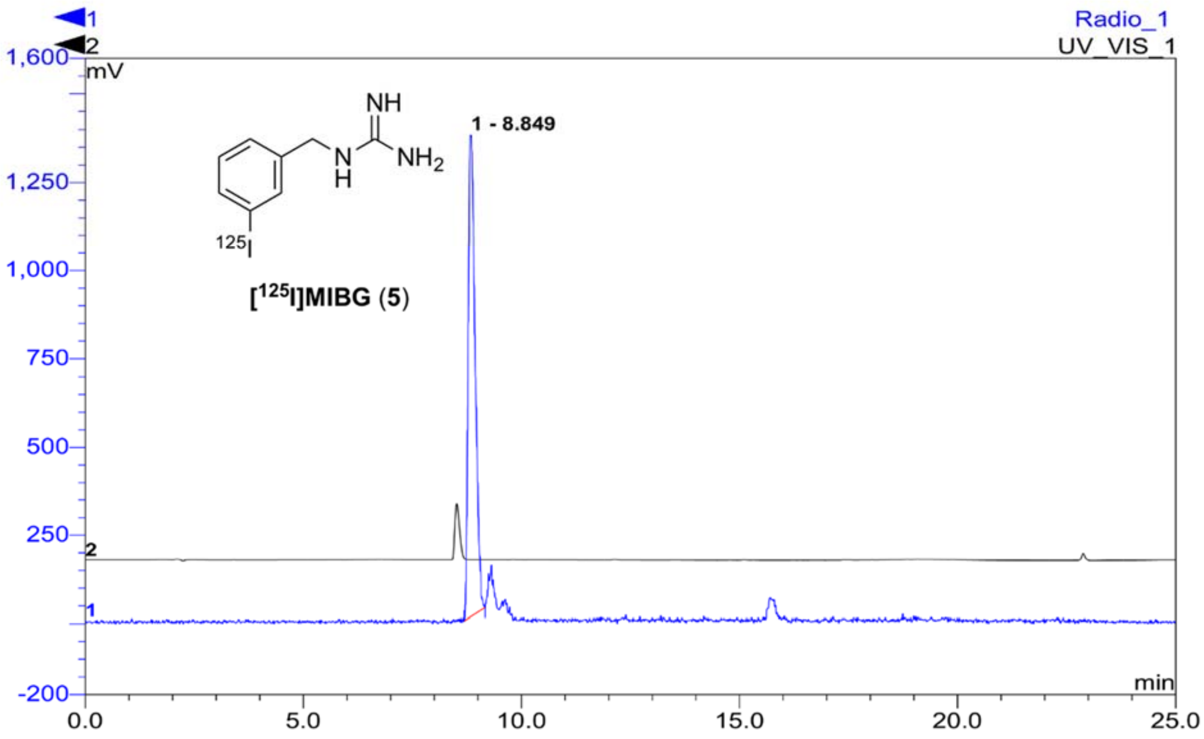
Radio_1
UV_VIS_1
mV
1 - 8.849
[125I]MIBG (5)
min
0.0
5.0
10.0
15.0
20.0
25.0
1,600
1,250
1,000
750
500
250
-200

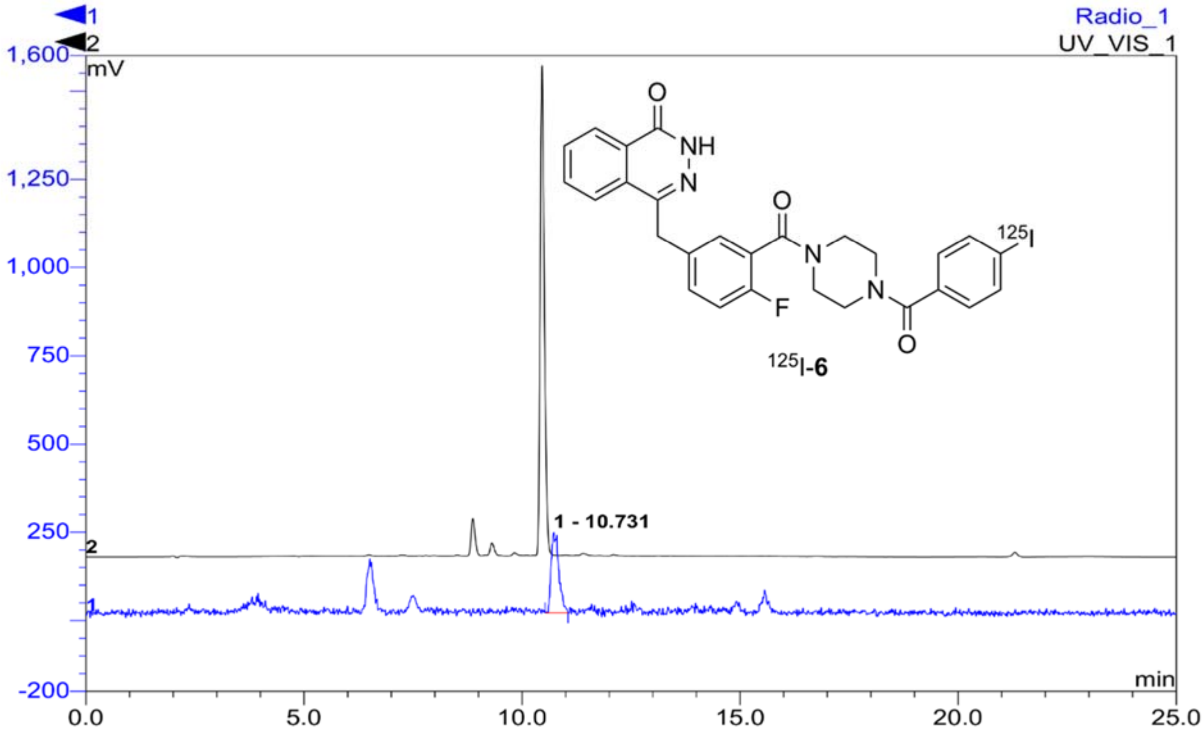
Radio_1
UV_VIS_1
mV
1 - 10.731
125I-6
min
0.0
5.0
10.0
15.0
20.0
25.0
1,600
1,250
1,000
750
500
250
-200

7. References

[1] G. Barker, S. Webster, D. G. Johnson, R. Curley, M. Andrews, P. C. Young, S. A. Macgregor, A.-L. Lee, *J. Org. Chem.* **2015**, *80*, 9807-9816.
[2] L. Niu, H. Zhang, H. Fu, *Synlett* **2014**, *25*, 995-1000.
[3] M. E. Trusova, E. A. Krasnokutskaya, P. S. Postnikov, Y. Choi, K.-W. Chi, V. D. Filimonov, *Synthesis* **2011**, *2011*, 2154-2158.
[4] R. H. Tale, G. K. Toradmal, V. B. Gopula, A. H. Rodge, R. P. Pawar, K. M. Patil, *Tetrahedron Lett.* **2015**, *56*, 2699-2703.
[5] R. D. Tilve, V. M. Alexander, B. M. Khadilkar, *Tetrahedron Lett.* **2002**, *43*, 9457-9459.
[6] N. I. Foster, N. D. Heindel, H. D. Burns, W. Muhr, *Synthesis* **1980**, *1980*, 572-573.
[7] M. Yoshida, T. Doi, S. Kang, J. Watanabe, T. Takahashi, *Chem. Commun.* **2009**, 2756-2758.
[8] D. Lebouef, J. Ciesielski, A. J. Frontier, *Synlett* **2014**, *25*, 399-402.
[9] E. B. Merkushev, N. D. Simakhina, G. M. Koveshnikova, *Synthesis* **1980**, *1980*, 486-487.
[10] F. Tramutola, L. Chiummiento, M. Funicello, P. Lupattelli, *Tetrahedron Lett.* **2015**, *56*, 1122-1123.
[11] C. Thiebes, G. K. S. Prakash, N. A. Petasis, G. A. Olah, *Synlett* **1997**, 141.
[12] E. Campaigne, W. B. Reid, J. D. Pera, *J. Org. Chem.* **1959**, *24*, 1229.
[13] H. Togo, T. Nabana, K. Yamaguchi, *J. Org. Chem.* **2000**, *65*, 8391-8394.
[14] R. R. Kadiyala, D. Tilly, E. Nagaradja, T. Roisnel, V. E. Matulis, O. A. Ivashkevich, Y. S. Halauko, F. Chevallier, P. C. Gros, F. Mongin, *Chem. –Eur. J.* **2013**, *19*, 7944.
[15] A. T. Ilagouma, J. Dornand, C. F. Liu, F. Zenone, J. C. Mani, J. M. Kamenka, *Eur. J. Med. Chem.* **1990**, *25*, 609-615.
[16] A. Mukhopadhyay, V. K. Maka, J. N. Moorthy, *Eur. J. Org. Chem.* **2016**, *2016*, 274-281.
[17] L. Wang, E. Ji, N. Liu, B. Dai, *Synthesis* **2016**, *48*, 737-750.
[18] F. Zmuda, G. Malviya, A. Blair, M. Boyd, A. J. Chalmers, A. Sutherland, S. L. Pimlott, *J. Med. Chem.* **2015**, *58*, 8683-8693.
[19] P. Zhang, R. Zhuang, Z. Guo, X. Su, X. Chen, X. Zhang, *Chem. Eur. J.* **2016**, *22*, 16783-16786.
